# Linked Data‐Driven, Physics‐Based Modeling of Pumping‐Induced Subsidence with Application to Bangkok, Thailand

**DOI:** 10.1111/gwat.13443

**Published:** 2024-10-11

**Authors:** Jenny T. Soonthornrangsan, Mark Bakker, Femke C. Vossepoel

**Affiliations:** ^1^ Department of Water Management, Faculty of Civil Engineering and Geoscience Delft University of Technology Stevinweg 1 2628 CN Delft The Netherlands; ^2^ Department of Geoscience & Engineering, Faculty of Civil Engineering and Geoscience Delft University of Technology Stevinweg 1 2628 CN Delft The Netherlands

## Abstract

Research into land subsidence caused by groundwater withdrawal is hindered by the availability of measured heads, subsidence, and forcings. In this paper, a parsimonious, linked data‐driven and physics‐based approach is introduced to simulate pumping‐induced subsidence; the approach is intended to be applied at observation well nests. Time series analysis using response functions is applied to simulate heads in aquifers. The heads in the clay layers are simulated with a one‐dimensional diffusion model, using the heads in the aquifers as boundary conditions. Finally, simulated heads in the layers are used to model land subsidence. The developed approach is applied to the city of Bangkok, Thailand, where relatively short time series of head and subsidence measurements are available at or near 23 well nests; an estimate of basin‐wide pumping is available for a longer period. Despite the data scarcity, data‐driven time series models at observation wells successfully simulate groundwater dynamics in aquifers with an average root mean square error (RMSE) of 2.8 m, relative to an average total range of 21 m. Simulated subsidence matches sparse (and sometimes very noisy) land subsidence measurements reasonably well with an average RMSE of 1.6 cm/year, relative to an average total range of 5.4 cm/year. Performance is not good at eight out of 23 locations, most likely because basin‐wide pumping is not representative of localized pumping. Overall, this study demonstrates the potential of a parsimonious, linked data‐driven, and physics‐based approach to model pumping‐induced subsidence in areas with limited data.

## Introduction

Deltas around the world are at risk of subsidence, or land sinking, due to the compressibility of alluvial and coastal deltaic sediments. These sediments are typically made up of alternating layers of sand, peat, and clay, with the latter two layers being highly compressible (e.g., Erkens et al. 2015). Subsiding megacities located in deltas are subject to increased flood risk, infrastructure damage, and seawater intrusion (e.g., Gambolati and Teatini 2015). Many studies have found a direct correlation between rising subsidence rates and declining groundwater levels (e.g., Chai et al. 2004; Phien‐wej et al. 2006; Abidin et al. 2009; Erban et al. 2014), with groundwater extraction being the main cause.

Subsidence caused by groundwater extraction is a prolonged problem, taking place over many decades. As such, it is important to take a long‐term perspective when addressing this issue (Erkens and Stouthamer 2020). For example, the coastal deltaic cities of Shanghai (China), Jakarta (Indonesia), Bangkok (Thailand), and New Orleans (USA) have experienced cumulative subsidence of 300, 200, 125, and 113 cm, respectively, in the past century due to groundwater pumping (Shen and Xu 2011; Erkens et al. 2015). Even after reducing pumping, subsidence is expected to continue due to the lag in drainage from compressible clay layers (Gambolati and Teatini 2015). Given the importance of groundwater in aquifers and clay layers to the state of deltaic cities, it is imperative to evaluate and analyze the groundwater system of these cities under current and future conditions.

Geomechanical and groundwater models are typically used to analyze the long‐term effects of subsidence caused by groundwater pumping. Physics‐based distributed groundwater models are often complicated and require a high degree of parameterization (Voss 2011a; Voss 2011b), while data‐driven methods offer an easier alternative (Bakker and Schaars 2019). Time series analysis with response functions can be used to determine the relationship between hydrogeologic forcings or drivers that affect the heads in an observation well (Collenteur et al. 2019). Forcings typically consist of precipitation, evapotranspiration, groundwater pumping, and stream stage. A time series model may be used to predict heads beyond the period of measurements, provided that (estimates of) the forcings are available. In this approach, a forcing time series is convoluted with a response function to calculate the contribution of a forcing to the head variation. Time series analysis with response functions is quick to conduct, while often giving a good fit to observations.

From a geomechanical perspective, compaction of a layer can be represented by a simple expression that approximates subsidence (Riley 1998; Erban et al. 2014; Brown et al. 2022). The expression can be utilized to model elastoplastic subsidence, where subsidence has an elastic (recoverable) and inelastic (permanent) component. The geomechanical equation can be formulated to use a time series of groundwater level variations as input to calculate subsidence. Hence, the geomechanical aspect can be based on data‐driven inputs while incorporating physical characteristics.

**Figure 1:**
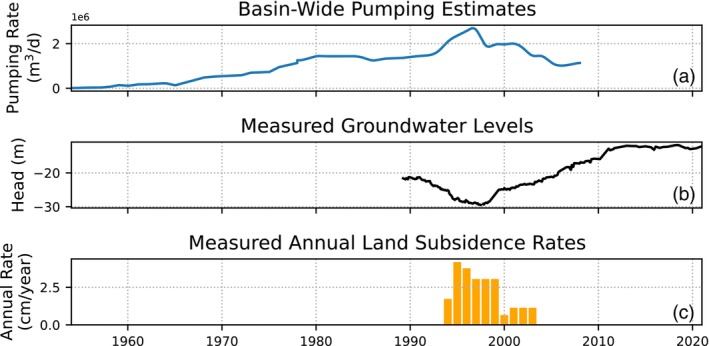
Minimal overlap between three datasets in the Chao Phraya Delta in Bangkok, Thailand which demonstrates the need for a simplified method to analyze pumping‐induced subsidence. (a) A time series of basin‐wide pumping rates from 1950 to 2007. (b) Measured head time series. (c) Observed annual subsidence rates.

Data scarcity often hinders simulation of pumping‐induced land subsidence and is a problem found in many deltas. For example, in the Mekong Delta of Vietnam, a forcing time series of pumping is available for 1991 to 2011, while groundwater and subsidence observations only span periods of 2001 to 2015 and 2006 to 2010, respectively (Minderhoud et al. 2017). Another example is the Chao Phraya Delta of Thailand. Here, yearly basin‐wide pumping data is available for the period 1950 to 2007, head measurements for 1989 to 2020, and yearly subsidence observations for 1994 to 2003 (Figure 1). Data scarcity results here in minimal overlap between the available datasets.

The objective of this paper is to develop a parsimonious approach that combines data‐driven and physics‐based modeling to simulate pumping‐induced land subsidence in deltaic areas with sparse and intermittent data. The approach is described in the first part of this paper. In the second part of this paper, the developed approach is applied to simulate and forecast pumping‐induced land subsidence in the area of Bangkok, Thailand. A short review of subsidence in the Bangkok area is given when introducing the example application.

## Developed Methods

**Figure 2:**
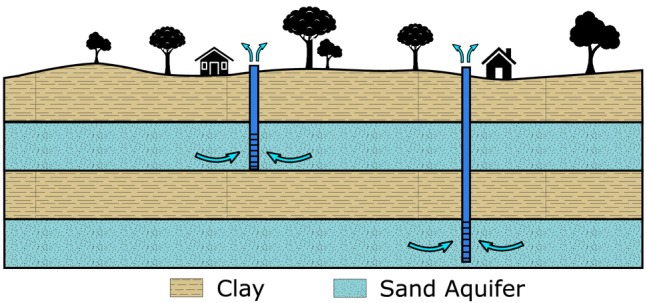
The stratigraphy of deltas often consists of alternating layers of clay and sand. Groundwater is extracted from sand aquifers located at various depths, as illustrated by the two pumping wells.

Deltaic subsurface systems are often characterized by sequences of clay and sand aquifer layers (e.g., Nile Delta, Chao Phraya Delta, Mekong Delta, Rhine Delta, Mississippi Delta; Figure 2). Analyzing historical and future subsidence caused by groundwater pumping involves simulating groundwater changes in both aquifers and clay layers. In this paper, modeling is conducted on a point scale, which is a sensible start to analyzing pumping‐induced land subsidence when dealing with data scarcity often encountered in deltas.

**Figure 3:**
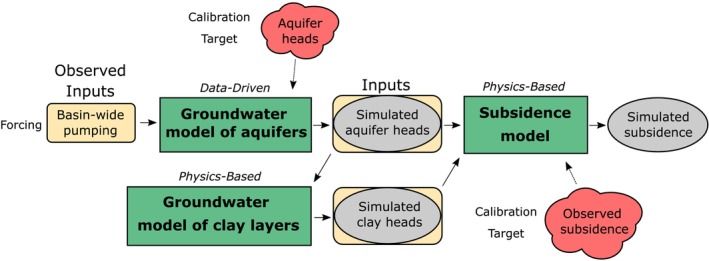
Workflow of the three‐step approach to simulate land subsidence. Yellow oval boxes represent model inputs, gray ovals represent outputs, and green boxes represent models (note that output of one model serves as input for another model). Calibration targets for the models are labeled.

A three‐step approach is developed to simulate land subsidence at observation well nests (Figure 3). First, heads in the aquifers are simulated using a data‐driven groundwater model. These heads are subsequently used as the boundary conditions to simulate the heads in the clay layers using a physics‐based groundwater model. The compaction of the layers is computed from the simulated heads using a physics‐based elastoplastic subsidence model. The combined groundwater flow model of the clay layers and the subsidence model is also commonly referred to as a consolidation model. Summation of the compaction of all layers results in the compaction‐based, vertical land subsidence at a well nest location.

### Groundwater Model of the Aquifers

Heads in the aquifer are simulated with Pastas, an open‐source Python package for time series analysis of groundwater levels (Collenteur et al. 2019). Pastas utilizes time series analysis with response functions by having input time series, or forcings, explain observed heads in monitoring wells. In time series analysis with response functions, these are called stresses. To avoid confusion with the effective stress in the aquifer, which causes subsidence, the term “forcing” is used in this paper. The following basic model structure demonstrates the usage of one forcing to explain head observations:

(1) $$h\left(t\right)=\hat{h}\left(t\right)+d+r\left(t\right)$$

where h(t) is the observed head [L], $\hat{h}\left(t\right)$ is the contribution of the forcing to the head [L], d is the base elevation of the model [L], t is time [T], and r(t) is the residual [L].

The contribution of the forcing to the simulated groundwater heads is calculated through convolution of a time series of the forcing with a response function (e.g., Collenteur et al. 2019):

(2) $$\hat{h}\left(t\right)=\int_{-\infty }^{t}Q\left(\tau \right)\theta \left(t-\tau \right)d\tau$$

where Q is the time series of the forcing, t is time [T], and θ is the impulse response function. A commonly used response function is the scaled Gamma distribution:

(3) $$\theta =A\frac{t^{n-1}}{a^{n}\Gamma \left(n\right)}e^{\frac{-t}{a}}$$

where A is a scaling factor [L/forcing dimensions], a [T] and n [−] are shape parameters, t is time [T], and Γ is the Gamma function. The scaled Gamma response function is an all‐purpose response function that is expected to perform well in many settings, including the response to distributed pumping from many wells simultaneously.

For an aquifer, a time series model with one forcing, if simulated with the scaled Gamma function, contains four parameters: A, a, and n of the response function, and the base level d. The parameters of the time series model are estimated using measured heads as a calibration target. The time series model can be used to simulate heads prior to and after the period of observed heads, provided (an estimate of) the forcing time series is available.

### Groundwater Model of the Clay Layers

A decline of heads in clay layers leads to compaction of the layer. The low permeability of the clay layers results in a delayed reaction of heads in the clay in response to changes in heads in the underlying and overlying aquifers. Clay compaction often dominates subsidence in deltas, as clay can be up to two orders of magnitude more compressible than sand. Clay compaction can have elastic and inelastic components. Rebound can occur if previous compaction had some elastic components, but often, compaction is inelastic (Gambolati and Teatini 2021). Inelastic compaction is described by the permanent physical rearrangement of sediment grains (Hoffmann et al. 2003). An elastoplastic model takes elastic and inelastic subsidence into consideration using the previous maximum effective stress, or preconsolidation stress. If the preconsolidation stress is exceeded, the compressibility and specific storage of clays change (Riley 1969). In terms of groundwater, preconsolidation stress can be defined as inversely proportional to the preconsolidation head, i.e., the maximum effective stress occurs when the groundwater head is at its minimum (Hoffmann et al. 2003). The specific storage term for elastic deformation is used when groundwater heads are above the preconsolidation head (the previous groundwater minimum), while the specific storage term for inelastic deformation is used when groundwater heads fall below the preconsolidation head.

The head in a clay layer is modeled with the one‐dimensional diffusion equation (e.g., Hoffmann et al. 2003):

(4) $$\frac{\partial^{2}h}{\partial z^{2}}=\frac{S}{K_{v}}\frac{\partial h}{\partial t}\;S\left\{\begin{array}{c} S_{e}\;\text{for}\;h>h_{p}\\ S_{v}\;\text{for}\;h\leq h_{p} \end{array}\right.$$

where S is the skeletal specific storage ($S_{e}$: elastic or $S_{v}$: inelastic value depending on whether the preconsolidation head $h_{p}$ is exceeded) [1/L], $K_{v}$ is the vertical hydraulic conductivity of the clay layer [L/T], z is the vertical coordinate [L], and t is time [T].

Simulated groundwater levels in the aquifers are used as the boundary conditions for the simulation of the head in the clay layers. The heads above the clay layer may differ from the heads below the clay layer; the upper boundary of the top clay layer is approximated as a no‐flow boundary. Each clay layer is discretized in N model layers of equal thickness (Section S1.2), and the diffusion equation is solved using an implicit finite difference scheme. The implicit approach has the advantage of being unconditionally stable, independent of the size of the time steps (Wang and Anderson 1995).

Heads in the clay layers are simulated from the start of substantial pumping. Since heads in clay layers are rarely known, the initial heads in a clay layer can be linearly interpolated from the steady state heads of the overlying and underlying aquifers prior to the start of substantial pumping. The groundwater model of each clay layer contains three parameters: the specific storage $S_{e}$ (elastic) and $S_{v}$ (inelastic) and the vertical hydraulic conductivity $K_{v}$.

### Subsidence Model

Pumping‐induced land subsidence is modeled using an expression that approximates layer compaction over a time period based on groundwater drawdown and layer properties (Riley 1969; Erban et al. 2014; Brown et al. 2022). The elastoplastic model uses an elastic specific storage term if groundwater head is above the preconsolidation head and an inelastic term if it is below (Hoffmann et al. 2003). Sand layers are approximated as homogeneous and only elastic compaction is used; the inelastic compaction of sand is often found negligible compared to that of clay. For clay, the head changes above the preconsolidation head results in small elastic compaction. Below the preconsolidation head, compaction of clay is inelastic.

An approximation made in the subsidence model is that compaction is only occurring in the vertical z‐direction. The use of a one‐dimensional model is applicable if the aquifer system has an areal extent much larger than the vertical depth in question. Groundwater pumping is not a localized occurrence but is applied over the entire basin. Thus, sediment compaction migrates to the ground surface and behaves mechanically in a one‐dimensional manner (Gambolati and Teatini 2021).

The subsurface is divided in $M_{c}$ clay layers and $M_{a}$ aquifer layers; clay layers are further discretized into N model layers of equal thickness, while the aquifer layers are represented by a single model layer. Time is discretized into equal periods Δt. Total land subsidence D during period Δt is the sum of the compaction of all the model layers during the period:

(5) $$D=\sum_{n=1}^{M_{a}+{\mathrm{NM}}_{c}}\Delta b_{n}$$

If layer n is an aquifer, the change in thickness $\Delta b_{n}$ of model layer n during period Δt is computed as:

(6) $$\Delta b_{n}=S_{e_{n}}b_{n}\Delta h_{n}$$

If layer n is a clay layer, the change in thickness $\Delta b_{n}$ of model layer n is computed as:

(7) $$\Delta b_{n}=S_{n}b_{n}\Delta h_{n}\;S_{n}\begin{array}{l} \left\{\begin{array}{c} S_{e_{n}}\;\text{for}\;h_{n}>h_{p}\\ S_{v_{n}}\;\text{for}\;h_{n}\leq h_{p} \end{array}\right. \end{array}$$

where $b_{n}$ is the initial full thickness of model layer n [L], $\Delta h_{n}$ is the change in head in model layer n during period Δt [L], and $S_{n}$ is the specific storage of model layer n ($S_{e_{n}}$: elastic or $S_{v_{n}}$: inelastic) [1/L].

The subsidence model of each aquifer layer contains only one parameter: the specific storage $S_{e}$ (elastic). The subsidence model of each clay layer contains two parameters: the specific storage $S_{e}$ (elastic) and $S_{v}$ (inelastic), which are the same values as the parameters of the groundwater model of each clay layer. The sets of parameters for the clay and subsidence models are estimated simultaneously.

### Subsidence in Bangkok, Thailand

The described approach is applied to model and forecast pumping‐induced land subsidence in Bangkok, Thailand. The capital city of Bangkok continues to encounter many subsidence issues, as it is located on a delta. Groundwater started to be a major source of the public water supply in 1954 when groundwater pumping was around 8360 m^3^/d (Gupta and Babel 2005). Lagging piped‐water supply, lack of widespread infrastructure, and growing water demand led to additional private and largely unregistered groundwater pumping (Japan International Cooperation Agency 1995; Gupta and Babel 2005; Babel et al. 2006). In the 1980s, Bangkok underwent major urbanization and population growth, leading to increased groundwater usage and average pumping rates of 1.4 million m^3^/d (Endo 2011). Substantial groundwater decline resulted in subsidence of up to 12 cm per year in the early 1980s (Aobpaet et al. 2013). After identifying groundwater pumping as a main driver for the subsidence, the Thai government introduced regulatory measures in the 1980s to control groundwater pumping, which took time to implement but eventually resulted in stabilized or reduced subsidence rates (Buapeng and Foster 2008). Subsidence continues today, albeit at smaller rates, with an average areal rate of around 1 cm per year (Lorphensri et al. 2016).

Many projects have investigated subsidence caused by groundwater pumping in Bangkok from geomechanical and hydrogeological perspectives (e.g., Nutalaya et al. 1986; Japan International Cooperation Agency 1995; Babel et al. 2006; Chulalongkorn University Faculty of Engineering 2009). Saowiang and Giao (2020) and Giao et al. (2013) used geomechanical finite element models to simulate land subsidence due to compaction of the upper 80 m of the subsurface from groundwater drawdown at specific sites throughout Bangkok. Chulalongkorn University Faculty of Engineering (2009) used spatially distributed physics‐based groundwater and subsidence modeling to analyze the contributions of various drivers to historical subsidence as well as forecast subsidence to 2027. The study found that roughly 70% of total subsidence is attributed to groundwater pumping and 30% to building load. In this paper, the new parsimonious, linked data‐driven and physics‐based approach, described in the previous, is applied, which is well‐suited to deal with sparse and intermittent data. The approach is capable of simulating pumping‐induced subsidence at an individual well nest, based on the measured groundwater dynamics, subsidence rates at or near the well nest, and an estimate of basin‐wide pumping.

### Study Site

**Figure 4:**
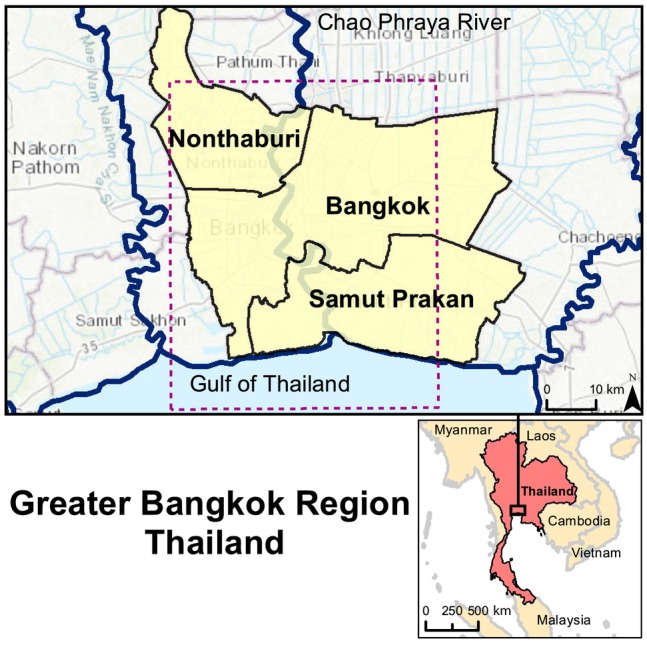
The study area consisting of the Bangkok, Nonthaburi, and Samut Prakan provinces (OpenStreetMap contributors 2017). The area denoted by the dashed purple box represents the area shown in Figures 7, 9, 10, 12, and 14.

The province of Bangkok, Thailand has an official area of 1570 km^2^ with a population of about 10.35 million people (United Nations 2021). For the purpose of this study, the greater Bangkok region refers to the 3156 km^2^ area consisting of the provinces of Bangkok, Nonthaburi, and Samut Prakan (Figure 4). The region is situated on the Lower Central Plain, or specifically the Chao Praya River Delta, which is a large flat delta consisting of fluvial and marine deposits. The Chao Praya River flows through the study area and discharges into the Gulf of Thailand 25 km south of the city.

**Figure 5:**
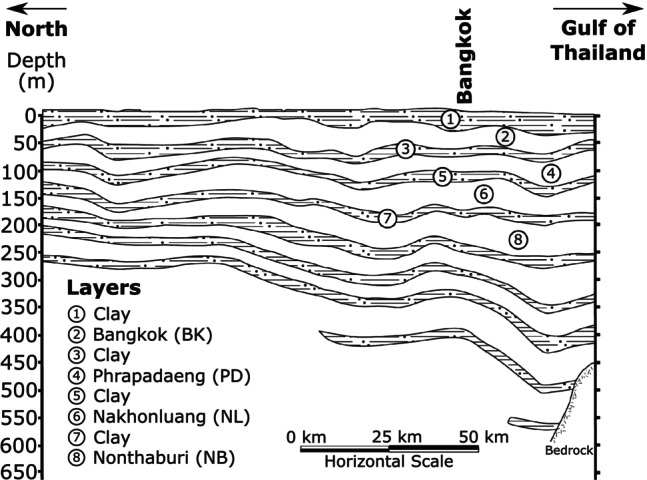
A schematic north‐south cross section of the Bangkok Plain indicating the generalized clay and aquifer layering (after Phien‐wej et al. 2006).

Past alluvial, fluvial, and deltaic processes are responsible for the 2000 m of complex sequences of Pleistocene and Holocene deposits underlying the Chao Praya River Delta (Sinsakul 2000). Eight main aquifers are recognized in the upper 600 m of unconsolidated sediments. These are confined aquifers of sand and gravel that are interbedded with clays and separated by clay aquitards (Figure 5). The prevalence of clay layers throughout the Bangkok subsurface makes the city highly susceptible to subsidence.

**Figure 6:**
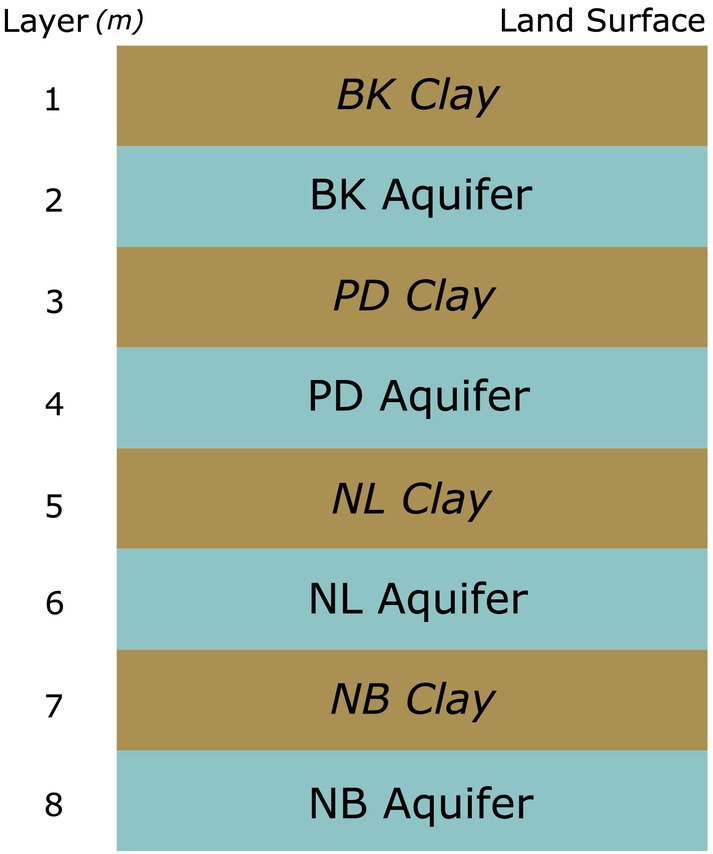
Only the four uppermost confined aquifers and their respective confining clay layers are considered. In this paper, clay layers are named after the aquifers they are confining.

The focus of this study is on the four shallowest aquifers and the clay layers between them (Figure 6). The first 50 m seemingly contributes 30% to 50% of land subsidence, and the remaining percentages are likely due to the three next shallowest and highly pumped aquifers (Nutalaya et al. 1986; Phien‐wej et al. 2006). The Bangkok (BK) aquifer is the uppermost aquifer within the first 50 m depth and is about 20 to 30 m thick (Phien‐wej et al. 2006). Most pumping occurs within the second Phra Pradaeng (PD) aquifer, the third Nakorn Luang (NL) aquifer, and the fourth Nonthaburi (NB) aquifer (Phien‐wej et al. 2006). The PD aquifer is approximately within the 50 to 100 m depth zone, NL within 100 to 150 m, and NB within 150 to 200 m. Pumped groundwater is used for domestic, agricultural, and industrial purposes, of which industrial use is the most significant (Babel et al. 2006). Groundwater is typically older than 10,000 years, suggesting that natural recharge is a slow process (Sanford and Buapeng 2012). Significant recharge to the aquifers is limited to the edge of the basin.

### Data

Basin‐wide yearly pumping rates are the only forcing. This means that it is assumed that the relative variation in pumping rates is the same for all aquifers. The effects of precipitation, evapotranspiration, and return flow are neglected in this study and are considered negligible with respect to pumping. Endo (2011) and Babel et al. (2006) provided estimates of basin‐wide pumping rates, which are combined into an estimate of basin‐wide pumping for 1954 to 2007. Pumping rates for 2008 to 2020 are unknown and are adjusted manually during the calibration process. The Thai government reports unofficial pumping rates between 490,000 and 610,000 m^3^/d for 2016 to 2021. The average of this range is used as an initial pumping estimate for 2007 to 2020 in the time series models. Ultimately, the pumping rates for 2009 to 2020 are calibrated at a constant rate of 500,000 m^3^/d after a linear decline from 2007 to 2009.

Head observations in the aquifers are obtained from the Thai Department of Groundwater Resources (DGR; Department of Groundwater Resources 2021). Depth to water observations are converted to groundwater heads using the Climate Central's CoastalDEM v2.1 90 m resolution of land surface (Kulp and Strauss 2021). Head measurements are available for the period 1994 to 2020 for most wells. The average head of the BK, PD, NL, and NB aquifer are −14, −18, −26, and −27 m (standard deviation: 3.9, 5.0, 8.5, and 8.1 m), respectively; the statistics of groundwater data and a typical well nest configuration are shown in Section S1.1.1.

For modeling of groundwater of the clay layers and subsidence, thicknesses of all layers are approximated from geologic logs provided by the Thai DGR (Table S26). Benchmark level measurements serve as land subsidence observations (Royal Thai Survey Department 2021). Every well nest, except for one, has benchmark levels measured within 500 m (the exception is well nest BKK036 which has a station within 1 km). The benchmark levels are located at 1 m depth, which represent subsidence that occurs below 1 m and serve as the best estimate of land subsidence at those locations. Yearly benchmark levels are available for the period 1992 to 2003 for most stations. If data was missing for certain years, the next available measurement represents the cumulative subsidence rate that occurred over the years where measurements were not taken. The cumulative subsidence rate was averaged for these years as the best estimate of observed annual subsidence rate. The average subsidence rate for all leveling stations is 2.1 cm/year with a standard deviation of 2.2 cm/year. Total range is approximately 16.2 cm/year. Statistics for every benchmark leveling station are provided in Data S1.2.

The Hampel filter was applied to identify possible outliers in the subsidence observations (Pearson 2002). Observations at eight out of 23 well nests were determined to have outliers and will be marked when presenting results. The outliers are characterized as sudden spikes in subsidence rate measurements (e.g., 13 to 14 cm/year spikes in 2005 to 2006 for well nest BKK005, BKK011, BKK020, and BKK027; see Section S1.5). After outlier detection, outliers were compared to observations of groundwater heads and pumping, and there are no corresponding decreases in groundwater heads or increases in groundwater pumping. Outliers are attributed to either unobserved processes or are incorrect measurements. Instrument error is not known.

### Modeling Approach

**Figure 7:**
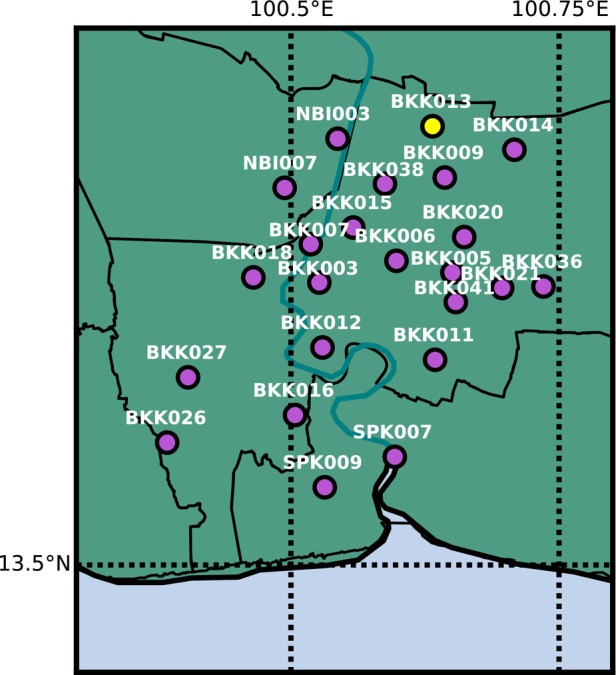
The 23 observation well nests throughout the study area. The yellow dot is the location of BKK013, which is used in Figures 8, 11, and 13.

Twenty‐three observation well nests were selected for groundwater estimation with the time series model based on head availability (Figure 7). The time series models start in 1950, which is the start of substantial pumping, with most head observations starting in the 1980s. Most well nests do not have head observations in all four aquifers; models are only created for the aquifers with available observations. A total of 66 time series models are created: 5 for BK, 20 for PD, 19 for NL, and 22 for NB.

Groundwater heads are needed in all four aquifers to model the head in the clay layers and, subsequently, land subsidence. Most well nests lack head measurements in the BK aquifer and some in the PD aquifer. Complete observation sets at well nests with all four aquifers reveal similar patterns and trends between the shallowest two and deepest two aquifers. If the BK aquifer is missing for a particular well nest, the PD model is used as a proxy. If the NL aquifer is missing, the NB model is used as a proxy. If only a model for a single aquifer is available, it is used as a proxy for the three missing aquifers.

All time series models are used to simulate heads starting in 1950, which is the start of substantial pumping. The simulated heads in the aquifers serve as the boundary conditions to simulate the heads in the clay layers. The groundwater models for the clay layers also start simulation in 1950. The initial heads in the clay layers are linearly interpolated from the steady state heads of the overlying and underlying aquifers, which in turn are obtained from the time series model (the value of parameter d in Equation 1). Running these two models prior to the start of the subsidence model in 1978 is essential to allow clay layers to have realistic heads. The heads simulated in the aquifers and clay layers serve as input for the subsidence model.

### Calibration Approach

With the scaled Gamma response function, a time series model of each aquifer contains four parameters, which are estimated using least squares as implemented in Pastas. The calibration period for the time series models is 1978 to 2005, and the validation period is 2005 to 2020, while some wells have data for only part of the periods. Model fit is visualized once the parameters are optimized by the time series model. Simulated heads in the aquifers are then used as inputs for the groundwater models of the clay layers and the subsidence models.

The groundwater model of each clay layer requires three parameters: the elastic and inelastic specific storage and the vertical hydraulic conductivity. With no head observations in the clay layers for calibration, the parameters of the clay and subsidence models are estimated simultaneously. Subsidence rates are calibrated for the time period 1978 to 2003, which starts at the earliest available benchmark measurement and consists of both intense and reduced pumping. However, measurements at most well nests are only taken for a few years from 1990 to early 2000s.

At each well nest location, the linked models require four parameter sets consisting of four values each (one for each layer). The four parameter sets are: the elastic specific storage of the aquifer sand layers, the elastic specific storage of the clay layers, the inelastic specific storage of the clay layers, and the vertical hydraulic conductivity of the clay layers. Hence, the total number of parameters at each well nest location is 16. The elastic storage values for sand are not frequently reported in literature, but generally, the range for elastic specific storage of sand tends to be an order of magnitude smaller than that of clay (Batu 1998; Chen et al. 2016). This (rough) approximation is made for the elastic specific storage values of the sand layers. Each parameter set for clay layers for each well nest is calibrated using only one constant that affects every layer equally, as explained in the following. This reduces the number of calibration parameters to three for each well nest.

Initial estimates of vertical hydraulic conductivity of each clay layer are assigned the average of the ranges reported by Japan International Cooperation Agency (1995). During calibration, the vertical hydraulic conductivity $K_{v_{m}}$ of clay layer m is adjusted using constant α:

(8) $$K_{v_{m}}=\alpha K_{v_{m,0}}$$

where $K_{v_{m}}$ is the calibrated vertical hydraulic conductivity of clay layer m [1/L], $K_{v_{m,0}}$ is the initial vertical hydraulic conductivity value of clay layer m, and α is the calibration constant, which is the same for all clay layers.

Inelastic specific storage is approximated as constant within each clay layer but decreases with clay layer depth (midpoint). The following empirical model is used to describe the decrease of inelastic specific storage $S_{v}$ with depth (Kuang et al. 2021):

(9) $$\ln\frac{S_{v_{m}}}{S_{\mathrm{sr}}}=\ln\frac{S_{s0}}{S_{\mathrm{sr}}}{\left(1+\frac{z_{m}}{1000}\right)}^{-\lambda }$$

where $S_{v_{m}}$ is the inelastic specific storage of clay layer m [1/L], $S_{\mathrm{sr}}$ is the residual specific storage [1/L], $S_{s0}$ is the inelastic specific storage at ground surface [1/L], λ is the decay index [−], and $z_{m}$ is the midpoint depth below ground surface of clay layer m [L]. Inelastic specific storage at ground surface, residual specific storage, and λ are chosen such that inelastic specific storage in the deepest layer beneath Bangkok are approximately half that of ground surface values (Erban et al. 2014), so that $S_{s0}$ = 5.5e−4 m^−1^, $S_{\mathrm{sr}}$ = 1e−4 m^−1^, and λ = 0.5.

During calibration, the initial inelastic specific storage $S_{v_{m,0}}$ of clay layer m (calculated using Equation 9) is adjusted using constant β:

(10) $$S_{v_{m}}=\beta S_{v_{m,0}}$$

where $S_{v_{m}}$ is the calibrated inelastic specific storage of clay layer m [1/L], $S_{v_{m,0}}$ is the initial inelastic specific storage value of clay layer m (Equation 9), and β is the calibration constant, which is the same for all clay layers.

Elastic specific storage tends to be 20 to 100 times less than inelastic storage (Riley 1998). Elastic storage $S_{e_{m}}$ of clay layer m is computed as

(11) $$S_{e_{m}}=\frac{S_{v_{m}}}{\gamma }$$

where $S_{e_{m}}$ is the elastic specific storage of clay layer m [1/L], $S_{v_{m}}$ is the inelastic specific storage of clay layer m [1/L], and γ is the calibration constant that varies between 20 and 100 [−].

The parameters of the clay and subsidence models are calibrated using the sparse data available. Calibration constants α and β are adjusted for each well nest while maintaining that calibrated vertical hydraulic conductivity and inelastic specific storage are within ranges determined from local consolidation and pumping tests reported by Japan International Cooperation Agency (1995) (Section S1.3.2). For each well nest, γ is adjusted between 20 and 100. Thus, three calibration constants are adjusted for each well nest. The calibration constants are manually adjusted to fit simulated subsidence to observations by approximately minimizing the root mean square error (RMSE).

## Results

The following section contains the results of the time series models of the heads in the aquifers and the land subsidence models.

### Heads in the Aquifers

Time series models were created, calibrated, and validated for all 66 wells at 23 well nests. Calibration and validation results are summarized in Table 1. The optimized parameters of the time series models are reported in Section S1.3.1.

**Table 1:** The Time Series Models Were Calibrated for 1978 to 2005 and Validated for 2005 to 2020

	Calibration		Validation		Entire Period	
Aquifers	RMSE	Range	RMSE	Range	RMSE	Range
BK	1.7	4	1.5	8	1.6	11
PD	2.3	6	1.3	9	2.0	15
NL	3.9	13	1.9	14	3.2	25
NB	3.8	13	2.6	13	3.5	25
All	3.2	10	2.0	12	2.8	21

Note: The average RMSE and observed average total ranges for the calibration, validation, and entire period are shown for each aquifer. All values are in meters.

**Figure 8:**
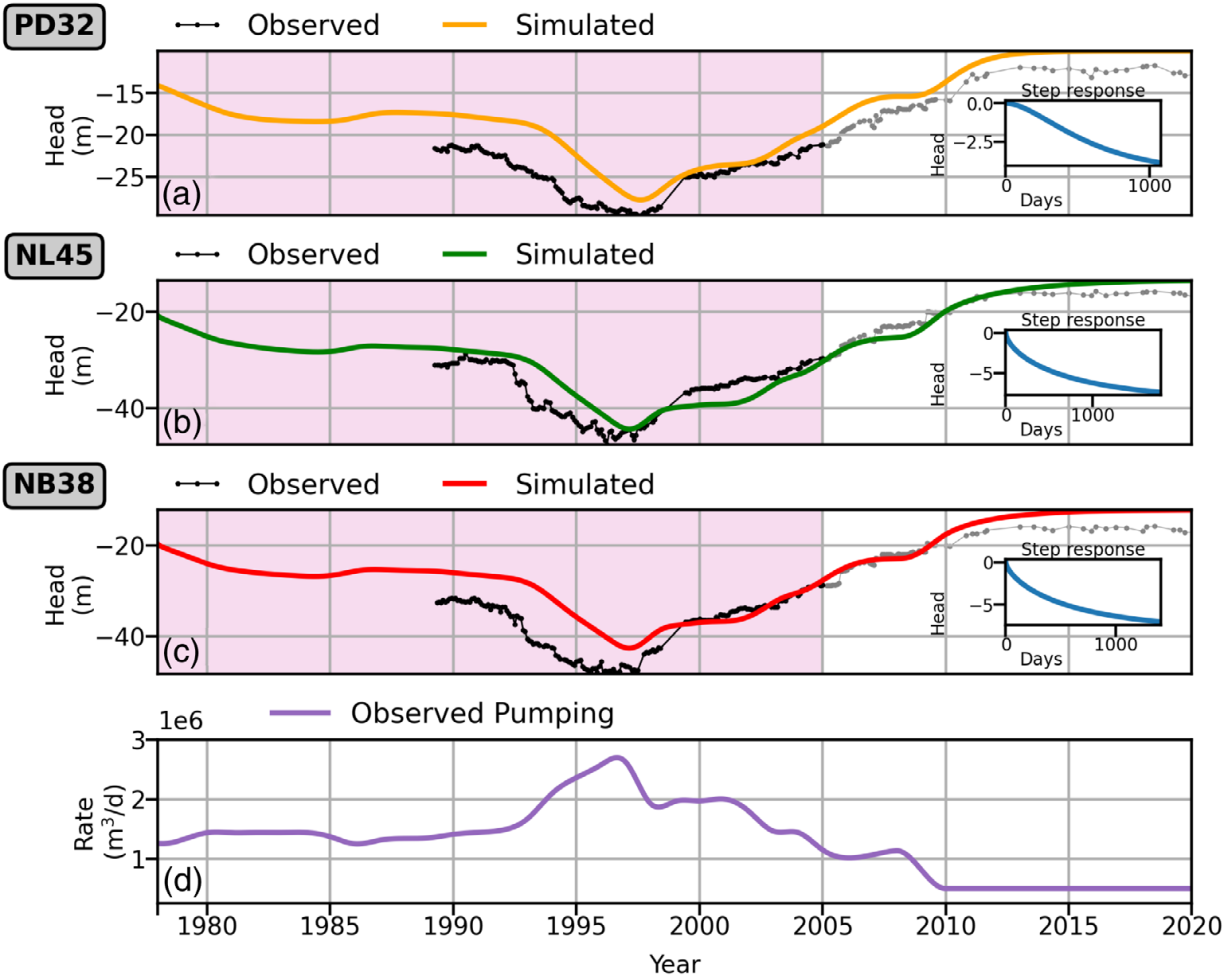
Results of time series models for wells in well nest BKK013 for 1978 to 2020. The pink box represents the calibration period (1978 to 2005). (a) Simulated heads (orange) and observations (black) for well PD32. (b) Simulated heads (green) and observations (black) for well NL45. (c) Simulated heads (red) and observations (black) for well NB38. (d) The basin‐wide pumping time series, which is the only input forcing for these models.

As an example, the model fit for well nest BKK013 is shown in Figure 8. The top three graphs contain simulated heads (orange, green, red) plotted against observed heads (black), while the inset graphs depict the step response, which is the head response due to a constant pumping rate of 500,000 m^3^/d. The input pumping time series (the forcing) is plotted in the bottom graph. The pink box highlights the calibration period (1978 to 2005). Similar plots for all other well nests are provided in Section S1.4. The RMSEs between simulated and observed head for wells PD32, NL45, and NB38 in well nest BKK013 for the entire time period are 17%, 11%, and 18% relative to their respective total observed ranges of head. The time series models simulate higher groundwater levels than observed pre‐1997 when compared to post‐1997 (as is the case for several other well nests, see Section S1.4). One of the likely reasons is the use of basin‐wide pumping rates instead of localized pumping rates, which are not available.

**Figure 9:**
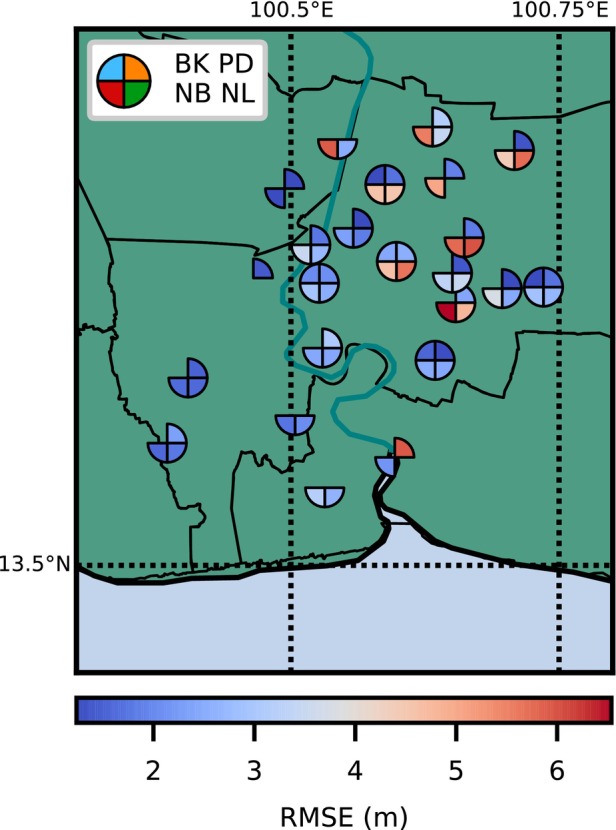
The RMSE of the head for each well in a specific aquifer at each well nest location.

The average RMSEs between the simulated and observed head for each aquifer for the entire time period are 13% to 15% of the associated observed data range (Table 1). The spatial distribution of RMSEs for all modeled wells is shown in Figure 9, with lower RMSEs shown in blue colors and higher RMSEs in red colors. At each well nest location, each wedge represents a well in a different aquifer and its RMSE. Missing wedges mean that measurements in respective aquifers were not available. The well nests that have higher RMSEs are localized to the eastern areas of the study site. A likely explanation for these higher RMSEs is that the basin‐wide pumping is not representative of localized pumping trends in this region.

**Figure 10:**
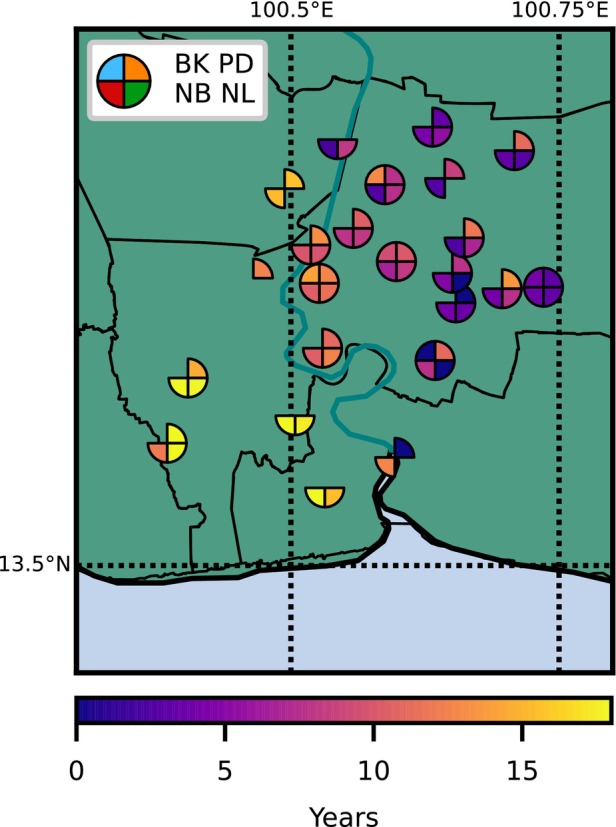
The response time $t_{90}$ for each well in a specific aquifer at each well nest location.

The groundwater response due to pumping is characterized by the response function of the pumping forcing. The response time is defined here by the time it takes for the system to reach 90% of the total response and is referred to as the $t_{90}$. The $t_{90}$ values of the time series models vary from 2 to 15 years, with some exceptions from poor model simulations. The spatial distribution of $t_{90}$ for the modeled wells is shown in Figure 10, with smaller response times in purple colors and larger response times in yellow colors. Most of the models with larger response times are located in well nests toward the southwestern part of the study area. Yet, they are not necessarily associated with higher RMSEs. In these cases, basin‐wide pumping may not be representative of localized pumping toward the southwestern regions.

### Subsidence

Land subsidence was modeled and calibrated at all 23 well nest locations. The average calibrated parameters of the clay and subsidence models for all well nests are reported in Table 2 and final calibrated ranges for all well nests are reported in Table S28.

**Table 2:** Average Calibrated Parameter Values

	$S_{v}$ [1/m] Average	$S_{e}$ [1/m] Average	$K_{v}$ [m/d] Average
BK clay	3e‐3	1e‐4	3e‐5
BK aquifer	—	1e‐5	—
PD clay	7e‐4	2e‐5	2e‐6
PD aquifer	—	2e‐6	—
NL clay	3e‐4	1e‐5	2e‐7
NL aquifer	—	1e‐6	—
NB clay	3e‐4	1e‐5	5e‐7
NB aquifer	—	1e‐6	—

**Figure 11:**
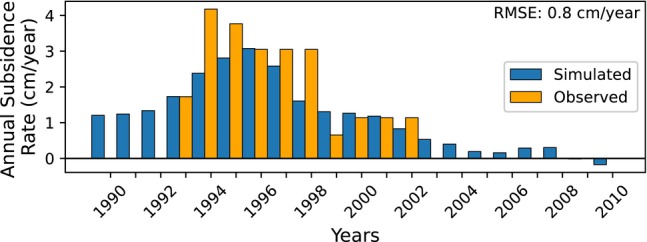
Simulated (blue) annual rates of land subsidence at well nest BKK013 compared to benchmark leveling (orange) at benchmark leveling station 5503. These benchmark levels were measured for 1993 to 2002.

An example of modeled pumping‐induced subsidence for well nest BKK013 is presented in Figure 11, where both the annual simulated subsidence rates (blue) and the annual observed subsidence rates from benchmark leveling measurements (orange) are plotted. For 1996 to 1998, the subsidence rate represents the average over 1996 to 1998 because benchmark levels were not measured for 1996 and 1997. Results for all other well nests are provided in Section S1.5. For well nest BKK013, annual rates of simulated land subsidence compare well to benchmark observations with a RMSE of 0.8 cm/year. The observed total range of subsidence rates for well nest BKK013 is 3.5 cm/year. Simulated land subsidence matches observed subsidence well for 2000 to 2002, but simulated rates are lower than observed for 1993 to 1995 and 1997 to 1998.

**Figure 12:**
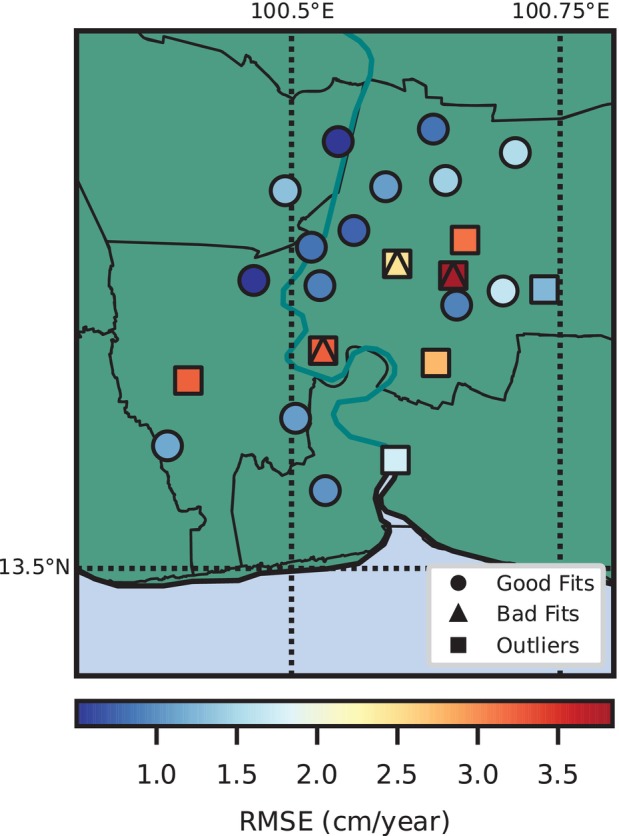
The RMSE of yearly land subsidence rates for each well nest. Models with poor fits and outliers in observations are characterized separately with triangle and square markers, respectively. [Correction added after first online publication on 09 December 2024. The figure 12 has been replaced with a new version.]

The average RMSE between simulated and observed subsidence for all well nests is 1.6 cm/year, compared to an observed average total range of 5.4 cm/year. The spatial distribution of the RMSEs for every well nest is shown in Figure 12, with blue colors representing lower RMSEs. Eight out of 23 models are characterized with outliers in subsidence observations and are indicated with a square in Figure 12. For three out of these eight well nests, outliers also resulted in a poor fit. A poor fit is defined as a model having either high RMSE or not capturing the overall trend in observed subsidence rates when visually examined (Barthel et al. 2022).

### Model Diagnostics

For an additional time series model check, heads in the aquifers simulated with the data‐driven time series models were compared to the results of a physics‐based MODFLOW model of the region, which used inputs that are independent from the time series models (Mikita et al. 2011). The present study found that 60 out of 66 time series models had response times between 5 and 20 years (Figure 10). As a comparison, the MODFLOW model was used to simulate pumping in the NL aquifer for 20 years at a constant rate. The MODFLOW model's response times were of the same order (5 to 20 years) as the response times of the time series models.

Comparing the compaction of each clay layer to the total land subsidence in the present study, BK Clay contributed on average 51%, PD Clay 29%, NL Clay 8%, and NB Clay 12%. These results roughly agree with the findings of Nutalaya et al. (1986), who found that the BK Clay contributes 30% to 50% to land subsidence at selected locations. Head data was available for the BK aquifer at only five locations. At these five locations, BK Clay contributed on average 50% and PD Clay 31%. At the other locations, the PD model was used as a proxy for BK, and BK Clay contributed on average 51% and PD Clay 28% to subsidence. The similar percentages between the proxy and independent model cases demonstrate that the PD models represent the BK layers well.

Simulated heads are higher than observed at early times at some well nests. The sensitivity of subsidence to the simulated groundwater levels in the aquifers at early times is analyzed for well nest BKK013 by increasing the pumping rate by 15% for 1954 to 1993 (in essence, it is hypothesized that localized pumping is 15% higher than basin‐wide pumping during 1954 to 1993). New time series models are fitted with the modified pumping rates; for models at wells PD32, NL45, and NB38, the RMSEs for the entire time period decrease 22%, 21%, and 25% using the modified rates, respectively (Figure S72). Subsidence is simulated using these new heads in the aquifers. Annual subsidence rates slightly increase compared to subsidence rates simulated without modifying the pumping time series, leading to an increase of cumulative subsidence of 3 cm, or 12%, in 1993 (Figure S74). It is concluded that the simulated subsidence is mildly sensitive to the simulated heads at early times. This analysis accentuates the importance of localized pumping, which unfortunately is not available.

Sensitivity analyses are also conducted for the subsidence models of well nest BKK013 using the four sets of calibration parameters ($S_{e}$ for aquifer layers, $S_{e}$ for clay layers, $S_{v}$ for clay layers, $K_{v}$ for clay layers). The sensitivity of initial layer thicknesses is also examined, since errors can be made in the interpretation of geologic logs. At well nest BKK013, the parameters associated with the subsidence model for each layer are adjusted between 50% and 150% of the calibrated values in 10% increments. The resulting cumulative land subsidence (cm) at well nest BKK013 for an extended period of 1978 to 2060 is computed for each set of parameters (Section S1.6.2). During periods of minimal subsidence, the model is sensitive to changes in the elastic specific storage term of the clay and sand layers. Simulated subsidence is affected by changes in initial layer thicknesses only as layers were made thinner. Delayed subsidence is small for thin layers, specifically clay layers, resulting in smaller overall cumulative subsidence. The subsidence model is especially sensitive to the value of the vertical hydraulic conductivity and inelastic specific storage, producing a difference of 16 and 36 cm of cumulative subsidence, respectively, that is, 34% and 75% of the total cumulative subsidence.

### Forecasts

**Figure 13:**
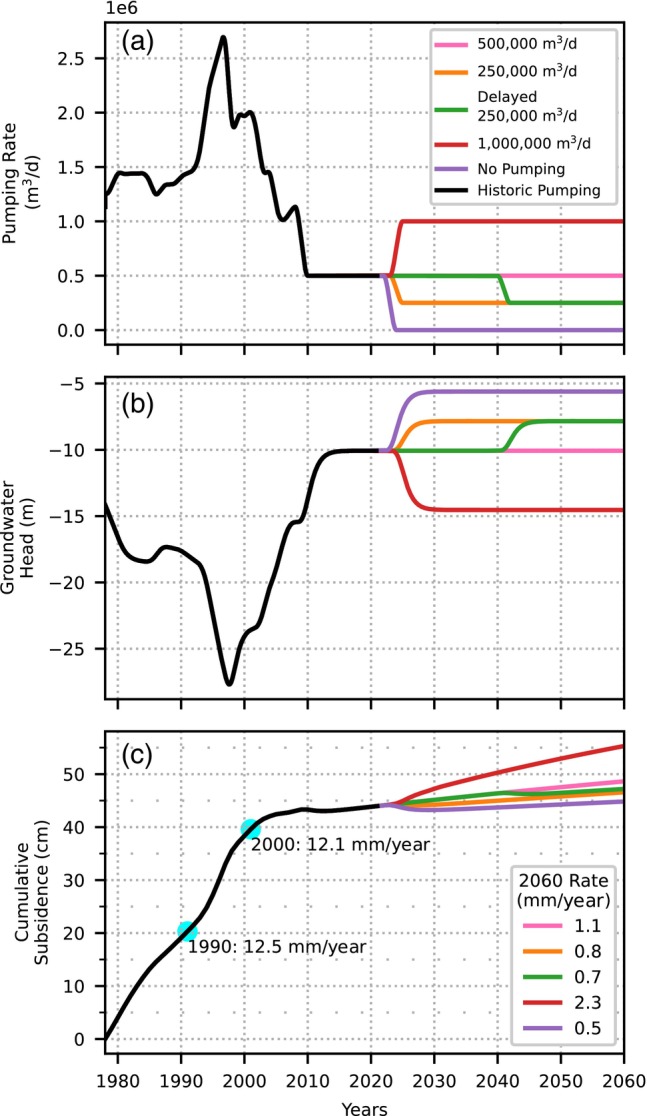
(a) Four pumping scenarios are used to forecast groundwater levels in the four aquifers; no pumping is used as a control. (b) Forecast of heads for four pumping scenarios for well PD32 in well nest BKK013 using the time series model. (c) Subsidence forecasts for well nest BKK013 vary from 0.5 to 2.3 mm/year by 2060 depending on the pumping scenario (subsidence with respect to 1978). Numbers on the lower right‐hand corner represent the annual rate of subsidence in 2060 in mm/year.

To evaluate the consequences of different pumping strategies on ground motion, subsidence is forecasted for four pumping scenarios starting in 2022: (1) a steady pumping rate of 500,000 m^3^/d for the next 40 years, (2) decreased pumping of 250,000 m^3^/d for the next 40 years, (3) steady pumping rate for the next 20 years with decreased pumping for the 20 years after, and (4) an increased pumping rate of 1,000,000 m^3^/d for the next 40 years (Figure 13a). Turning off pumping is considered unrealistic but is used as a control scenario nonetheless.

Time series models are used to forecast heads in the aquifers until 2060 using the four pumping scenarios. When pumping rates double, groundwater heads decline, and when pumping decreases, groundwater heads rise, as expected. An example is shown for the simulated heads in well PD32 of well nest BKK013 in Figure 13b. The control case of turning pumping off is used as a check to ensure that the model works properly. When pumping ceases, heads return to the value of constant d, which represents the time series model's estimate of the head prior to pumping.

Forecasts of subsidence until 2060 are generated with linked groundwater‐subsidence models at the 23 well nest locations for the different pumping scenarios. An example for well nest BKK013 is presented in Figure 13c, showing cumulative subsidence (cm) from 1978 until 2060. The annual subsidence rates in 2060 for the different scenarios are shown in the lower right‐hand corner of Figure 13c. Similar plots for all other well nests are provided in Section S1.5. For well nest BKK013, the annual subsidence rate in 2060 is highest at 2.3 mm/year for a pumping rate of 1,000,000 m^3^/d compared to 12 to 13 mm/year in 1990 and 2000. Under all pumping scenarios, subsidence is still expected to occur in 2060. Rates will not drop below 1 mm/year beyond year 2100 for the pumping scenario of 1,000,000 m^3^/d, while the 500,000, 250,000, delayed 250,000^3^/d, and no pumping scenarios result in rates below 1 mm/year in the years 2078, 2023, 2040, and 2022, respectively.

The average subsidence rates are forecasted to be around 1.2 mm/year with a maximum of 5 mm/year for Bangkok in 2060, for the basin‐wide pumping scenario of 1,000,000 m^3^/d. In the nearby Mekong Delta in Vietnam, Minderhoud et al. (2020) utilized a three‐dimensional physics‐based groundwater model coupled to a subsidence module to forecast subsidence until 2100 using several pumping scenarios. A basin‐wide pumping rate of 1,000,000 m^3^/d resulted in subsidence rates ranging 1.0 to 2.7 mm/year in 2050. These subsidence rates in response to the same basin‐wide pumping rate are of the same order as for the Bangkok area.

**Figure 14:**
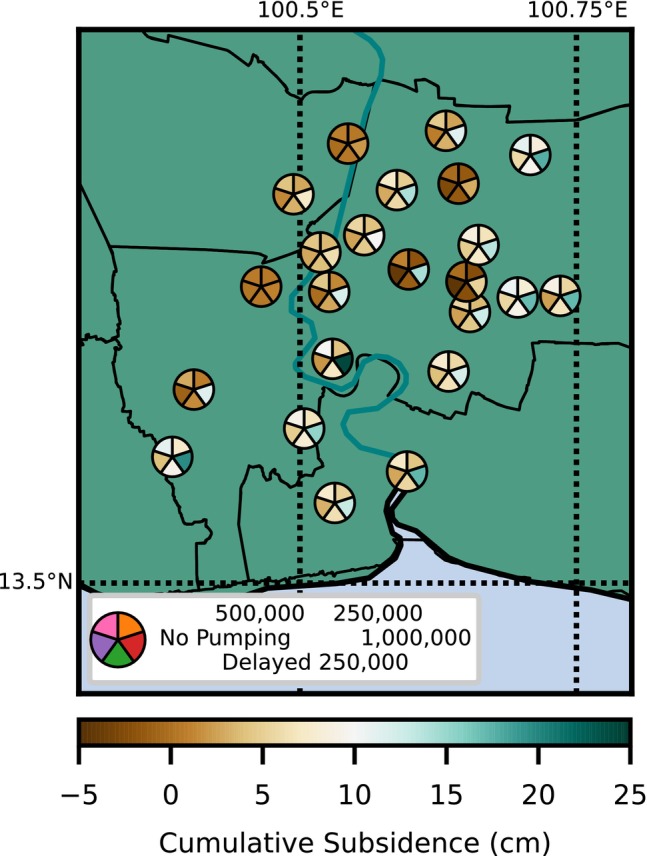
Cumulative subsidence (cm) from 2020 to 2060 for the pumping scenarios for each well nest. Each wedge represents cumulative subsidence for a particular pumping scenario.

The forecasted cumulative subsidence (2020 to 2060) for all well nests and all five scenarios (including the control scenario) is shown in Figure 14. Larger cumulative subsidence is shown in green colors and smaller cumulative subsidence in brown colors. An additional 5 cm of subsidence is expected for the period 2020 to 2060 for most well nests and pumping scenarios (Figure 14). Under the most intense pumping scenario of 1,000,000 m^3^/d, an additional 10 to 25 cm of subsidence (2020 to 2060) is forecasted for some well nests.

## Discussion

Groundwater and subsidence are typically simulated using spatially distributed physics‐based models. However, large amounts of hydrogeological data and observations are required for development and calibration of these models, which prove a difficult limitation to overcome. This paper presents a parsimonious method that combines data‐driven and one‐dimensional physics‐based models to simulate and forecast heads and subsidence with minimal data and parameters in deltaic systems. Data‐driven models work with fewer parameters than physics‐based models but require time series of observations for at least part of the simulation period. The combination of a data‐driven model for the heads in the aquifers and physics‐based models for the subsidence results in an approach with relatively few parameters that can deal with sparse data while providing forecasting capabilities.

The developed approach was applied to 23 well nests in Bangkok and gave reasonable results for groundwater at every well nest and subsidence at 15 well nests. The fit between measured and modeled groundwater and subsidence were on the same order as those of previous works (Nutalaya et al. 1986; Mikita et al. 2011; Minderhoud et al. 2020). Comparing the results of this parsimonious, linked approach with those that utilized different methods, inputs, and many more parameters showed that a parsimonious approach with only eight parameters can give comparable results at a well nest.

Time series models for groundwater modeling in the aquifers of Bangkok were able to simulate head with good fits to observations both for the calibration and validation periods. Comparing the calibration and validation results, the RMSEs decreased for validation. The validation period is characterized by a smaller pumping signal and recovery of heads. The models were able to simulate groundwater recovery very well. Overall, the average RMSEs of each aquifer are in the range of 13% to 15% of their associated observed data range, indicating that the models were successfully capturing groundwater dynamics with estimated basin‐wide pumping time series as a driver.

In the present application, RMSE results suggest that the subsidence models perform reasonably well at 15 of 23 locations. The models were able to capture the rise in subsidence rates for 1990 to 1995 and decline for 1995‐early 2000s. The average RMSE is about 30% of the data range. This means the method performs reasonably well, especially since pumping‐induced subsidence represents roughly 70% of total subsidence in Bangkok (Chulalongkorn University Faculty of Engineering 2009). A poor fit was obtained at three locations, all of which had outliers and made a good fit impossible. Sudden uplifts measured in the observations occur in different years for different well nests and do not coincide with groundwater variations. These may be due to measurement error or unknown local processes. The 13 to 14 cm/year spikes in 2005 to 2006 observed at four well nests are most likely due to measurement error as they all occur in the same year and the values are an order of magnitude larger than the observed values before and after the spike. There are three wells with subsidence measurements prior to 1988; two of which gave poor fits with RMSEs of 2.5 and 3.8 cm/year. The poor fit is most likely due to the averaging of rates for periods with missing measurements or regional pumping rates that differ from the basin‐wide pumping rates. In other studies using time series analysis, it is common that measured data cannot be simulated well at a number of observation points because of missing forcings or differences in local conditions (e.g., Brakenhoff et al. 2022). In addition, proxies are used for aquifers in this study that were not measured but may have significant pumping, but the use of proxies in some aquifers did not necessarily coincide with poor subsidence fits.

Previously, land subsidence was simulated at specific sites in Bangkok by, for example, Intui et al. (2022), Saowiang and Giao (2020), and Giao et al. (2013). These authors assumed that land subsidence was due to compaction of the upper 80 m of the subsurface, with an eventual complete groundwater recovery. Saowiang and Giao (2020) and Giao et al. (2013) found rebound occurring during 1960 to 2016 and 1957 to 2040, respectively. Ishitsuka et al. (2014) also found uplift of 0.5 to 3.0 cm occurring during 2007 to 2010 after which subsidence stabilized when analyzing satellite data. A small uplift of 0.5 to 10 cm is simulated at five well nests in this study for the no‐pumping scenario for 2020 to 2060 (e.g., BKK005 in Section S1.5), while no uplift is simulated for the other well nests. At six out of seven locations where subsidence stabilizes, the calibrated vertical hydraulic conductivity values of the clay layers were higher. As a result, groundwater in the clay layers equilibrates quickly with groundwater in the aquifers, and compaction of the clay layers stabilizes as groundwater levels in the aquifers stabilize.

Simulated subsidence matches the sparse subsidence measurements reasonably well at 15 locations, especially considering the uncertainties of the subsidence observations and the lack of local pumping data. A better subsidence simulation can possibly be achieved by incorporating other deformation processes, such as viscous creep (Kooi and Erkens 2020) and lateral deformation (Moh and Woo 1987), which were neglected in this study. It is noted that the use of a basin‐wide, uniform pumping time series may result in site‐to‐site variability in the calibrated specific storage and hydraulic conductivity parameters. The calibrated inelastic and elastic specific storage values both vary two orders of magnitude over the study area, while the calibrated vertical hydraulic conductivity varies three orders of magnitude over the study area (Table S28). Although the spatial variability in calibrated parameters may indeed compensate for the lack of spatial variability in pumping, the final ranges in the calibrated parameters are considered reasonable when compared to other studies (Chowdhury et al. 2022; van Leer et al. 2023).

Future work will focus on obtaining a more physics‐based estimate using data assimilation. Data assimilation is an approach that combines observations with model outputs (which is considered prior knowledge) in an attempt to estimate the likelihood of subsidence and parameter values based on the model, data, and their respective uncertainties (e.g., Evensen et al. 2022). Utilizing additional data from satellites and GPS with data assimilation may also help to improve subsidence calibration and forecasts (e.g., Gazzola et al. 2021).

## Conclusion

A parsimonious, linked data‐driven and physics‐based approach was developed to simulate pumping‐induced land subsidence at well nests. The approach is applicable to deltas with layered aquifers consisting of a sequence of sand and clay layers. One‐dimensional groundwater models are linked to subsidence models to examine subsidence under current and future groundwater conditions. Measurements of heads in aquifers and total subsidence rates must be available for at least a portion of the simulation period, while measurements (or estimates) of pumping must be available for the entire period. Time series models of the groundwater head required calibration of four parameters for each aquifer where heads were measured. The subsidence model used the simulated heads as input and required calibration of three parameters for each well nest. The approach was applied to observation well nests in Bangkok, Thailand. Results were used to forecast subsidence using various pumping scenarios until 2060.

The linked models located at well nests near the center of the study area performed reasonably well when compared to groundwater and subsidence observations, with calibrated parameters consistent with previous research. Subsidence is expected to persist in the Bangkok area at a rate of a few millimeters per year until at least 2060. Subsidence remains highly susceptible to changes in groundwater pumping, underscoring the need for a linked groundwater‐subsidence model for analyzing subsidence. Overall, the developed method is a promising approach to analyze pumping‐induced land subsidence in data‐scarce deltaic areas. The example application to Bangkok results in forecasts that may be useful for water policy decision makers.

## Authors' Note

The authors do not have any conflicts of interest or financial disclosures to report.

## Supporting information


**Data S1.** Statistics on observational data, modeling notes, and calibration results of groundwater and subsidence for other well nests.

## Data Availability

The data and models presented in this paper are available on github: https://github.com/jsoontho/BKKSubPastasModels.
