# Supplementary material for: Linked Data‐Driven, Physics‐Based Modeling of Pumping‐Induced Subsidence with Application to Bangkok, Thailand
Source: Ground Water. 2024 Oct 11;63(2):145–59. doi: 10.1111/gwat.13443 (PMC11875053; doi:10.1111/gwat.13443)
Supplement: Supplementary file 1 — Data S1. Statistics on observational data, modeling notes, and calibration results of groundwater and subsidence for other well nests. [file GWAT-63-145-s001.pdf]

Supporting Information

Additional information is provided for the paper submitted to *Groundwater* titled, "Linked data-driven, physics-based modeling of pumping-induced subsidence with application to Bangkok, Thailand" The authors are Jenny T. Soonthornrangsan, Mark Bakker, and Femke C. Vossepoel. The abstract is provided below:

Research into land subsidence caused by groundwater withdrawal is hindered by the availability of measured heads, subsidence, and forcings. In this paper, a parsimonious, linked data-driven and physics-based approach is introduced to simulate pumping-induced subsidence; the approach is intended to be applied at observation well nests. Time series analysis using response functions is applied to simulate heads in aquifers. The heads in the clay layers are simulated with a one-dimensional diffusion model, using the heads in the aquifers as boundary conditions. Finally, simulated heads in the layers are used to model land subsidence. The developed approach is applied to the city of Bangkok, Thailand, where relatively short time series of head and subsidence measurements are available at or near 23 well nests; an estimate of basin-wide pumping is available for a longer period. Despite the data scarcity, data-driven time series models at observation wells successfully simulate groundwater dynamics in aquifers with an average root mean square error (RMSE) of 2.8 m, relative to an average total range of 21 m. Simulated subsidence matches sparse (and sometimes very noisy) land subsidence measurements reasonably well with an average RMSE of 1.6 cm/year, relative to an average total range of 5.4 cm/year. Performance is not good at eight out of 23 lo-

cations, most likely because basin-wide pumping is not representative of localized pumping. Overall, this study demonstrates the potential of a parsimonious, linked data-driven, and physics-based approach to model pumping-induced subsidence in areas with limited data.

1.1 Observations

1.1.1 Groundwater

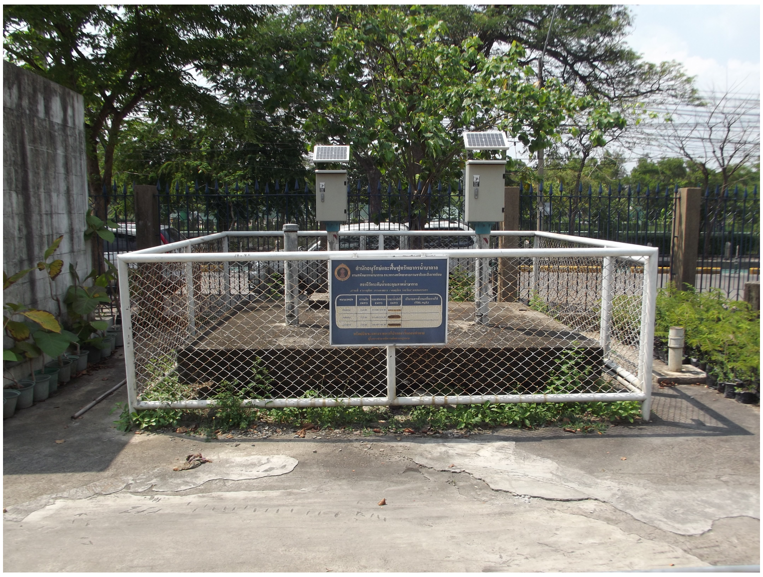

Figure 1.1: A picture of a typical groundwater well nest in Bangkok, Thailand (provided by email communication from Dr. Anuphao Aobpaet).

Table 1.1: Observed groundwater head statistics for each available well in each well nest.

Well Nest	Well	Average (m)	Standard Deviation (m)	Min (m)	Max (m)	Range (m)
LCBKK003	BK2	-15.14	4.10	-20.75	-8.17	12.59
	PD17	-18.21	2.78	-21.88	-10.93	10.95
	NL34	-21.31	3.05	-26.39	-13.52	12.87
	NB27	-22.14	5.81	-31.49	-13.75	17.74
LCBKK005	PD21	-16.57	5.36	-24.10	-8.63	15.46
	NL36	-30.20	12.26	-49.39	-14.31	35.08
	NB28	-28.69	10.59	-46.44	-7.82	38.62
LCBKK006	BK_JS	-15.67	4.99	-22.68	-8.57	14.11

	PD20	-16.64	4.99	-23.65	-9.54	14.11
	NL35	-26.83	9.42	-40.66	-13.61	27.05
	NB23	-25.86	8.21	-38.80	-14.10	24.70
LCBKK007	PD25	-16.38	4.06	-22.01	-10.40	11.61
	NL39	-21.50	5.63	-30.27	-13.76	16.51
	NB32	-23.37	6.37	-33.96	-14.84	19.12
LCBKK009	PD26	-16.03	4.28	-22.97	-9.88	13.09
	NB36	-29.16	10.24	-49.40	-16.87	32.53
LCBKK011	BK3	-15.04	4.41	-21.71	-8.45	13.26
	PD27	-17.72	5.25	-24.83	-10.41	14.42
	NL42	-26.87	9.68	-44.39	-14.41	29.98
	NB35	-25.68	7.99	-40.22	-15.45	24.77
LCBKK012	PD23	-19.58	5.35	-26.64	-11.85	14.79
	NL38	-20.79	5.20	-28.42	-13.30	15.12
	NB33	-23.11	5.63	-32.33	-15.27	17.06
LCBKK013	PD32	-19.51	5.92	-29.49	-11.78	17.71
	NL45	-27.73	9.92	-47.50	-15.81	31.69
	NB38	-28.16	10.76	-48.22	-15.74	32.48
LCBKK014	PD84	-15.40	4.19	-21.88	-9.95	11.93
	NL54	-34.53	12.29	-56.43	-19.04	37.39
	NB47	-31.10	10.59	-51.28	-18.31	32.97
LCBKK015	PD34	-16.73	4.80	-23.58	-9.32	14.26
	NL48	-23.67	7.59	-35.26	-3.04	32.22
	NB42	-24.17	7.05	-35.68	-14.74	20.94
LCBKK016	NL58	-20.77	5.16	-28.40	-13.62	14.78
	NB54	-22.72	4.92	-30.33	-15.81	14.52
LCBKK018	PD82	-16.36	4.07	-22.53	-10.14	12.39
LCBKK020	PD41	-15.60	4.16	-21.64	-9.64	12.00
	NL56	-32.13	12.37	-52.49	-16.73	35.76
	NB51	-32.12	12.25	-54.37	-17.49	36.88
LCBKK021	PD36	-15.67	4.04	-21.47	-9.72	11.75
	NL52	-35.30	14.34	-58.86	-17.38	41.48
	NB45	-32.62	12.08	-54.59	-17.92	36.67
LCBKK026	PD62	-17.21	3.97	-22.39	-9.78	12.61
	NL18	-20.76	3.96	-27.27	-14.50	12.77

	NB66	-25.22	5.20	-33.17	-18.38	14.79

LCBKK027	PD57	-16.99	4.19	-22.48	-10.04	12.44
	NL70	-19.46	4.49	-25.61	-12.95	12.66
	NB62	-24.22	4.78	-31.10	-17.45	13.65
LCBKK036	BK_JICA8	-11.26	2.62	-15.95	-7.55	8.40
	PD_JICA7	-27.52	12.71	-53.16	-12.85	40.31
	NL_JICA6	-34.03	16.14	-65.75	-17.73	48.02
	NB_JICA5	-32.69	14.09	-59.54	-17.63	41.91
LCBKK038	BK1	-12.29	3.33	-17.22	-8.30	8.92
	PD26_009	-16.81	4.16	-23.31	-10.22	13.09
	NL31	-25.80	8.60	-39.05	-13.58	25.47
	NB36_009	-29.66	10.21	-49.74	-17.25	32.49
LCBKK041	PD138	-18.57	4.43	-25.36	-11.14	14.22
	NL140	-35.16	11.99	-51.69	-16.58	35.11
	NB139	-34.44	11.25	-51.12	-17.10	34.02
LCNBI003	NL41	-19.25	5.12	-27.88	-12.08	15.80
	NB24	-22.80	6.00	-33.67	-14.81	18.86
LCNBI007	PD56	-14.35	2.97	-18.73	-10.17	8.56
	NB63	-20.78	4.21	-27.98	-15.20	12.78
LCSPK007	PD22	-19.58	7.43	-32.42	-9.70	22.72
	NB30	-23.91	5.91	-34.15	-14.93	19.22
LCSPK009	NL67	-23.09	3.59	-27.87	-14.39	13.48
	NB53	-23.15	3.54	-28.38	-14.20	14.18
All		-22.92	9.95	-65.75	-3.04	62.71

1.1.2 Subsidence

Table 1.2: Observed subsidence statistics for benchmark leveling stations near each well nest.

Well Nest	Leveling Station	Average (cm/yr)	Standard Deviation (cm/yr)	Min (cm/yr)	Max (cm/yr)	Range (cm/yr)
LCBKK003	DMR62	1.55	1.02	0.51	3.52	3.01
LCBKK005	Cl18-1	3.11	3.50	-0.72	13.74	14.46
LCBKK006	Cl05-1	3.57	2.46	0.77	7.76	6.99
LCBKK007	DMR63	0.71	0.76	-0.01	2.31	2.32

LCBKK009	BMS8313/1	2.89	1.17	1.84	5.22	3.38
LCBKK011	CI14-3/DMR49	1.26	2.64	-2.02	13.54	15.55
LCBKK012	DMR84	0.85	1.54	-1.16	5.31	6.47
LCBKK013	5503	2.86	1.07	0.65	4.18	3.52
LCBKK014	DMR29	2.10	1.60	-0.50	5.58	6.08
LCBKK015	DMR61	1.69	0.71	1.13	3.55	2.42
LCBKK016	Cl22-1	1.55	1.07	-0.47	3.53	4.00
LCBKK018	DMR79	0.31	0.33	-0.53	0.74	1.26
LCBKK020	DMR27	2.07	2.89	-0.41	13.17	13.58
LCBKK021	DMR66	2.92	1.19	0.50	4.82	4.32
LCBKK026	DMR07	2.79	0.74	1.64	3.64	2.00
LCBKK027	DMR09	2.89	2.82	0.64	14.23	13.59
LCBKK036	BMA_SiteA	2.89	1.16	1.92	5.61	3.69
LCBKK038	DMR69	2.74	0.59	2.10	3.66	1.56
LCBKK041	4608	2.95	1.28	1.87	5.00	3.13
LCNBI003	DMR17	1.02	0.60	-0.23	1.94	2.17
LCNBI007	DMR16	1.18	1.08	0.16	2.82	2.66
LCSPK007	1703	3.21	1.55	1.79	7.08	5.28
LCSPK009	DMR12	2.13	0.76	0.81	3.37	2.56
All		2.13	2.20	-2.02	14.23	16.25

1.2 Groundwater in the Clay Layer Modeling

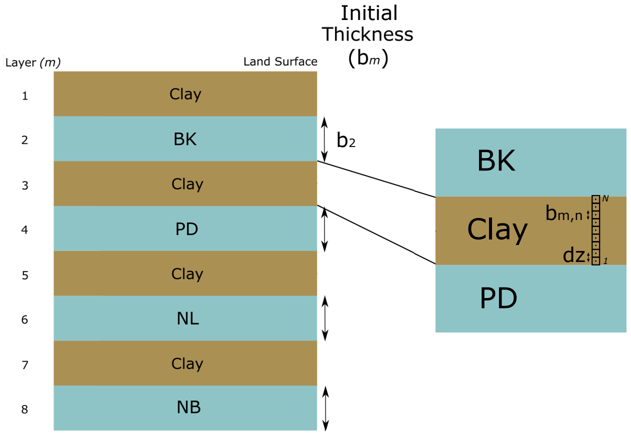

Figure 1.2: a) Each Bangkok confined aquifer is overlaid by a clay layer. Model layers in the clay layers are affected by heads from the top aquifer and heads from the aquifer beneath. b) Within each clay layer, model layers are created for the finite difference approximation.

A finite-difference numerical modeling approach is followed to simulate the groundwater heads in clay layers based on simulated heads in four aquifers (Figure 1.2). Specifically, an implicit finite-difference approximation is utilized to solve a 1D diffusion equation describing the lagged head changes in the clay layers (Equation 1.1).

$$\frac{\partial^2 h}{\partial z^2} = \frac{S}{K_v}\frac{\partial h}{\partial t} \qquad S\begin{cases} S_e & \text{for } h > h_{min} \\ S_v & \text{for } h \leq h_{min} \end{cases} \tag{1.1}$$

where S is the skeletal specific storage (S_e: elastic or S_v: inelastic value depending on whether the preconsolidation head was exceeded) [1/L], K_v is the vertical hydraulic conductivity of clay [L/T], z is the vertical coordinate [L], and t is time [T].

This scheme was originally introduced in a MODFLOW groundwater model package (Hoffmann et al. 2003), but in this study, it is linked to Pastas time series models instead of linked to MODFLOW. The approximation is represented by a system of equations in matrix form and solved using an inverse matrix (Equations 1.2 - 1.11).

$$[A]^m [h]^m = [r]^m \tag{1.2}$$

where $[A]^m$ is an N by N symmetric, tridiagonal matrix.

$$A_{ij}^m = \frac{K_v}{\Delta z} \quad \text{for off-diagonal elements } i \neq j \tag{1.3}$$

$$A_{ij}^m = -2\frac{K_v}{\Delta z} - S_i^m \frac{\Delta z}{\Delta t} \quad \text{for } 1 < i < N \tag{1.4}$$

For all clay layers besides the top clay layer:

$$A_{11}^m = A_N^m = -3\frac{K_v}{\Delta z} - S_{1,N}^m \frac{\Delta z}{\Delta t} \tag{1.5}$$

For the top clay layer, where the Nth model layer is a no-flow boundary:

$$A_{11}^m = -3\frac{K_v}{\Delta z} - S_1^m \frac{\Delta z}{\Delta t} \tag{1.6}$$

$$A_N^m = -\frac{K_v}{\Delta z} - S_N^m \frac{\Delta z}{2\Delta t} \tag{1.7}$$

Note that equation 1.7 is different from equation 21d in Hoffmann et al. (2003). When equation 1.7 is derived, there is a negative before $\frac{K_v}{\Delta z}$, which is not reported in Hoffmann et al. (2003).

where $[h]^m$ is an N by 1 vector of head values in clay model layers.

where $[r]^m$ is an N by 1 vector of known quantities defined by the following equations.

$$r_i^m = \frac{\Delta z}{\Delta t}[-S_i^m H_i^{m-1} + S_{e_i}^m (H_i^{m-1} - h_i^{m-1})] \quad \text{for } 1 < i < N \tag{1.8}$$

For all clay layers besides the top clay layer:

$$r_1^m = r_N^m = \frac{\Delta z}{\Delta t}[-S^m H_{1,N}^{m-1} + S_e^m (H_{1,N}^{m-1} - h_{1,N}^{m-1})] - 2\frac{K_v}{\Delta z} h_j^m \tag{1.9}$$

For the top clay layer, where the Nth model layer is a no-flow boundary:

$$r_1^m = \frac{\Delta z}{\Delta t}[-S_1^m H_1^{m-1} + S_{e_1}^m (H_1^{m-1} - h_1^{m-1})] - 2\frac{K_v}{\Delta z} h_j^m \tag{1.10}$$

$$r_N^m = \frac{\Delta z}{2\Delta t}[-S_N^m H_N^{m-1} + S_{e_N}^m (H_N^{m-1} - h_N^{m-1})] \tag{1.11}$$

where K_v is the vertical hydraulic conductivity for the entire clay layer (assumed constant) [L/T], Δz, or dz in figure 1.2b, is the distance between two model layers (constant because the change in thickness is assumed to be small compared to its original thickness) [L], Δt is the length of the time step [T], S_i^m is the skeletal specific storage at model layer i and time-step m (can be elastic or inelastic) [1/L], h_j^m is the head in the aquifer model layer j at time-step m [L], H_i^{m-1} is the preconsolidation head (lowest previous head) at model layer i at time-step $m-1$ [L], and h_i^{m-1} is the head at model layer i at time-step $m-1$ [L].

Available observations of subsidence consist of benchmark leveling measurements from the Royal Thai Survey Department (Royal Thai Survey Department 2021). Benchmark measurements were collected irregularly from 1978 onwards, and thus, rates are averaged over

gap years between measurements. The closest benchmark observations to the well nests are used for calibration. All leveling observations used for calibration were taken at 1 m depth. It is assumed to be the closest representative of land subsidence.

Calibration of hydraulic conductivity and specific storage parameters are conducted manually. Japan International Cooperation Agency (1995) provided ranges for specific storage and hydraulic conductivity. Specific storage terms are calculated from compressibility values, which were obtained from consolidation tests. It is assumed that these values represent inelastic specific storage (S_v). This also gives ranges for elastic storage, since inelastic specific storage tends to be 20 to more than 100 times greater than elastic storage (Riley 1998). Horizontal hydraulic conductivity values were obtained from pumping tests. Isotropy and homogeneity are assumed. It is noted that benchmark measurements had occasional outliers.

1.3 Calibration

1.3.1 Groundwater in the Aquifer Modeling

Table 1.3: Parameters for the Pastas models for well nest LCBKK003.

Well Nest	Well	Parameters	Initial	Optimal
LCBKK003	BK2	A	-0.017	-0.07 ± 5.4%
		n	1	0.33 ± 27.7%
		a	10	2070.07 ± 49.9%
		d	-6.7	-6.7 ± 7.7%
	PD17	A	-0.017	-0.07 ± 1.6%
		n	1	0.30 ± 7.0%
		a	10	2561.8 ± 13.0%
		d	-7.1	-7.1 ± 2.2%
	NL34	A	-0.017	-0.09 ± 1.5%
		n	1	0.26 ± 6.7%
		a	10	3102.1 ± 13.0%
		d	-6.7	-6.7 ± 2.9%
	NB27	A	-0.017	-0.12 ± 5.5%
		n	1	0.25 ± 20.5%
		a	10	4071.0 ± 43.3%
		d	-5.97	-5.97 ± 14.0%

Table 1.4: Parameters for the Pastas models for well nest LCBKK005.

Well Nest	Well	Parameters	Initial	Optimal
LCBKK005	PD21	A	-0.017	-0.09 ± 3.4%
		n	1	0.20 ± 17.3%
		a	10	3523.4 ± 40.2%
		d	-5.4	-5.4 ± 6.5%
	NL36	A	-0.017	-0.2 ± 3.8%
		n	1	0.1 ± 27.0%
		a	10	4296.5 ± 73.6%
		d	-4.9	-4.9 ± 17.7%
	NB28	A	-0.017	-0.2 ± 5.3%
		n	1	0.14 ± 11.3%
		a	10	10000 ± 45.3%
		d	-4.0	-4.0 ± 23.4%

Table 1.5: Parameters for the Pastas models for well nest LCBKK006.

Well Nest	Well	Parameters	Initial	Optimal
LCBKK006	Proxy	A	-0.017	-0.08 ± 3.8%
		n	1	0.23 ± 22.7%
		a	10	2175.4 ± 44.7%
		d	-6.1	-6.1 ± 6.7%
	PD20	A	-0.017	-0.08 ± 3.9%
		n	1	0.24 ± 19.9%
		a	10	2513.7 ± 40.5%
		d	-6.2	-6.2 ± 7.2%
	NL35	A	-0.017	-0.2 ± 5.2%
		n	1	0.2 ± 28.4%
		a	10	3253 ± 65.5%
		d	-5.8	-5.8 ± 19.3%
	NB23	A	-0.017	-0.2 ± 6.9%
		n	1	0.2 ± 31.1%
		a	10	3417.3 ± 67.6%
		d	-5.0	-5.0 ± 28.3%

Table 1.6: Parameters for the Pastas models for well nest LCBKK007.

Well Nest	Well	Parameters	Initial	Optimal
LCBKK007	PD25	A	-0.017	-0.07 ± 3.0%
		n	1	0.3 ± 10.3%
		a	10	2377.0 ± 3.5%
		d	-7.6	-7.6 ± 5.3%
	NL39	A	-0.017	-0.1 ± 6.4%
		n	1	0.2 ± 22.6%
		a	10	4461.6 ± 54.4%
		d	-7.2	-7.2 ± 12.0%
	NB32	A	-0.017	-0.1 ± 8.0%
		n	1	0.2 ± 24.3%
		a	10	7620.0 ± 76.2%
		d	-6.4	-6.4 ± 16.5%

Table 1.7: Parameters for the Pastas models for well nest LCBKK009.

Well Nest	Well	Parameters	Initial	Optimal
LCBKK009	PD26	A	-0.017	-0.08 ± 6.4%
		n	1	0.2 ± 20.0%
		a	10	4407.2 ± 48.0%
		d	-5.2	-5.2 ± 12.7%
	NB36	A	-0.017	-0.2 ± 7.0%
		n	1	0.1 ± 20.4%
		a	10	10000 ± 82.9%
		d	-3.8	-3.8 ± 32.8%

Table 1.8: Parameters for the Pastas models for well nest LCBKK011.

Well Nest	Well	Parameters	Initial	Optimal
	BK3	A	-0.017	-0.08 ± 3.3%
		n	1	0.35 ± 22.3%
		a	10	2242.6 ± 39.7%
		d	-4.8	-4.8 ± 6.6%
	PD27	A	-0.017	-0.09 ± 6.2%
		n	1	0.2 ± 12.0%
		a	10	8394.0 ± 46.5%

LCBKK011

		d	-5.8	-5.8 ± 9.6%
	NL42	A	-0.017	-0.2 ± 4.5%
		n	1	0.1 ± 11.0%
		a	10	10000 ± 52.6%
		d	-5.3	-5.3 ± 13.1%
	NB35	A	-0.017	-0.2 ± 4.5%
		n	1	0.2 ± 6.5%
		a	10	10000 ± 25.0%
		d	-4.6	-4.6 ± 14.7%

Table 1.9: Parameters for the Pastas models for well nest LCBKK012.

Well Nest	Well	Parameters	Initial	Optimal
LCBKK012	PD23	A	-0.017	-0.09 ± 7.7%
		n	1	0.3 ± 33.2%
		a	10	3126.4 ± 70.2%
		d	-7.5	-7.5 ± 12.8%
	NL38	A	-0.017	-0.1 ± 5.6%
		n	1	0.3 ± 16.3%
		a	10	3944.5 ± 36.0%
		d	-7.2	-7.2 ± 10.0%
	NB33	A	-0.017	-0.1 ± 5.6%
		n	1	0.3 ± 13.0%
		a	10	6228.1 ± 34.2%
		d	-6.5	-6.5 ± 12.0%

Table 1.10: Parameters for the Pastas models for well nest LCBKK013.

Well Nest	Well	Parameters	Initial	Optimal
LCBKK013	PD32	A	-0.017	-0.1 ± 5.5%
		n	1	0.2 ± 18.9%
		a	10	4643.2 ± 50.6%
		d	-5.6	-5.6 ± 12.9%
	NL45	A	-0.017	-0.2 ± 6.9%
		n	1	0.09 ± 23.6%
		a	10	10000 ± 98.6%

		d	-5.0	-5.0 ± 22.3%
	NB38	A	-0.017	-0.2 ± 8.0%
		n	1	0.06 ± 41.7%
		a	10	10000 ± 184.5%
		d	-4.1	-4.1 ± 33.8%

Table 1.11: Parameters for the Pastas models for well nest LCBKK014.

Well Nest	Well	Parameters	Initial	Optimal
LCBKK014	PD84	A	-0.017	-0.07 ± 3.6%
		n	1	0.3 ± 8.4%
		a	10	3672.1 ± 22.4%
		d	-6.2	-6.2 ± 5.5%
	NL54	A	-0.017	-0.2 ± 4.0%
		n	1	0.3 ± 15.8%
		a	10	2774.5 ± 33.7%
		d	-5.5	-5.5 ± 22.3%
	NB47	A	-0.017	-0.2 ± 5.0%
		n	1	0.2 ± 15.0%
		a	10	5307.8 ± 43.3%
		d	-4.7	-4.7 ± 23.4%

Table 1.12: Parameters for the Pastas models for well nest LCBKK015.

Well Nest	Well	Parameters	Initial	Optimal
LCBKK015	PD34	A	-0.017	-0.08 ± 2.2%
		n	1	0.2 ± 12.9%
		a	10	3030.2 ± 27.8%
		d	-6.8	-6.8 ± 3.0%
	NL48	A	-0.017	-0.1 ± 5.3%
		n	1	0.1 ± 9.5%
		a	10	10000 ± 41.1%
		d	-6.4	-6.4 ± 10.2%
	NB42	A	-0.017	-0.2 ± 4.7%
		n	1	0.2 ± 6.8%
		a	10	10000 ± 25.6%

		d	-5.6	-5.6 ± 10.9%

Table 1.13: Parameters for the Pastas models for well nest LCBKK016.

Well Nest	Well	Parameters	Initial	Optimal
LCBKK016	NL58	A	-0.017	-0.1 ± 5.9%
		n	1	0.3 ± 11.5%
		a	10	5925.8 ± 30.6%
		d	-5.9	-5.9 ± 12.3%
	NB54	A	-0.017	-0.1 ± 6.0%
		n	1	0.3 ± 8.5%
		a	10	10000 ± 28.0%
		d	-5.1	-5.1 ± 13.2%

Table 1.14: Parameters for the Pastas models for well nest LCBKK018.

Well Nest	Well	Parameters	Initial	Optimal
LCBKK018	PD82	A	-0.017	-0.07 ± 7.0%
		n	1	0.4 ± 17.1%
		a	10	2633.9 ± 37.8%
		d	-7.4	-7.4 ± 9.4%
		A	-0.017	-0.1 ± 6.0%

NB54

Table 1.15: Parameters for the Pastas models for well nest LCBKK020.

Well Nest	Well	Parameters	Initial	Optimal
LCBKK020	PD41	A	-0.017	-0.07 ± 7.1%
		n	1	0.3 ± 19.4%
		a	10	4251.9 ± 48.4%
		d	-6.0	-6.0 ± 10.9%
	NL56	A	-0.017	-0.2 ± 8.3%
		n	1	0.1 ± 38.6%
		a	10	5721.5 ± 125.9%
		d	-5.5	-5.5 ± 37.1%
	NB51	A	-0.017	-0.2 ± 4.9%
		n	1	0.2 ± 27.1%
		a	10	2967.6 ± 63.9%

		d	-4.6	-4.6 ± 31.2

Table 1.16: Parameters for the Pastas models for well nest LCBKK021.

Well Nest	Well	Parameters	Initial	Optimal
LCBKK021	PD36	A	-0.017	-0.08 ± 4.7%
		n	1	0.2 ± 6.1%
		a	10	10000 ± 24.0%
		d	-5.6	-5.6 ± 5.9%
	NL52	A	-0.017	-0.2 ± 3.6%
		n	1	0.1 ± 11.8%
		a	10	5747.5 ± 39.6%
		d	-5.0	-5.0 ± 18.5%
	NB45	A	-0.017	-0.2 ± 3.2%
		n	1	0.2 ± 4.6%
		a	10	10000 ± 18.9%
		d	-4.2	-4.2 ± 15.6

Table 1.17: Parameters for the Pastas models for well nest LCBKK026.

Well Nest	Well	Parameters	Initial	Optimal
LCBKK026	PD62	A	-0.017	-0.09 ± 2.1%
		n	1	0.4 ± 3.1%
		a	10	5875.0 ± 8.1%
		d	-5.1	-5.1 ± 4.0%
	NL18	A	-0.017	-0.1 ± 2.8%
		n	1	0.4 ± 7.3%
		a	10	7208.5 ± 16.6%
		d	-4.5	-4.5 ± 7.6%
	NB66	A	-0.017	-0.2 ± 2.9%
		n	1	0.8 ± 2.9%
		a	10	4455.9 ± 6.8%
		d	-3.8	-3.8 ± 13.2

Table 1.18: Parameters for the Pastas models for well nest LCBKK027.

Well Nest	Well	Parameters	Initial	Optimal

LCBKK027	PD57	A	-0.017	-0.09 ± 7.2%
		n	1	0.5 ± 13.8%
		a	10	3653.5 ± 30.5%
		d	-5.7	-5.7 ± 13.3%
	NL70	A	-0.017	-0.1 ± 7.5%
		n	1	0.4 ± 10.0%
		a	10	7169.5 ± 30.8%
		d	-5.3	-5.3 ± 15.2%
	NB62	A	-0.017	-0.2 ± 7.1%
		n	1	0.4 ± 7.5%
		a	10	10000 ± 25.6%
		d	-4.5	-4.5 ± 19.0

Table 1.19: Parameters for the Pastas models for well nest LCBKK036.

Well Nest	Well	Parameters	Initial	Optimal
LCBKK036	BK JICA8	A	-0.017	-0.04 ± 2.7%
		n	1	1.2 ± 12.4%
		a	10	1096.5 ± 15.1%
		d	-5.7	-5.7 ± 2.9%
	PD JICA7	A	-0.017	-0.2 ± 0.5%
		n	1	4.1 ± 7.9%
		a	10	181.9 ± 8.0%
		d	-5.3	-5.3 ± 3.0%
	NL JICA6	A	-0.017	-0.2 ± 0.7%
		n	1	5.2 ± 10.1%
		a	10	148.5 ± 10.2%
		d	-4.9	-4.9 ± 5.1%
	NB JICA5	A	-0.017	-0.2 ± 1.4%
		n	1	0.9 ± 9.5%
		a	10	1071 ± 11.4%
		d	-4.1	-4.1 ± 11.7%

Table 1.20: Parameters for the Pastas models for well nest LCBKK038.

Well Nest	Well	Parameters	Initial	Optimal

LCBKK038	BK1	A	-0.017	-0.05 ± 0.7%
		n	1	0.8 ± 2.4%
		a	10	1359.2 ± 3.6%
		d	-5.6	-5.6 ± 0.8%
	PD26 BKK009	A	-0.017	-0.08 ± 1.2%
		n	1	0.3 ± 4.1%
		a	10	3177.9 ± 8.8%
		d	-6.1	-6.1 ± 2.2%
	NL31	A	-0.017	-0.2 ± 2.1%
		n	1	0.2 ± 10.5%
		a	10	3226.3 ± 24.0%
		d	-5.5	-5.5 ± 7.9%
	NB36 BKK009	A	-0.017	-0.2 ± 1.4%
		n	1	0.1 ± 5.1%
		a	10	5626.5 ± 15.3%
		d	-4.7	-4.7 ± 6.3%

Table 1.21: Parameters for the Pastas models for well nest LCBKK041.

Well Nest	Well	Parameters	Initial	Optimal
LCBKK041	PD138	A	-0.017	-0.09 ± 7.2%
		n	1	0.5 ± 13.8%
		a	10	3653.5 ± 30.5%
		d	-5.7	-5.7 ± 13.3%
	NL140	A	-0.017	-0.1 ± 7.5%
		n	1	0.4 ± 10.0%
		a	10	7169.5 ± 30.8%
		d	-5.3	-5.3 ± 15.2%
	NB139	A	-0.017	-0.2 ± 7.1%
		n	1	0.4 ± 7.5%
		a	10	10000 ± 25.6%
		d	-4.5	-4.5 ± 19.0

Table 1.22: Parameters for the Pastas models for well nest LCNBI003.

Well Nest	Well	Parameters	Initial	Optimal

LCNBI003	NL41	A	-0.017	-0.1 ± 3.3%
		n	1	0.2 ± 8.5%
		a	10	7206.8 ± 25.0%
		d	-5.4	-5.4 ± 6.6%
	NB24	A	-0.017	-0.1 ± 6.5%
		n	1	0.2 ± 15.9%
		a	10	10000 ± 52.9%
		d	-4.5	-4.5 ± 17.5%

Table 1.23: Parameters for the Pastas models for well nest LCNBI007.

Well Nest	Well	Parameters	Initial	Optimal
LCNBI007	PD56	A	-0.017	-0.06 ± 4.5%
		n	1	0.5 ± 9.0%
		a	10	3547.6 ± 19.0%
		d	-6.1	-6.1 ± 5.8%
	NB63	A	-0.017	-0.1 ± 6.2%
		n	1	0.3 ± 7.5%
		a	10	10000 ± 26.4%
		d	-4.8	-4.8 ± 13.1%

Table 1.24: Parameters for the Pastas models for well nest LCSPK007.

Well Nest	Well	Parameters	Initial	Optimal
LCSPK007	PD22	A	-0.017	-0.1 ± 5.1%
		n	1	0.01 ± 0%
		a	10	0.01 ± 0%
		d	-7.0	-7.0 ± 12.5%
	NB30	A	-0.017	-0.1 ± 5.0%
		n	1	0.3 ± 12.6%
		a	10	5398.7 ± 29.8%
		d	-5.7	-5.7 ± 13.7%

Table 1.25: Parameters for the Pastas models for well nest LCSPK009.

Well Nest	Well	Parameters	Initial	Optimal
LCSPK009	NL67	A	-0.017	-0.1 ± 1.6%

		n	1	0.3 ± 4.8%
		a	10	4043.8 ± 10.8%
		d	-5.4	-5.4 ± 4.3%
	NB53	A	-0.017	-0.1 ± 2.1%
		n	1	0.2 ± 5.2%
		a	10	7570.8 ± 15.7%
		d	-4.6	-4.6 ± 5.3%

1.3.2 Groundwater in the Clay Layers and Subsidence Modeling

Table 1.26: Thicknesses of each clay and aquifer layer for each well nest.

Well Nest	Clay	BK	Clay	PD	Clay	NL	Clay	NB
LCBKK003	51	20	24	28	33	25	3	21
LCBKK005	22	34	19.5	52.5	17	51	19	11
LCBKK006	33	21	30	42	13	59	5	18
LCBKK007	25.8	41.2	16.5	50.3	17.4	56.1	6.1	6.7
LCBKK009	38.1	15.2	6.7	65.2	12.5	19.8	4.3	35.9
LCBKK011	27	58	23.6	47.2	13.5	27	3.7	34.2
LCBKK012	25.1	19.8	45.2	22.5	60	80	20	60
LCBKK013	38.5	40.1	20.9	17.4	10.4	55.7	8.9	19.1
LCBKK014	36	15	40	35	3	65	30	6
LCBKK015	25.8	41.2	16.5	50.3	17.4	56.1	6.1	6.7
LCBKK016	27.7	19.3	36	14.2	9.4	36.3	19.2	72.5
LCBKK018	27	27	12	48	26.2	20.8	11.3	9.7
LCBKK020	24.5	21	39.5	58	18.5	41.5	8	21
LCBKK021	18	27	45	33	39	54	3	9
LCBKK026	25.5	30	10	34.5	3	60	3	70
LCBKK027	42	6	23	13.5	24	34.5	51	18.5
LCBKK036	28	21	47.2	15.8	28	28	37	46
LCBKK038	22	34	19.5	52.5	17	51	19	11
LCBKK041	22	34	19.5	52.5	17	51	19	11
LCNBI003	25	20	28	14	9	55	30	52
LCNBI007	23.2	39	7.9	40.9	7.5	22.3	17.4	76.5
LCSPK007	40	10	70	30	20	20	20	60

LCSPK009	25.5	37.5	33.5	7	15	21.5	11.5	55.5
Min	18	6	6.7	7	3	19.8	3	6
Max	42	58	70	65.2	60	65	51	76.5
Average	29.2	27.4	27.6	35.8	18.7	42.2	15.5	31.8

Table 1.27: Parameter ranges used during calibration (Japan International Cooperation Agency 1995)

	S_v [1/m]		S_e [1/m]		K_v [m/day]	
	Min	Max	Min	Max	Min	Max
BK Clay	5e-4	7e-2	3e-6	6e-1	8e-7	1e-3
BK Aquifer	-	-	2e-7	2e-4	-	-
PD Clay	4e-5	8e-4	2e-7	1.6e-2	2e-7	5e-5
PD Aquifer	-	-	2e-7	2e-4	-	-
NL Clay	9e-5	3e-4	4.5e-7	6e-3	2e-7	6e-6
NL Aquifer	-	-	2e-7	2e-4	-	-
NB Clay	3e-5	3e-4	1.5e-7	6e-3	5e-8	1e-5
NB Aquifer	-	-	2e-7	2e-4	-	-

Table 1.28: Final parameter ranges after calibration

Final						
	S_v [1/m]		S_e [1/m]		K_v [m/day]	
	Min	Max	Min	Max	Min	Max
BK Clay	3e-4	1e-2	2e-6	5e-4	4e-7	2e-4
BK Aquifer	-	-	2e-7	5e-5	-	-
PD Clay	2e-4	8e-4	2e-6	4e-5	4e-7	6e-5
PD Aquifer	-	-	2e-7	5e-5	-	-
NL Clay	2e-4	3e-4	2e-6	2e-5	4e-7	6e-5
NL Aquifer	-	-	2e-7	5e-5	-	-
NB Clay	2e-4	3e-4	2e-6	2e-5	5e-8	1e-5
NB Aquifer	-	-	1e-7	2e-5	-	-

1.4 Groundwater in the Aquifer Time Series Models Results

Note that missing intervals of groundwater data were filled by assuming constant relationships with the closest aquifers. For example, if the difference between groundwater levels in PD and NL averaged to 15 m i.e. PD has, on average, groundwater levels 15 m higher, then missing data in PD can be calculated from NL assuming a difference of 15 m.

1.4.1 BKK003

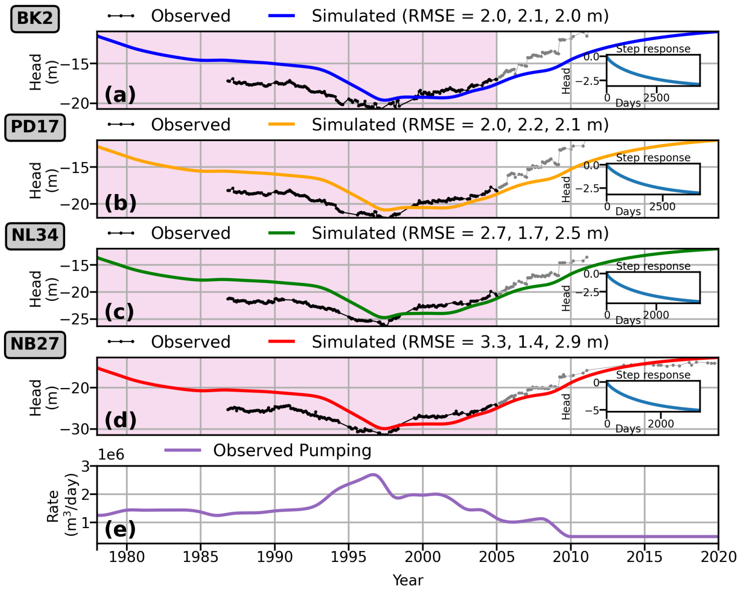

Figure 1.3: Results for Pastas time series models for well nest (LC)BKK003. The calibration period 1978-2005 is shown in the pink box. Validation is conducted for 2005-2020. RMSEs are provided for the calibration, validation, and entire time period.

1.4.2 BKK005

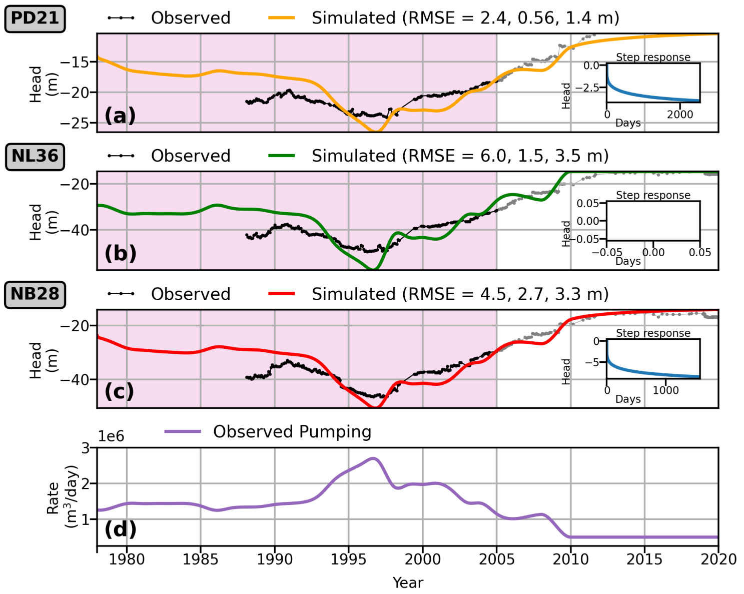

Figure 1.4: Results for Pastas time series models for well nest (LC)BKK005. The calibration period 1978-2005 is shown in the pink box. Validation is conducted for 2005-2020. RMSEs are provided for the calibration, validation, and entire time period.

1.4.3 BKK006

Note than an observation well did not exist in the BK aquifer for well nest (LC)BKK006. However, (LC)BKK006 is located about 8 km away from (LC)BKK003. The relationship between groundwater levels in BK2 and PD17 in (LC)BKK003 was assumed between BK and PD aquifers in (LC)BKK006.

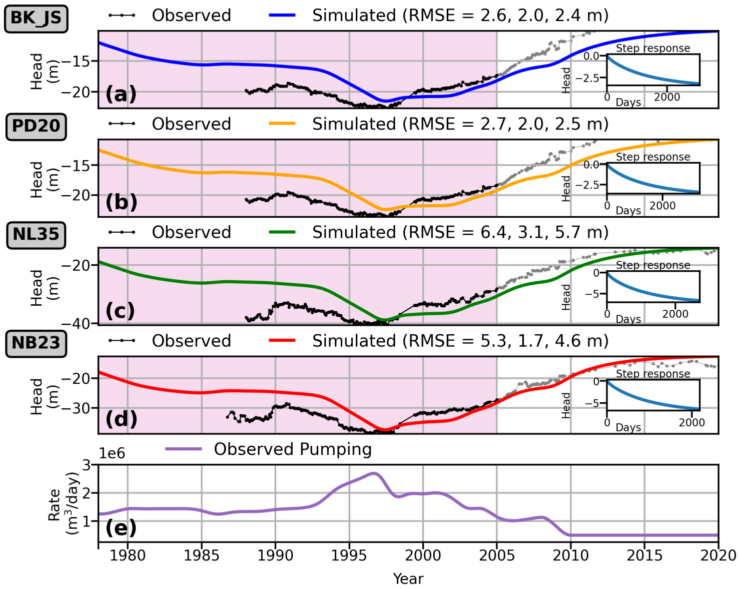

Figure 1.5: Results for Pastas time series models for well nest (LC)BKK006. The calibration period 1978-2005 is shown in the pink box. Validation is conducted for 2005-2020. RMSEs are provided for the calibration, validation, and entire time period.

1.4.4 BKK007

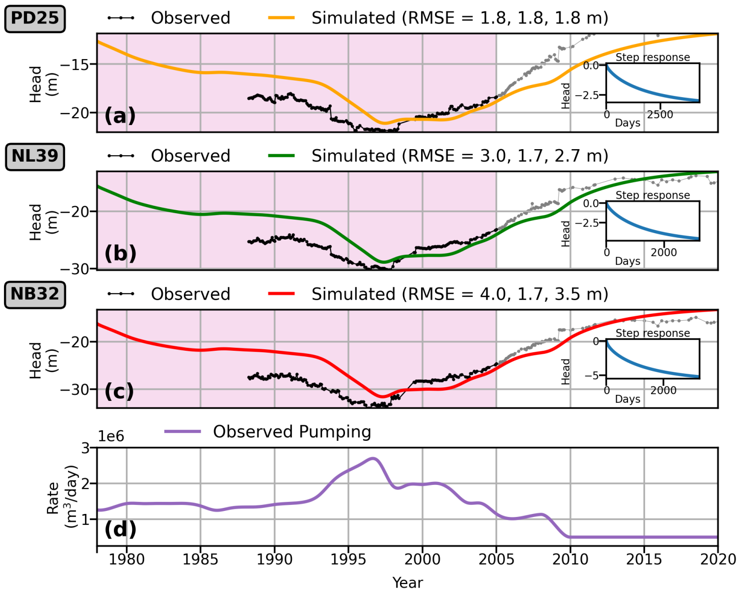

Figure 1.6: Results for Pastas time series models for well nest (LC)BKK007. The calibration period 1978-2005 is shown in the pink box. Validation is conducted for 2005-2020. RMSEs are provided for the calibration, validation, and entire time period.

1.4.5 BKK009

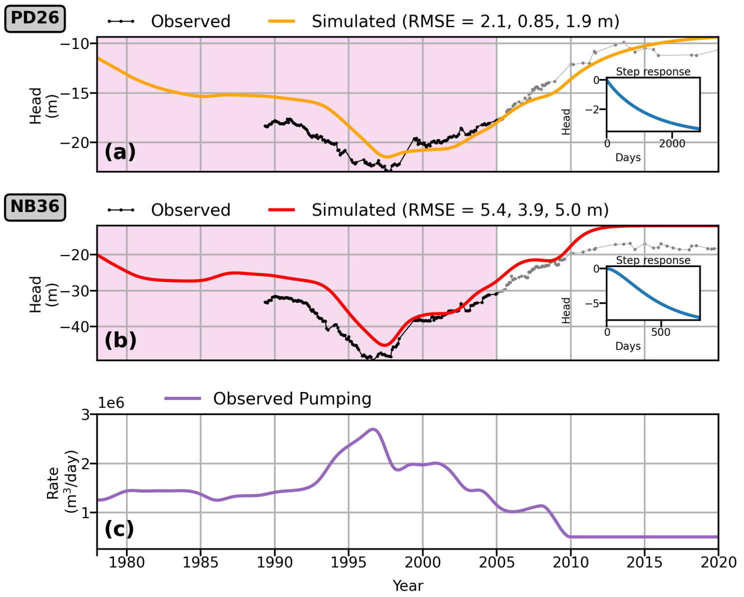

Figure 1.7: Results for Pastas time series models for well nest (LC)BKK009. The calibration period 1978-2005 is shown in the pink box. Validation is conducted for 2005-2020. RMSEs are provided for the calibration, validation, and entire time period.

1.4.6 BKK011

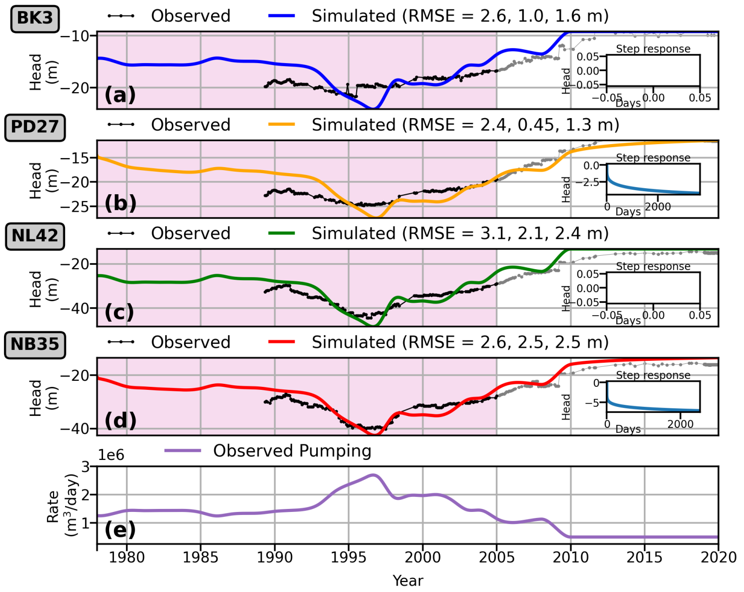

Figure 1.8: Results for Pastas time series models for well nest (LC)BKK011. The calibration period 1978-2005 is shown in the pink box. Validation is conducted for 2005-2020. RMSEs are provided for the calibration, validation, and entire time period.

1.4.7 BKK012

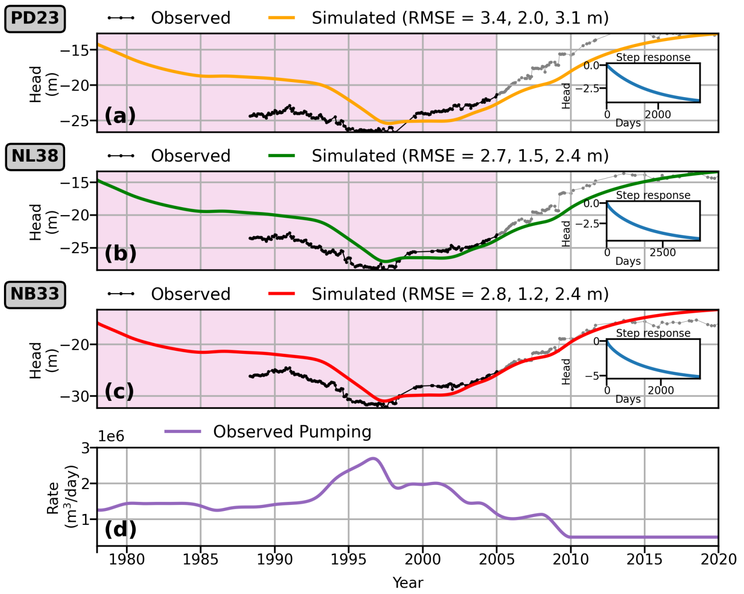

Figure 1.9: Results for Pastas time series models for well nest (LC)BKK012. The calibration period 1978-2005 is shown in the pink box. Validation is conducted for 2005-2020. RMSEs are provided for the calibration, validation, and entire time period.

1.4.8 BKK013

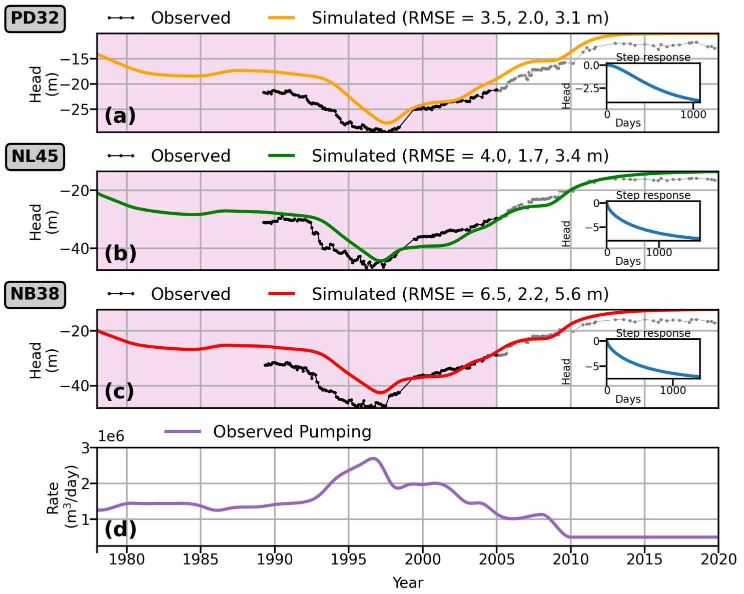

Figure 1.10: Results for Pastas time series models for well nest (LC)BKK013. The calibration period 1978-2005 is shown in the pink box. Validation is conducted for 2005-2020. RMSEs are provided for the calibration, validation, and entire time period.

1.4.9 BKK014

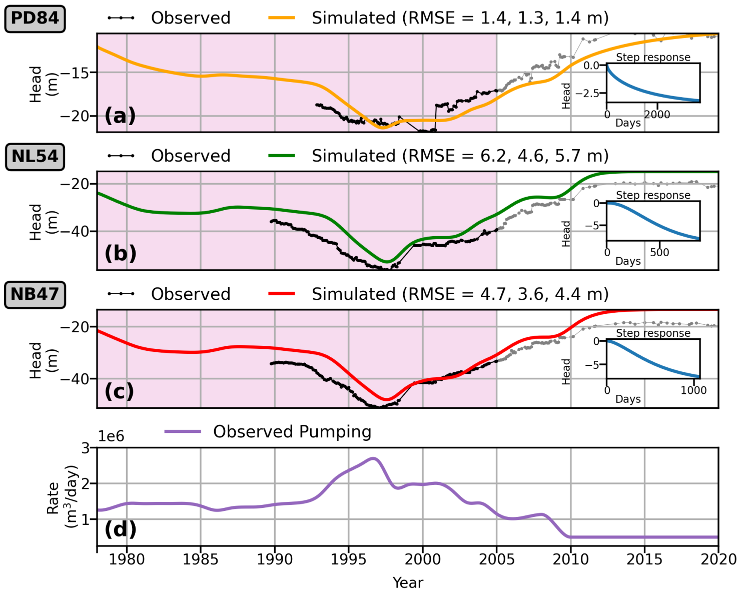

Figure 1.11: Results for Pastas time series models for well nest (LC)BKK014. The calibration period 1978-2005 is shown in the pink box. Validation is conducted for 2005-2020. RMSEs are provided for the calibration, validation, and entire time period.

1.4.10 BKK015

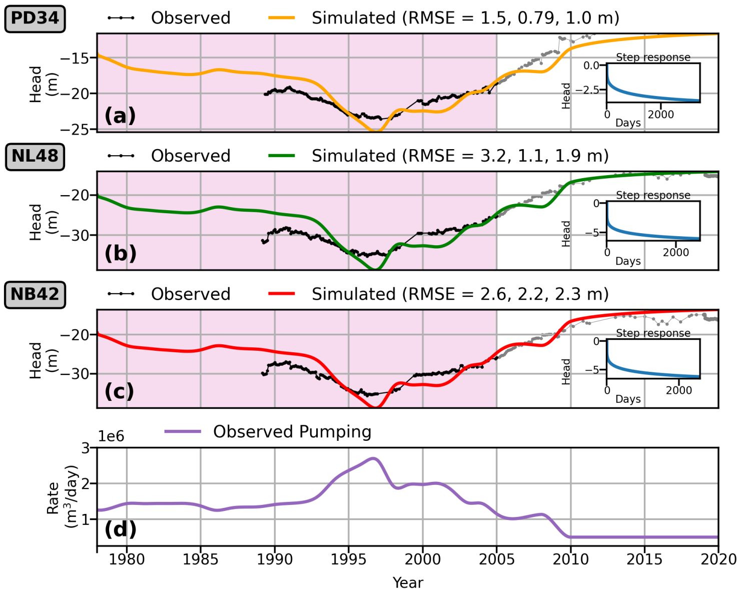

Figure 1.12: Results for Pastas time series models for well nest (LC)BKK015. The calibration period 1978-2005 is shown in the pink box. Validation is conducted for 2005-2020. RMSEs are provided for the calibration, validation, and entire time period.

1.4.11 BKK016

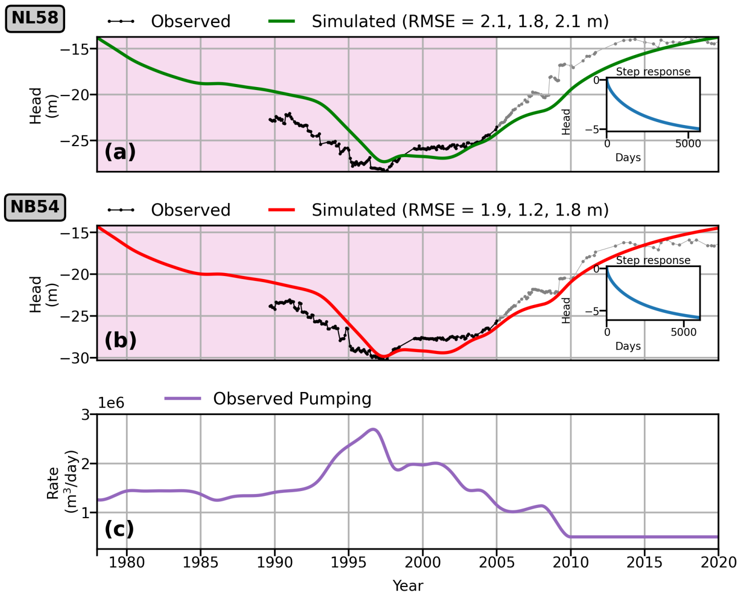

Figure 1.13: Results for Pastas time series models for well nest (LC)BKK016. The calibration period 1978-2005 is shown in the pink box. Validation is conducted for 2005-2020. RMSEs are provided for the calibration, validation, and entire time period.

1.4.12 BKK018

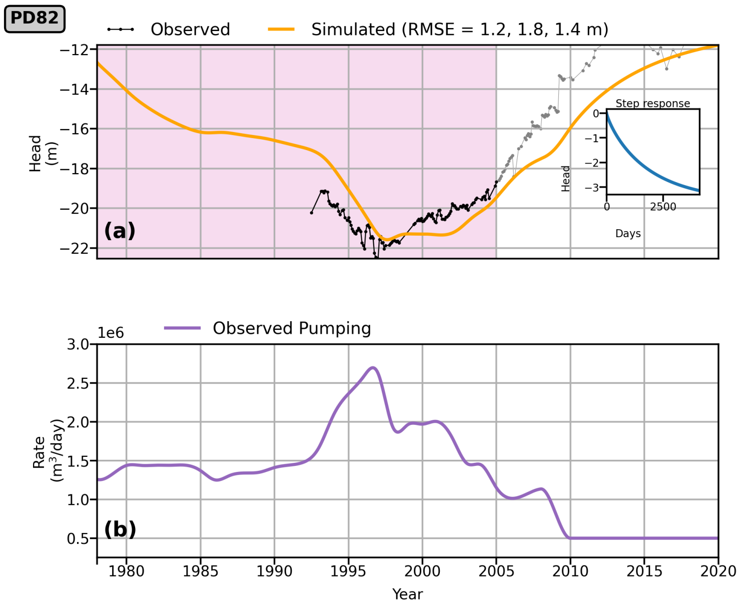

Figure 1.14: Results for Pastas time series models for well nest (LC)BKK018. The calibration period 1978-2005 is shown in the pink box. Validation is conducted for 2005-2020. RMSEs are provided for the calibration, validation, and entire time period.

1.4.13 BKK020

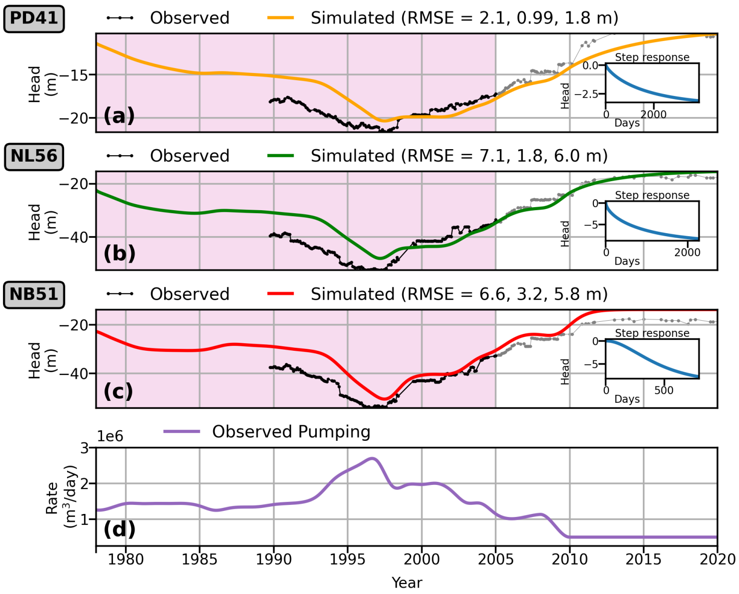

Figure 1.15: Results for Pastas time series models for well nest (LC)BKK020. The calibration period 1978-2005 is shown in the pink box. Validation is conducted for 2005-2020. RMSEs are provided for the calibration, validation, and entire time period.

1.4.14 BKK021

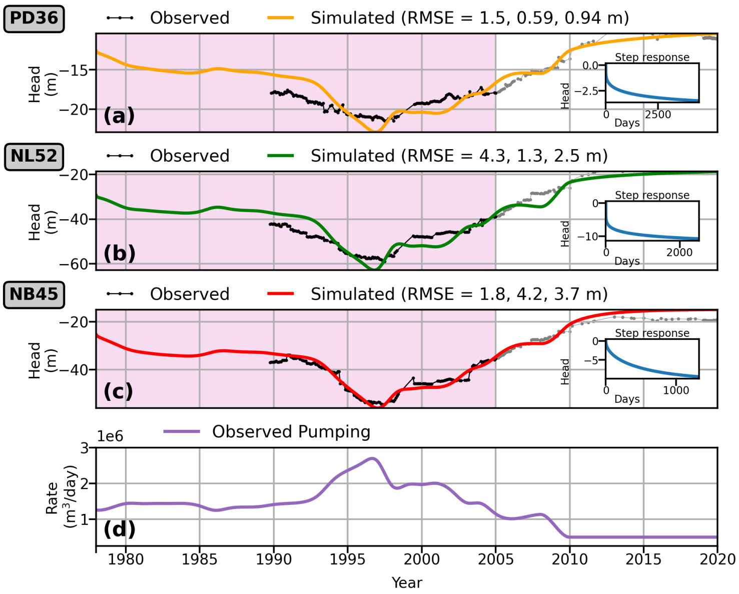

Figure 1.16: Results for Pastas time series models for well nest (LC)BKK021. The calibration period 1978-2005 is shown in the pink box. Validation is conducted for 2005-2020. RMSEs are provided for the calibration, validation, and entire time period.

1.4.15 BKK026

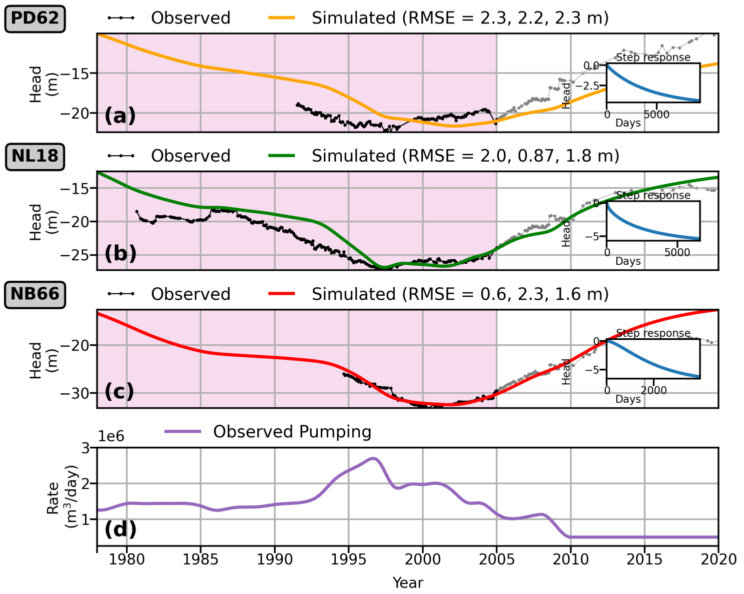

Figure 1.17: Results for Pastas time series models for well nest (LC)BKK026. The calibration period 1978-2005 is shown in the pink box. Validation is conducted for 2005-2020. RMSEs are provided for the calibration, validation, and entire time period.

1.4.16 BKK027

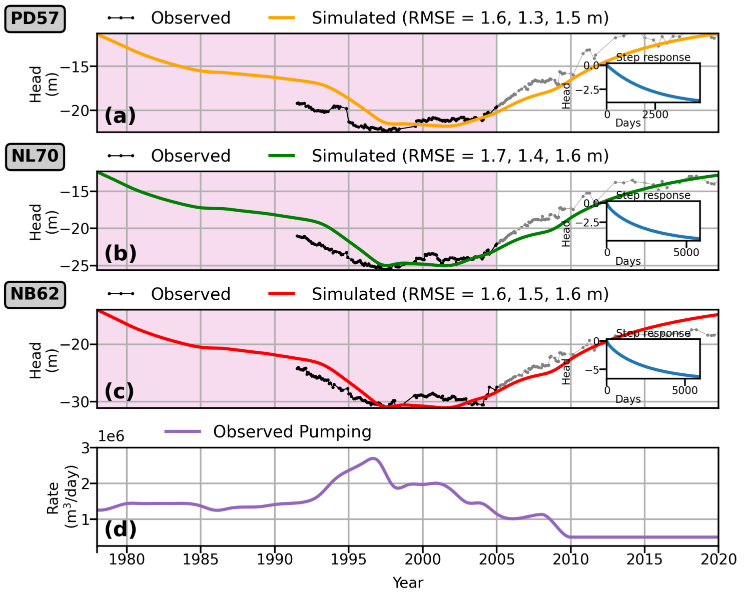

Figure 1.18: Results for Pastas time series models for well nest (LC)BKK027. The calibration period 1978-2005 is shown in the pink box. Validation is conducted for 2005-2020. RMSEs are provided for the calibration, validation, and entire time period.

1.4.17 BKK036

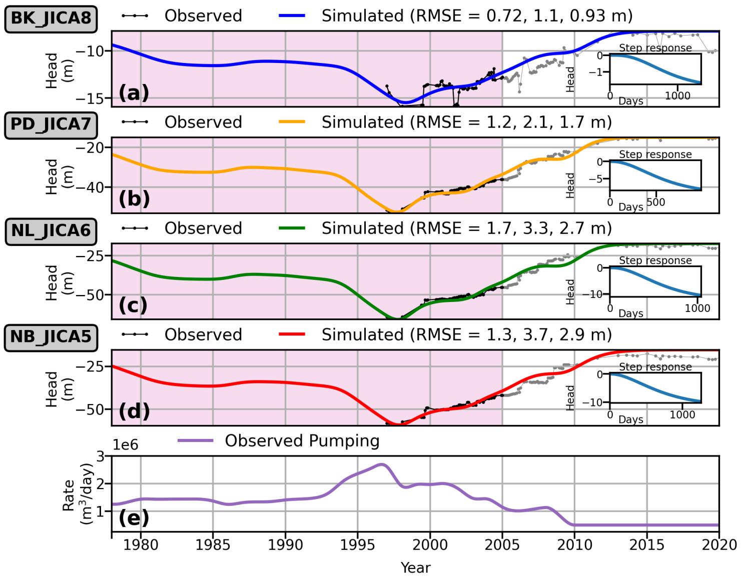

Figure 1.19: Results for Pastas time series models for well nest (LC)BKK036. The calibration period 1978-2005 is shown in the pink box. Validation is conducted for 2005-2020. RMSEs are provided for the calibration, validation, and entire time period.

1.4.18 BKK038

Note than observation wells do not exist in the PD and NB aquifers for well nest (LC)BKK038. However, (LC)BKK038 is located about 6 km away from (LC)BKK009. Groundwater levels in PD26 and NB36 were used as proxies for missing observations in the PD and NB aquifers for (LC)BKK038.

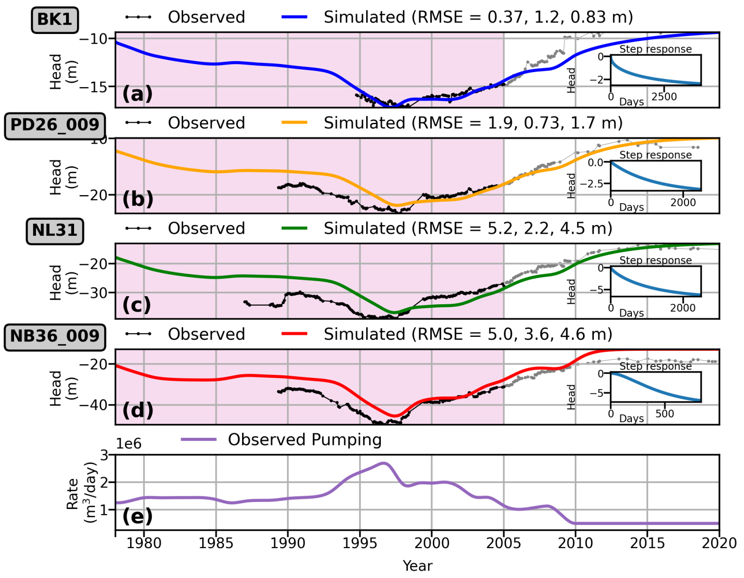

Figure 1.20: Results for Pastas time series models for well nest (LC)BKK038. The calibration period 1978-2005 is shown in the pink box. Validation is conducted for 2005-2020. RMSEs are provided for the calibration, validation, and entire time period.

1.4.19 BKK041

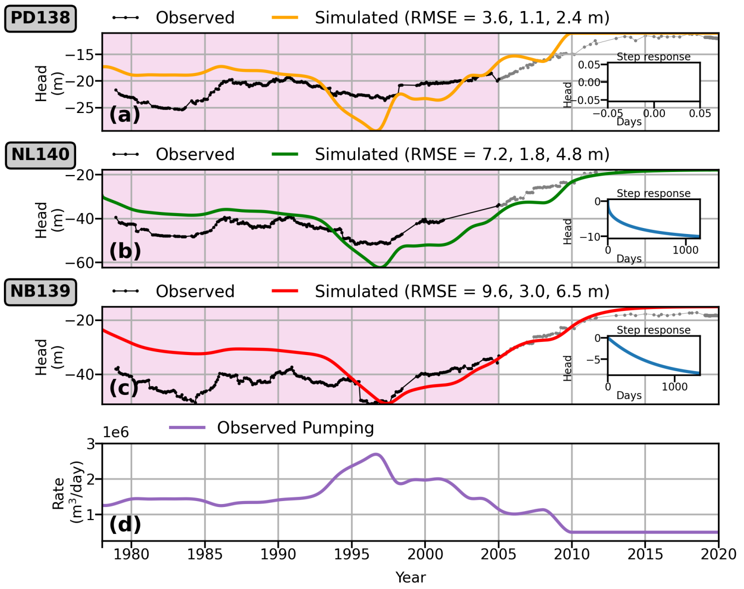

Figure 1.21: Results for Pastas time series models for well nest (LC)BKK041. The calibration period 1978-2005 is shown in the pink box. Validation is conducted for 2005-2020. RMSEs are provided for the calibration, validation, and entire time period.

1.4.20 NBI003

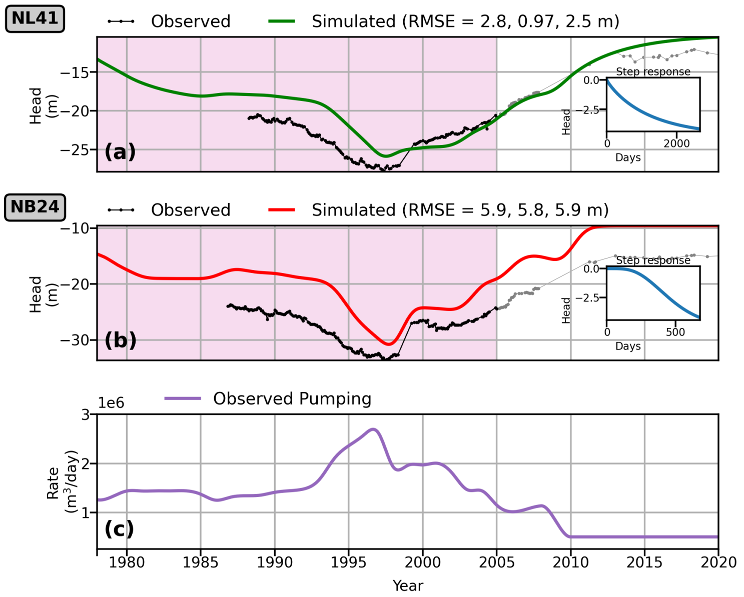

Figure 1.22: Results for Pastas time series models for well nest NBI003. The calibration period 1978-2005 is shown in the pink box. Validation is conducted for 2005-2020. RMSEs are provided for the calibration, validation, and entire time period.

1.4.21 NBI007

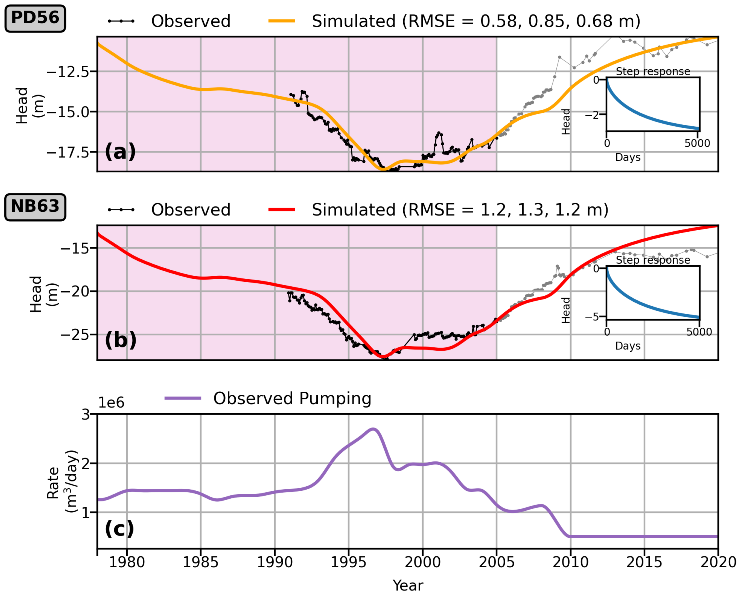

Figure 1.23: Results for Pastas time series models for well nest NBI007. The calibration period 1978-2005 is shown in the pink box. Validation is conducted for 2005-2020. RMSEs are provided for the calibration, validation, and entire time period.

1.4.22 SPK007

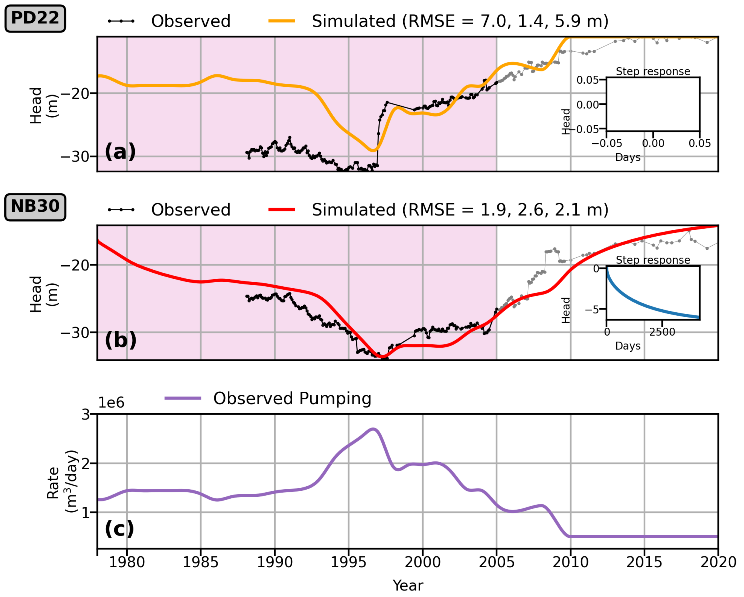

Figure 1.24: Results for Pastas time series models for well nest SPK007. The calibration period 1978-2005 is shown in the pink box. Validation is conducted for 2005-2020. RMSEs are provided for the calibration, validation, and entire time period.

1.4.23 SPK009

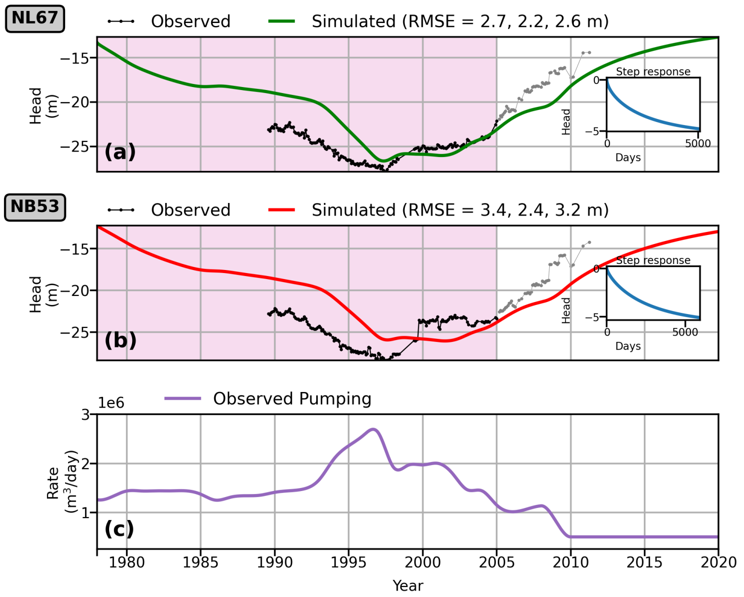

Figure 1.25: Results for Pastas time series models for well nest SPK009. The calibration period 1978-2005 is shown in the pink box. Validation is conducted for 2005-2020. RMSEs are provided for the calibration, validation, and entire time period.

1.5 Subsidence Model Results

1.5.1 BKK003

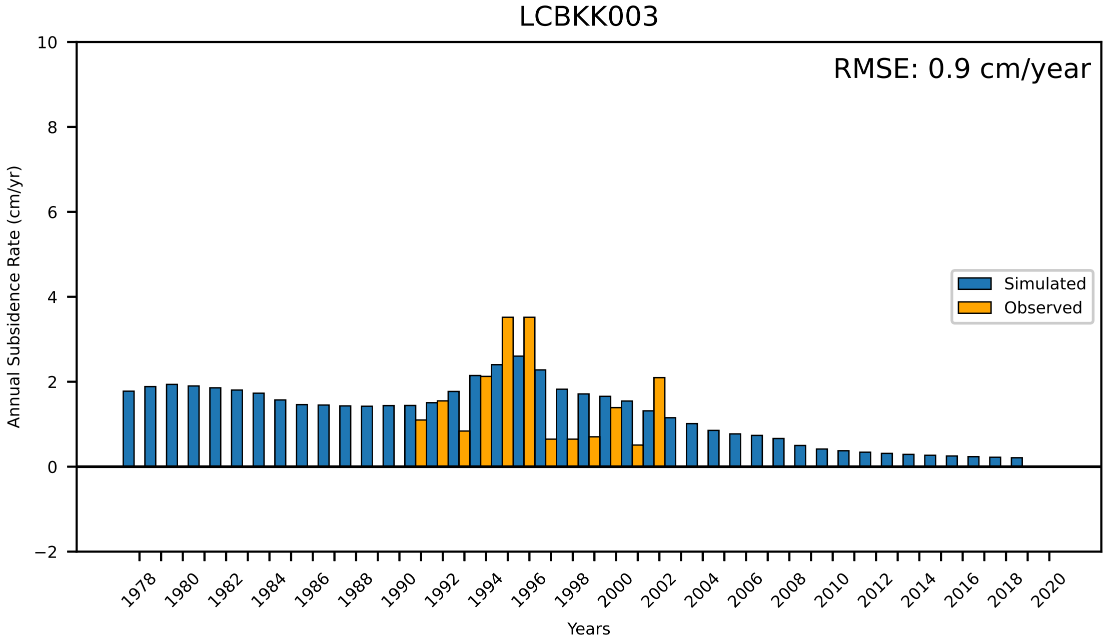

Figure 1.26: Simulated subsidence rates (blue) plotted against observed subsidence rates (orange) for well nest (LC)BKK003.

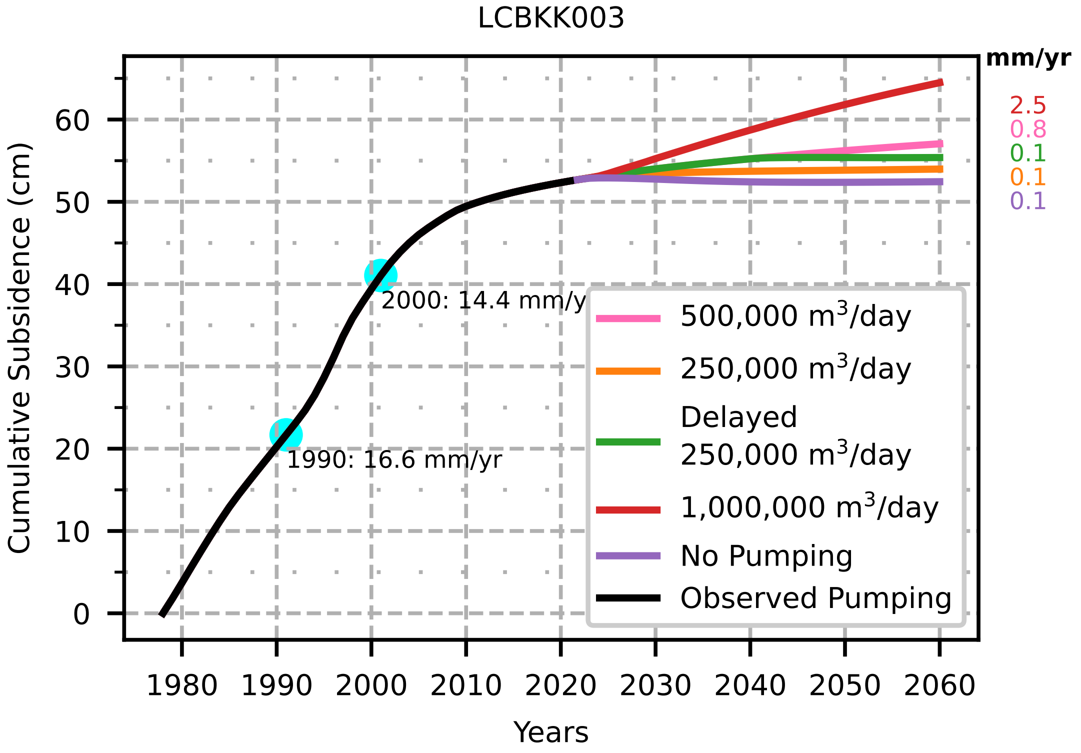

Figure 1.27: Simulated cumulative subsidence for 1978-2060 for well nest (LC)BKK003. For 2020-2060, cumulative subsidence is plotted for several pumping scenarios. The 2060 subsidence rate is shown in the upper right hand corner for each scenario.

1.5.2 BKK005

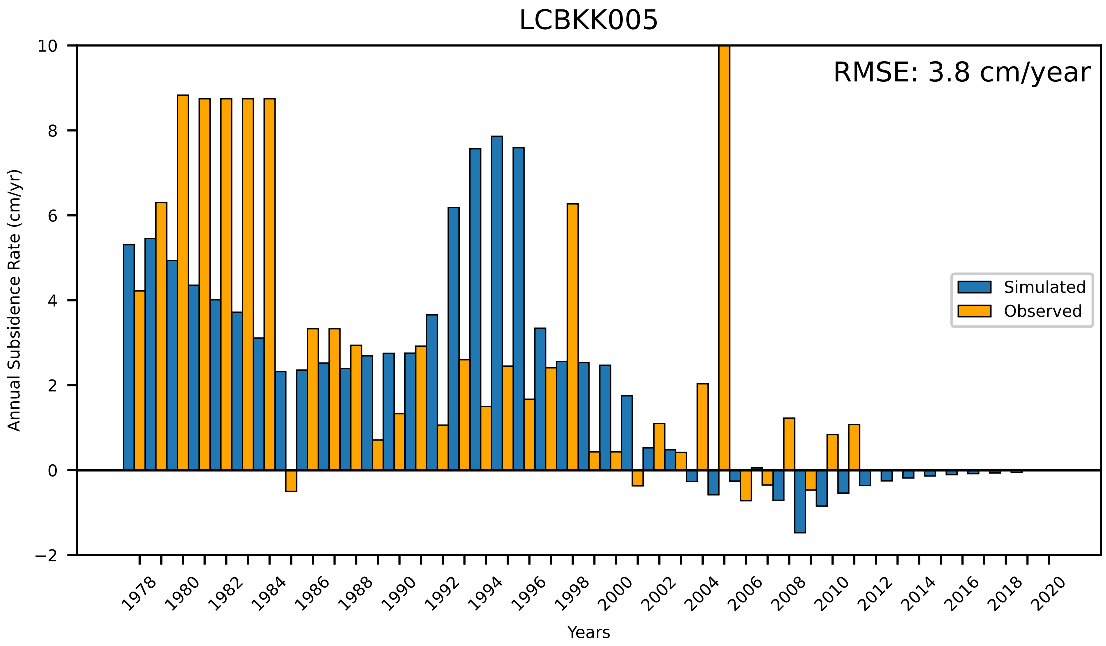

Figure 1.28: Simulated subsidence rates (blue) plotted against observed subsidence rates (orange) for well nest (LC)BKK005.

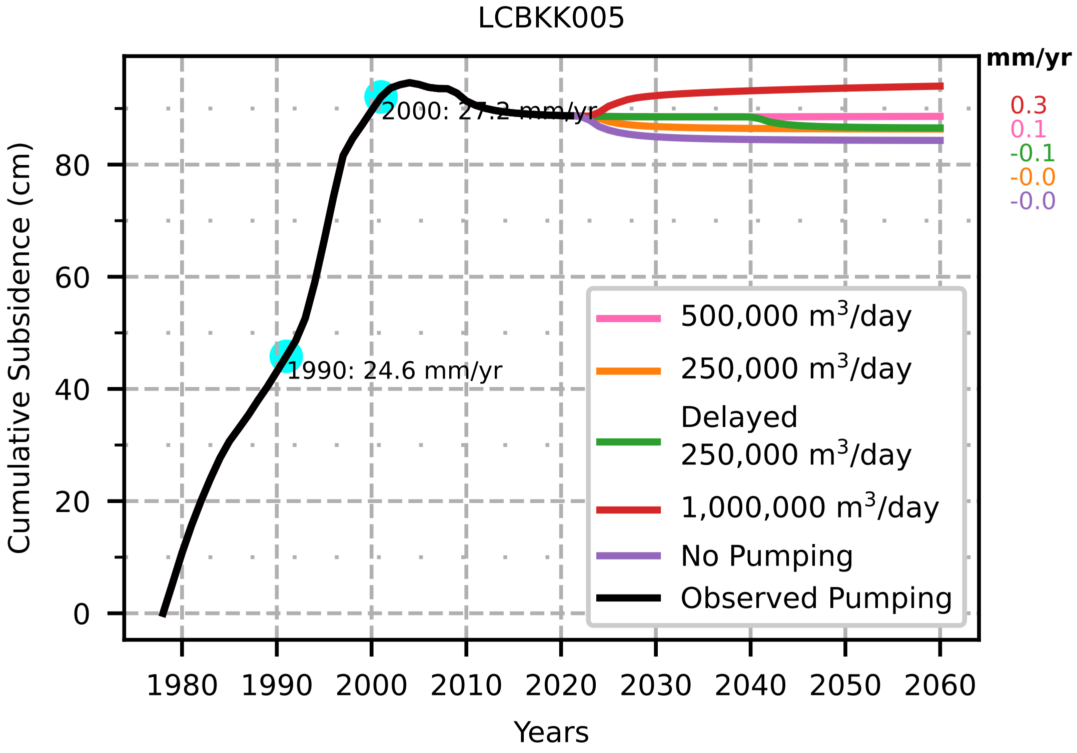

Figure 1.29: Simulated cumulative subsidence for 1978-2060 for well nest (LC)BKK005. For 2020-2060, cumulative subsidence is plotted for several pumping scenarios. The 2060 subsidence rate is shown in the upper right hand corner for each scenario.

1.5.3 BKK006

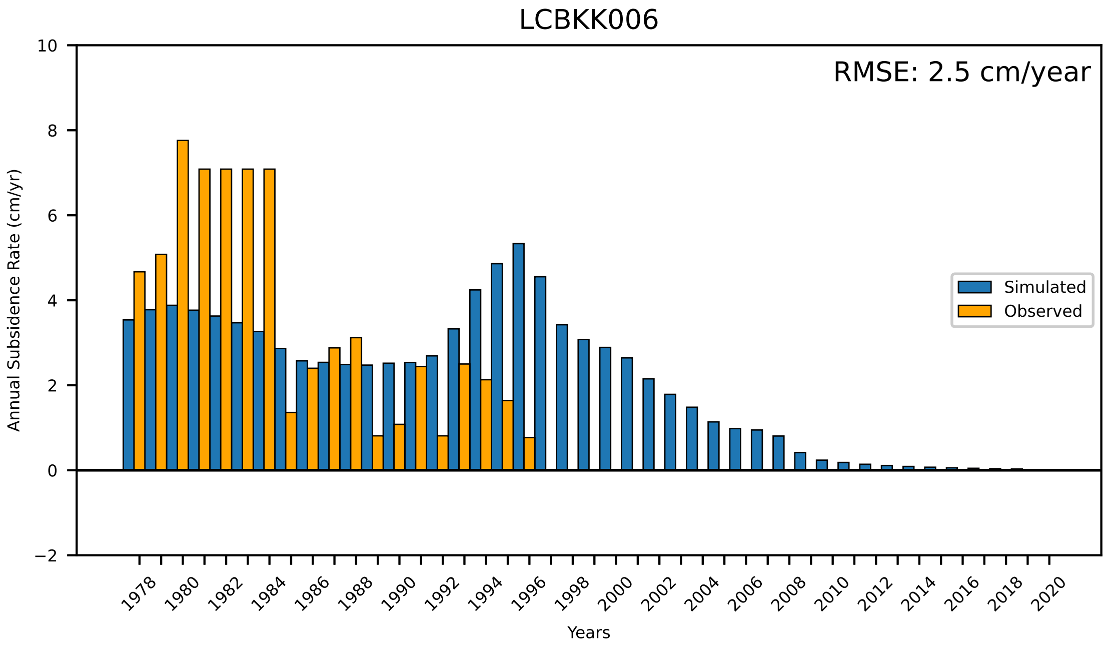

Figure 1.30: Simulated subsidence rates (blue) plotted against observed subsidence rates (orange) for well nest (LC)BKK006.

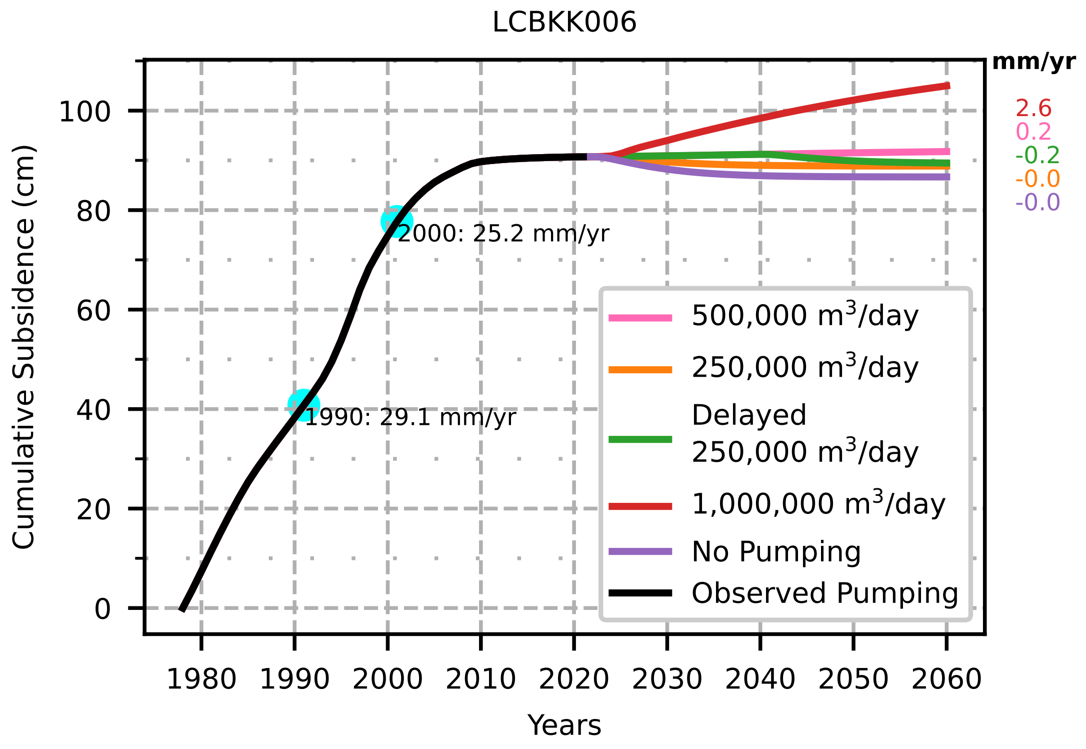

Figure 1.31: Simulated cumulative subsidence for 1978-2060 for well nest (LC)BKK006. For 2020-2060, cumulative subsidence is plotted for several pumping scenarios. The 2060 subsidence rate is shown in the upper right hand corner for each scenario.

1.5.4 BKK007

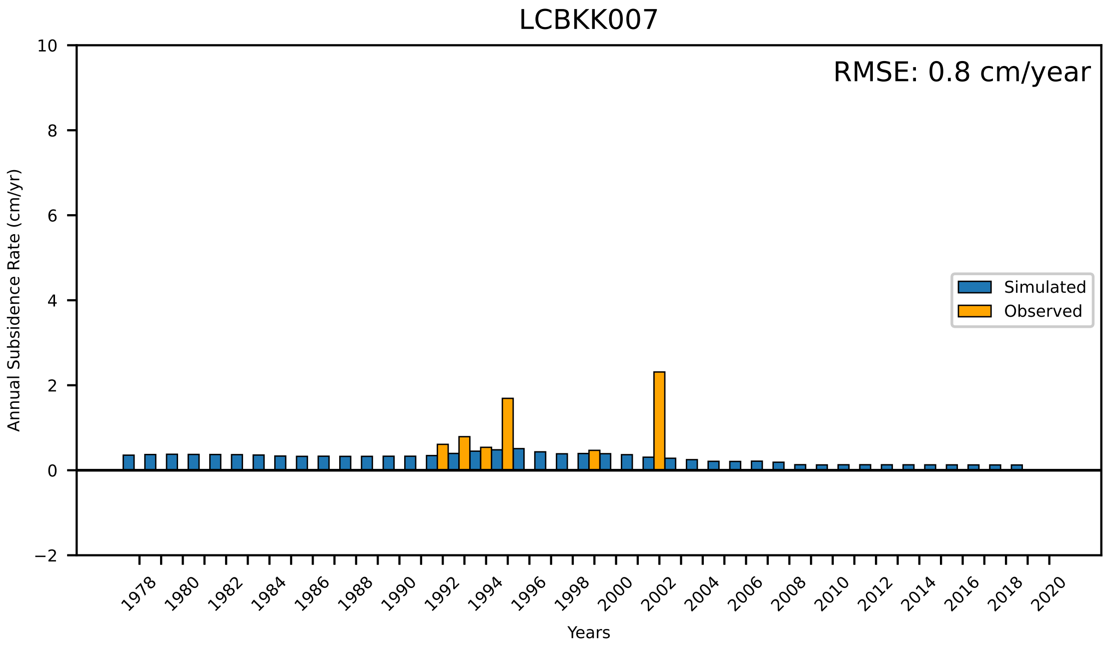

Figure 1.32: Simulated subsidence rates (blue) plotted against observed subsidence rates (orange) for well nest (LC)BKK007.

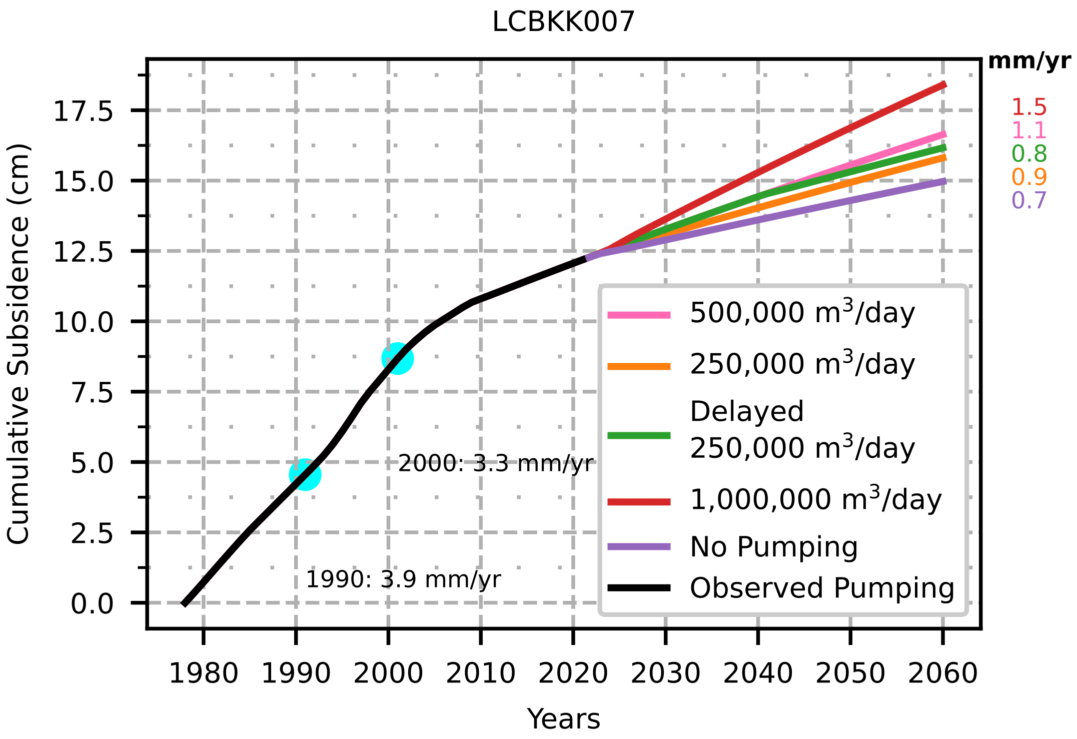

Figure 1.33: Simulated cumulative subsidence for 1978-2060 for well nest (LC)BKK007. For 2020-2060, cumulative subsidence is plotted for several pumping scenarios. The 2060 subsidence rate is shown in the upper right hand corner for each scenario.

1.5.5 BKK009

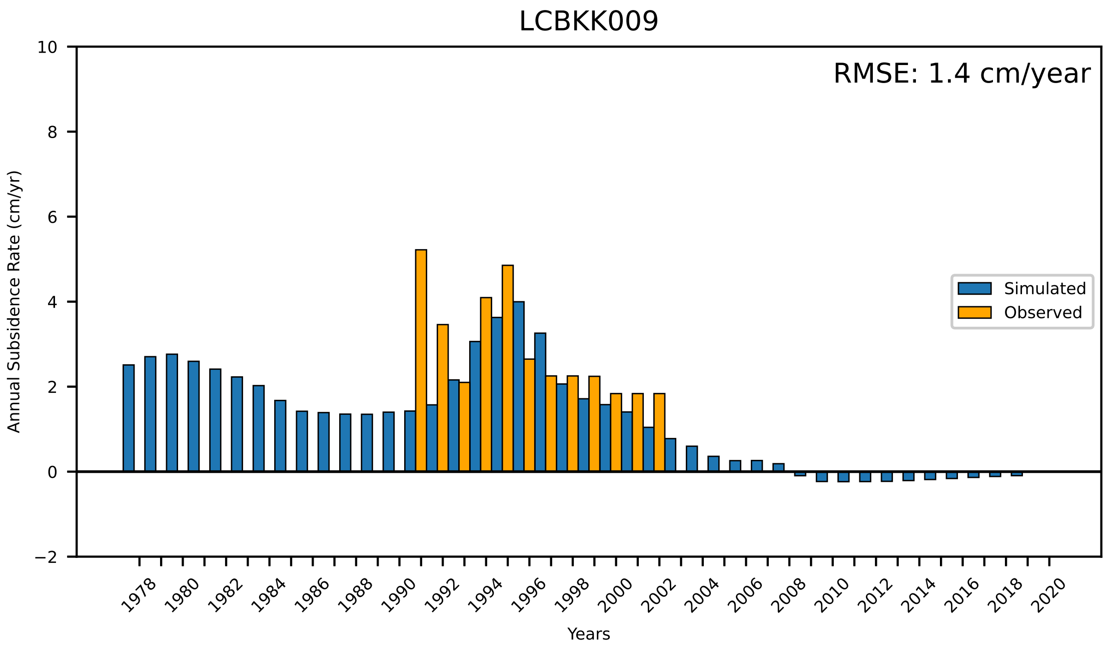

Figure 1.34: Simulated subsidence rates (blue) plotted against observed subsidence rates (orange) for well nest (LC)BKK009.

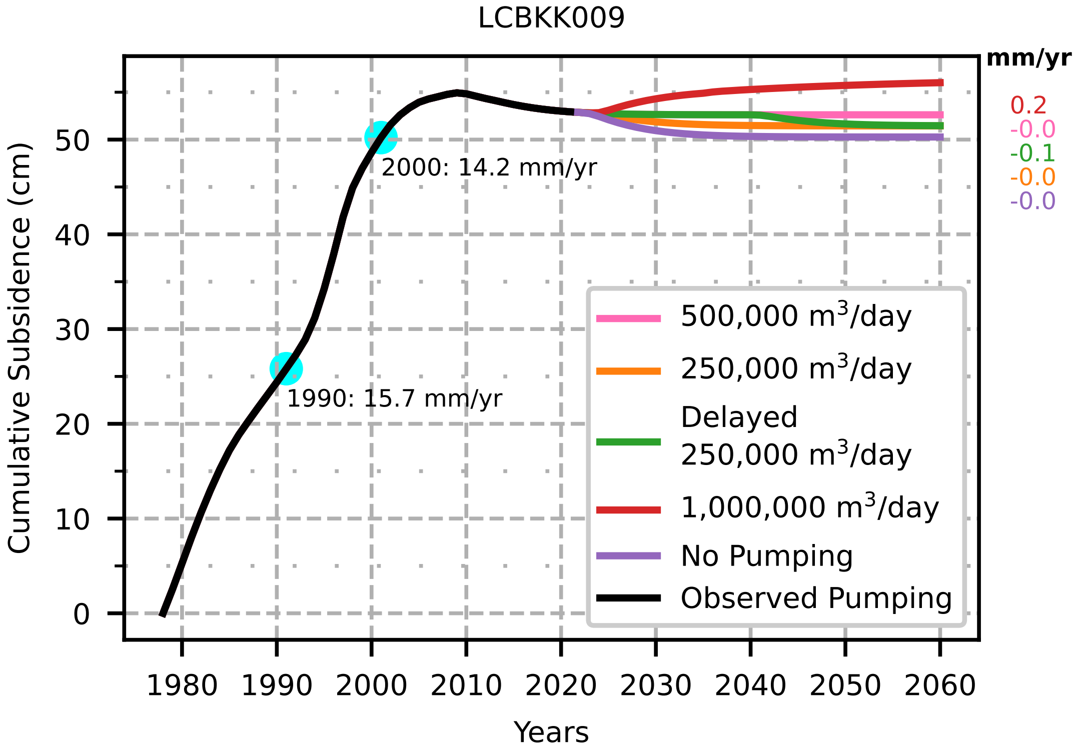

Figure 1.35: Simulated cumulative subsidence for 1978-2060 for well nest (LC)BKK009. For 2020-2060, cumulative subsidence is plotted for several pumping scenarios. The 2060 subsidence rate is shown in the upper right hand corner for each scenario.

1.5.6 BKK011

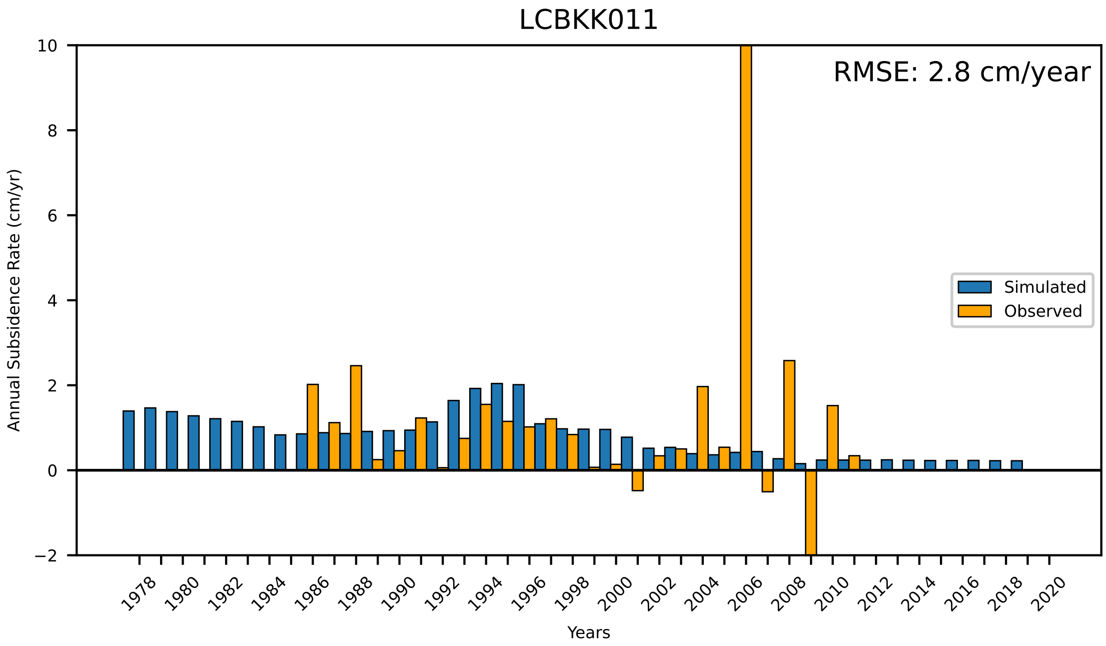

Figure 1.36: Simulated subsidence rates (blue) plotted against observed subsidence rates (orange) for well nest (LC)BKK011.

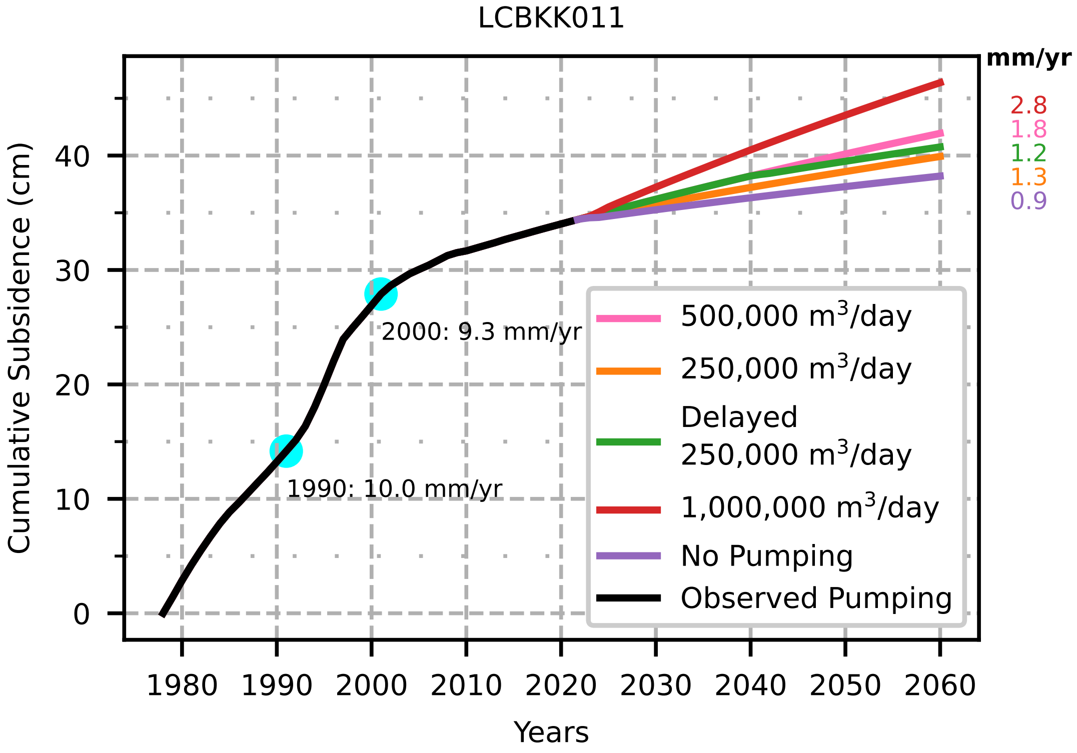

Figure 1.37: Simulated cumulative subsidence for 1978-2060 for well nest (LC)BKK011. For 2020-2060, cumulative subsidence is plotted for several pumping scenarios. The 2060 subsidence rate is shown in the upper right hand corner for each scenario.

1.5.7 BKK012

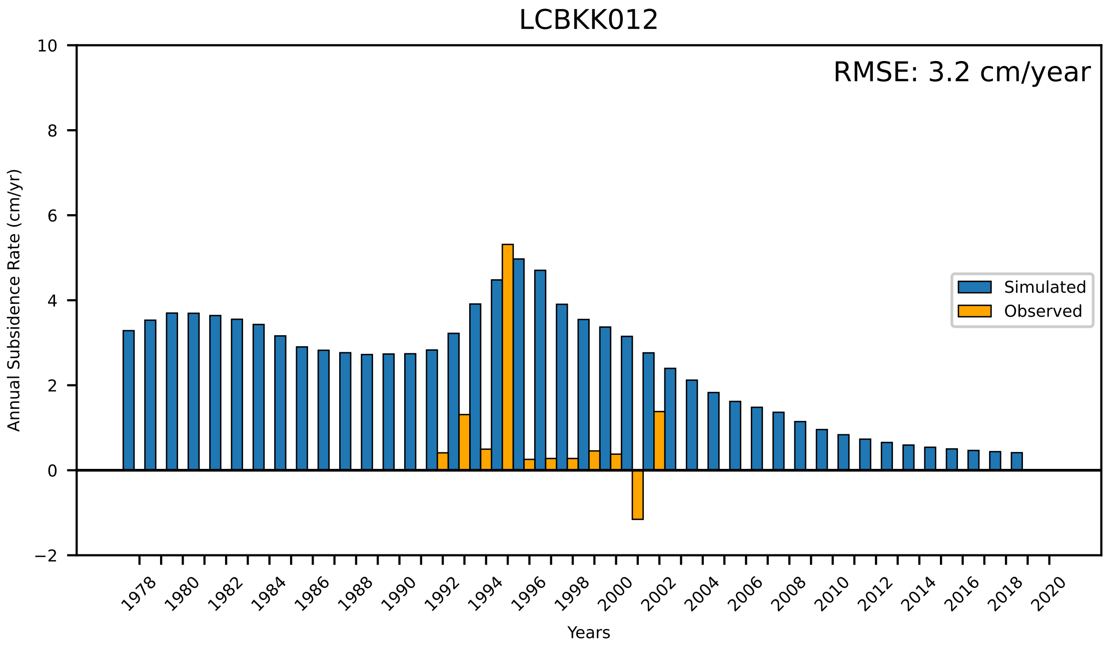

Figure 1.38: Simulated subsidence rates (blue) plotted against observed subsidence rates (orange) for well nest (LC)BKK012.

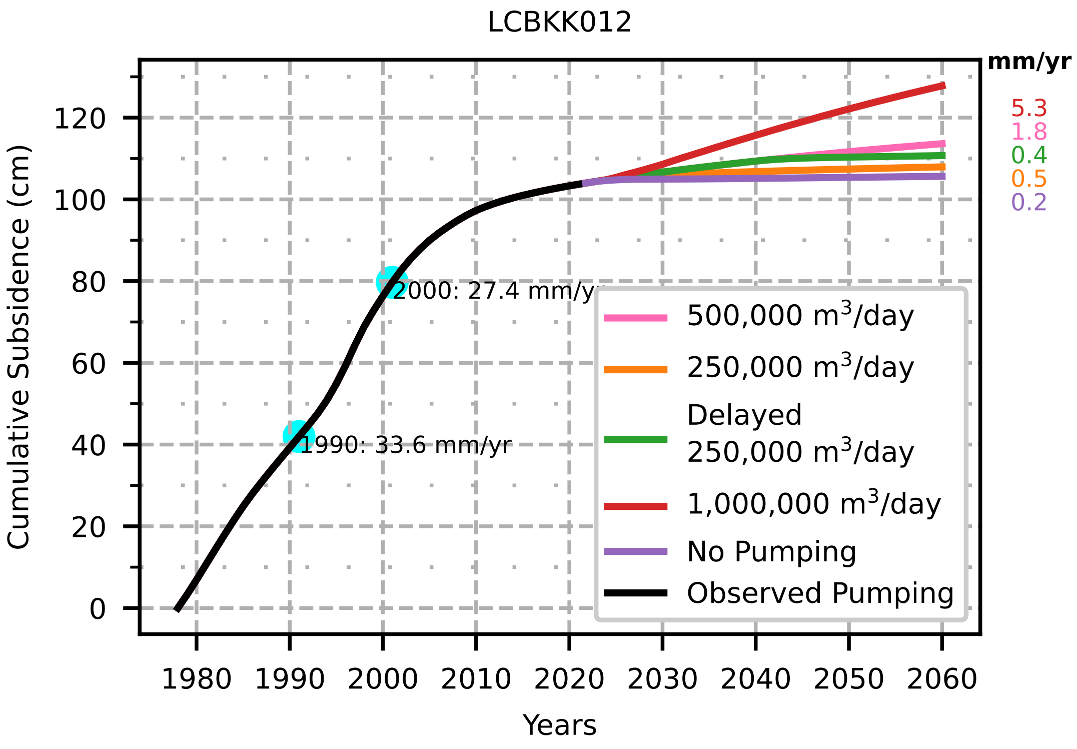

Figure 1.39: Simulated cumulative subsidence for 1978-2060 for well nest (LC)BKK012. For 2020-2060, cumulative subsidence is plotted for several pumping scenarios. The 2060 subsidence rate is shown in the upper right hand corner for each scenario.

1.5.8 BKK013

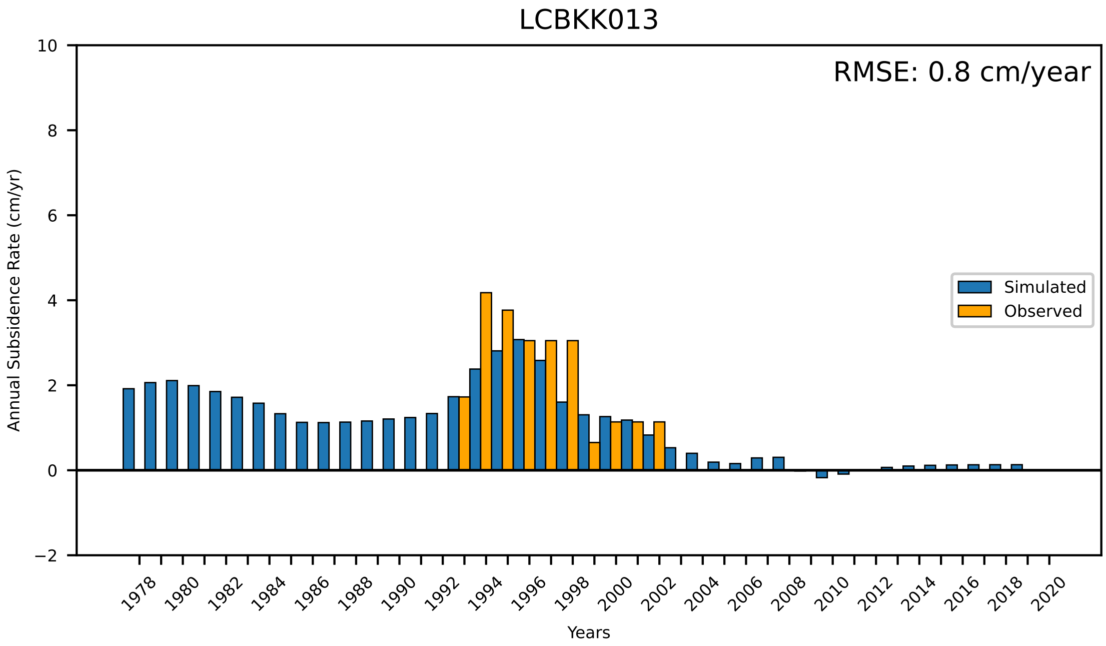

Figure 1.40: Simulated subsidence rates (blue) plotted against observed subsidence rates (orange) for well nest (LC)BKK013.

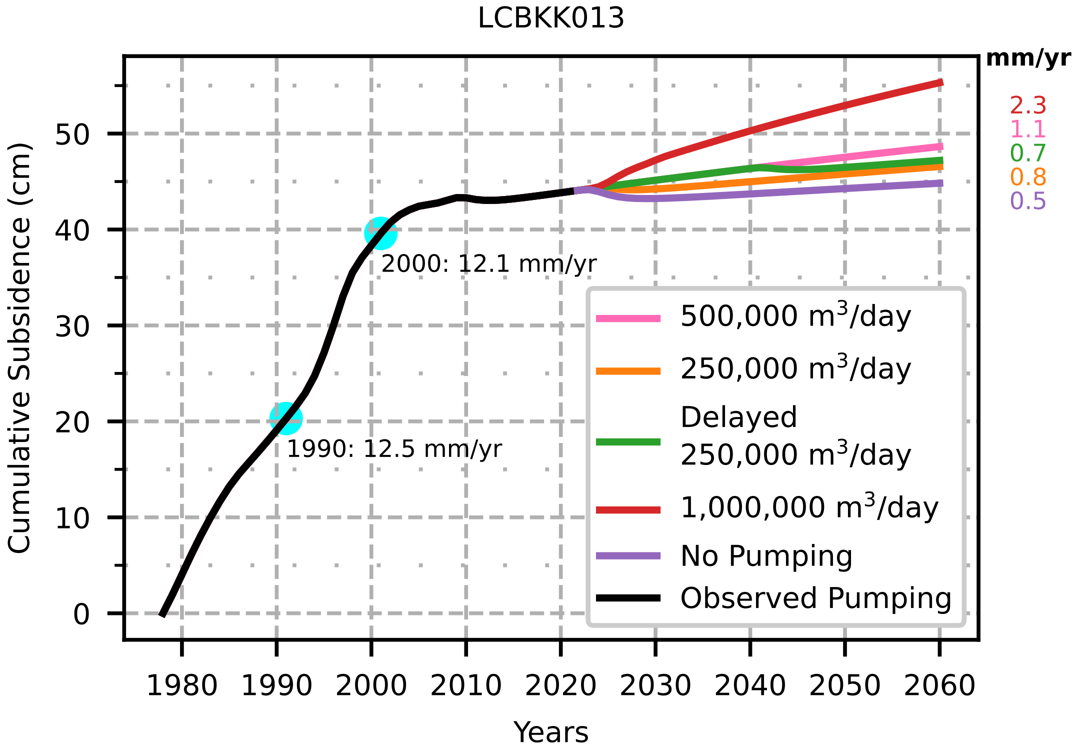

Figure 1.41: Simulated cumulative subsidence for 1978-2060 for well nest (LC)BKK013. For 2020-2060, cumulative subsidence is plotted for several pumping scenarios. The 2060 subsidence rate is shown in the upper right hand corner for each scenario.

1.5.9 BKK014

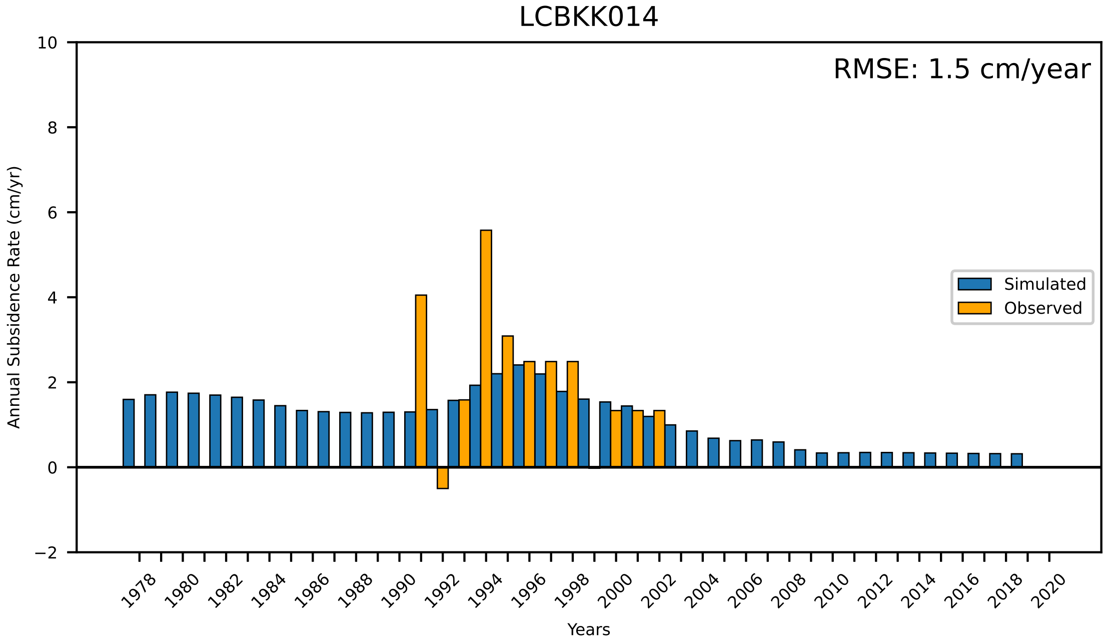

Figure 1.42: Simulated subsidence rates (blue) plotted against observed subsidence rates (orange) for well nest (LC)BKK014.

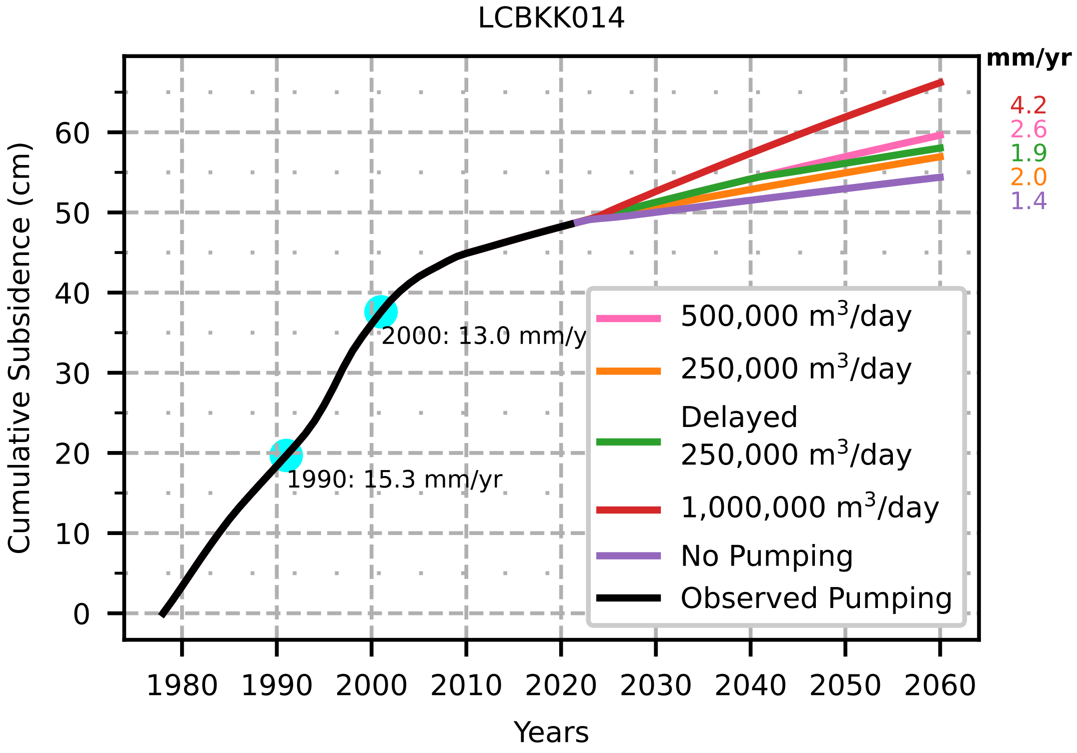

Figure 1.43: Simulated cumulative subsidence for 1978-2060 for well nest (LC)BKK014. For 2020-2060, cumulative subsidence is plotted for several pumping scenarios. The 2060 subsidence rate is shown in the upper right hand corner for each scenario.

1.5.10 BKK015

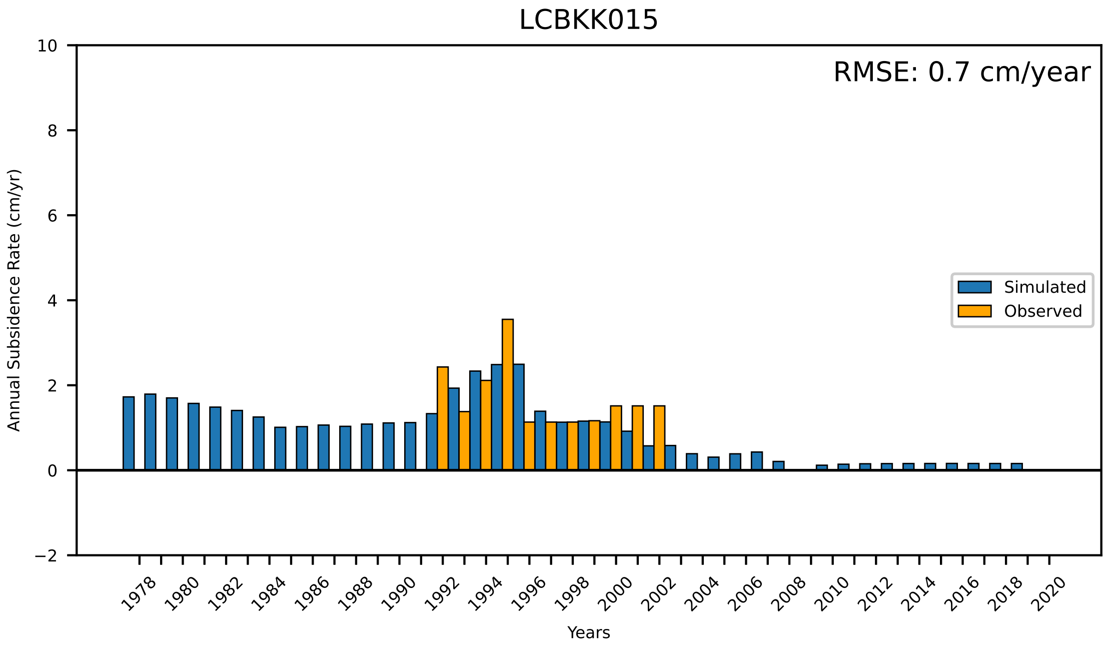

Figure 1.44: Simulated subsidence rates (blue) plotted against observed subsidence rates (orange) for well nest (LC)BKK015.

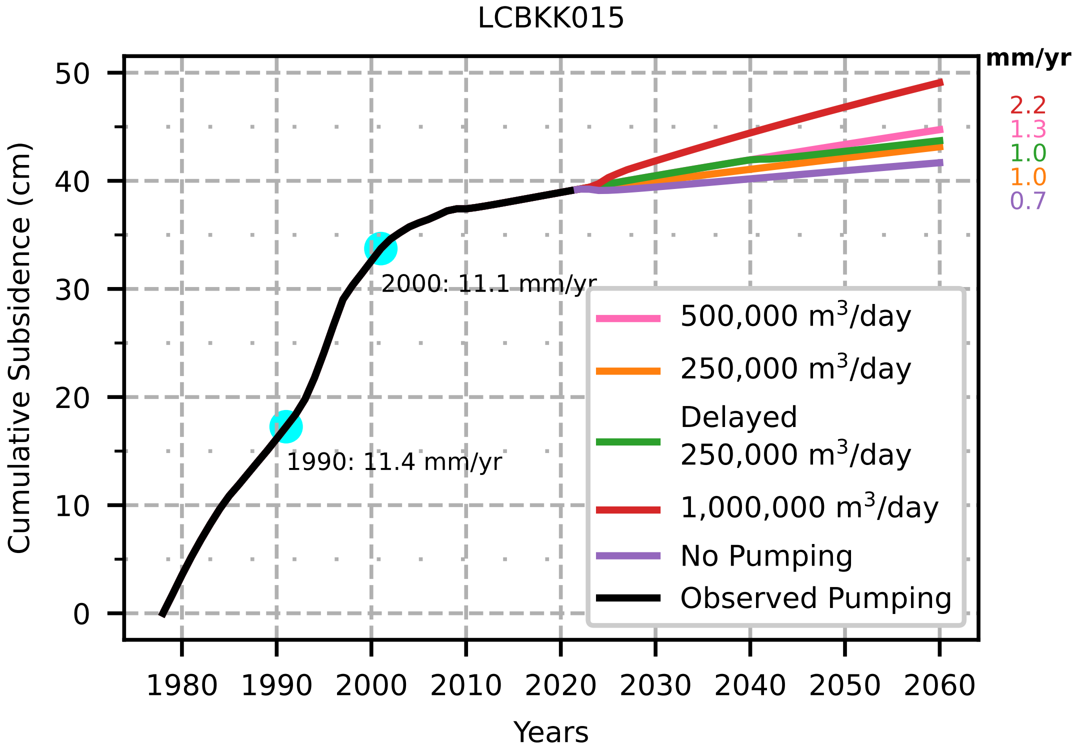

Figure 1.45: Simulated cumulative subsidence for 1978-2060 for well nest (LC)BKK015. For 2020-2060, cumulative subsidence is plotted for several pumping scenarios. The 2060 subsidence rate is shown in the upper right hand corner for each scenario.

1.5.11 BKK016

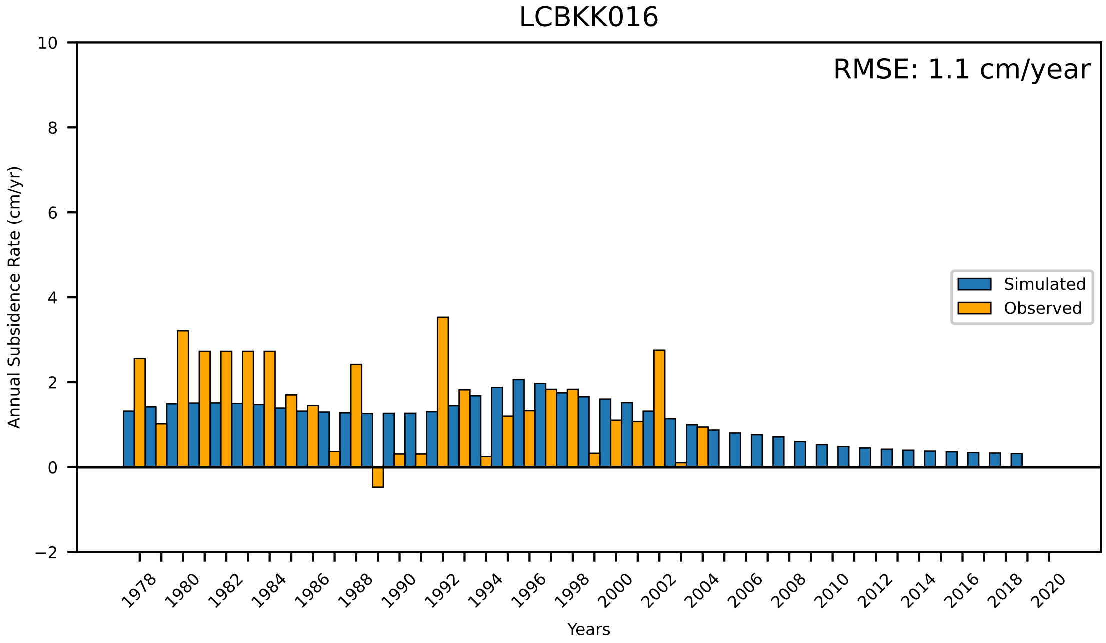

Figure 1.46: Simulated subsidence rates (blue) plotted against observed subsidence rates (orange) for well nest (LC)BKK016.

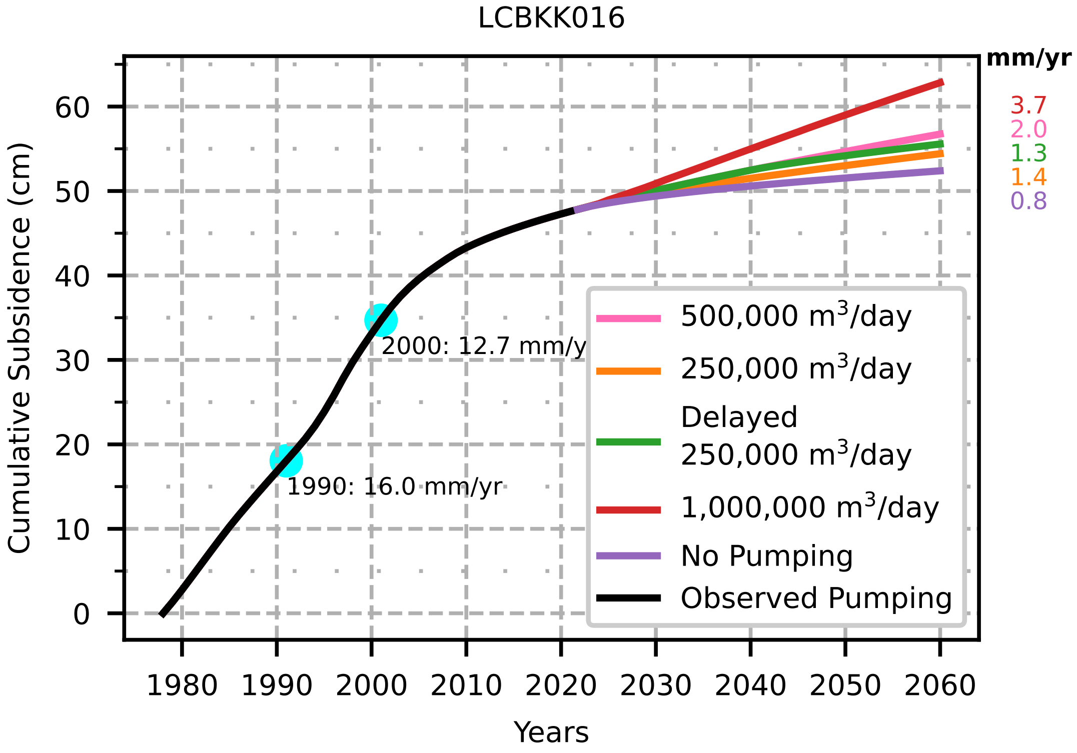

Figure 1.47: Simulated cumulative subsidence for 1978-2060 for well nest (LC)BKK016. For 2020-2060, cumulative subsidence is plotted for several pumping scenarios. The 2060 subsidence rate is shown in the upper right hand corner for each scenario.

1.5.12 BKK018

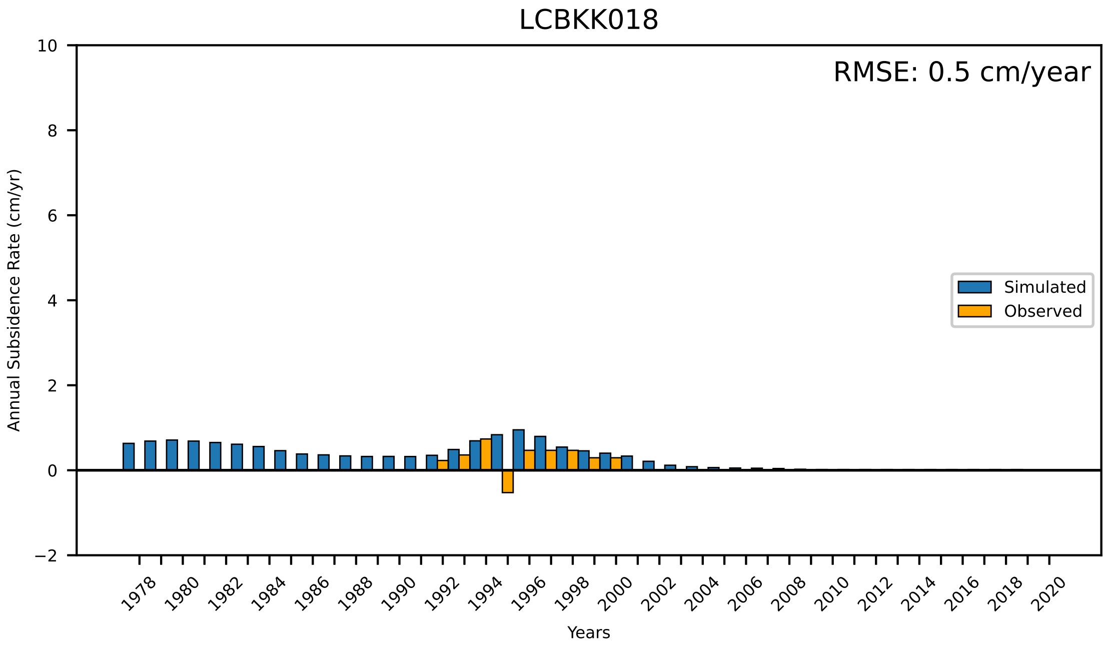

Figure 1.48: Simulated subsidence rates (blue) plotted against observed subsidence rates (orange) for well nest (LC)BKK018.

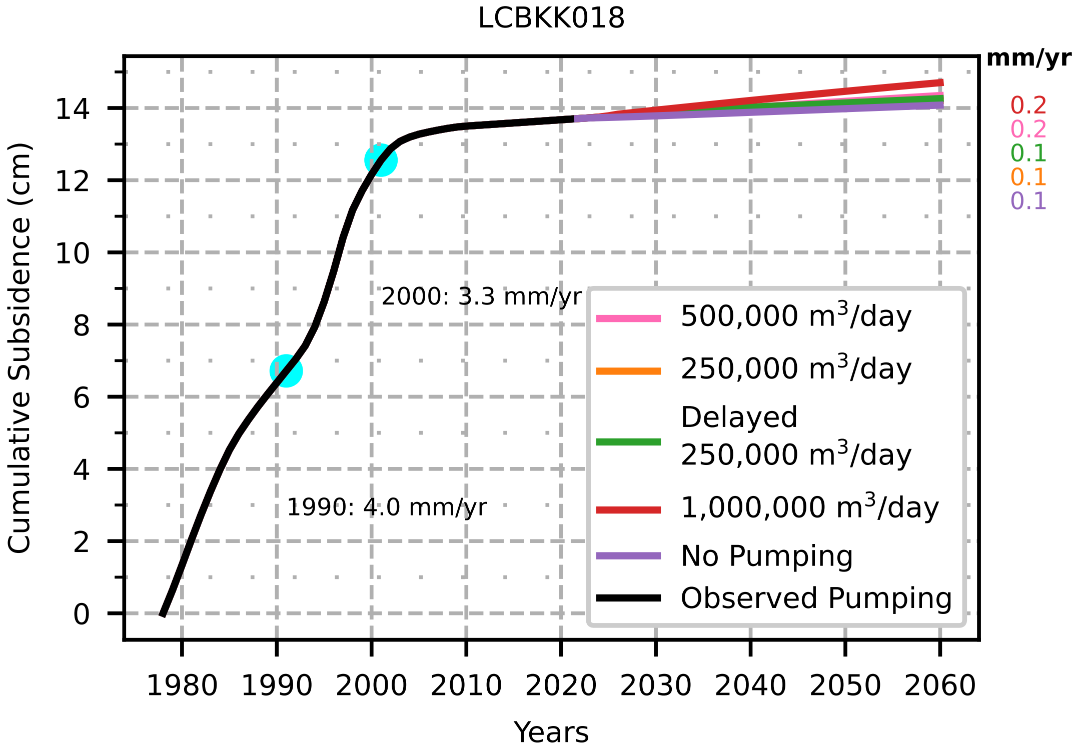

Figure 1.49: Simulated cumulative subsidence for 1978-2060 for well nest (LC)BKK018. For 2020-2060, cumulative subsidence is plotted for several pumping scenarios. The 2060 subsidence rate is shown in the upper right hand corner for each scenario.

1.5.13 BKK020

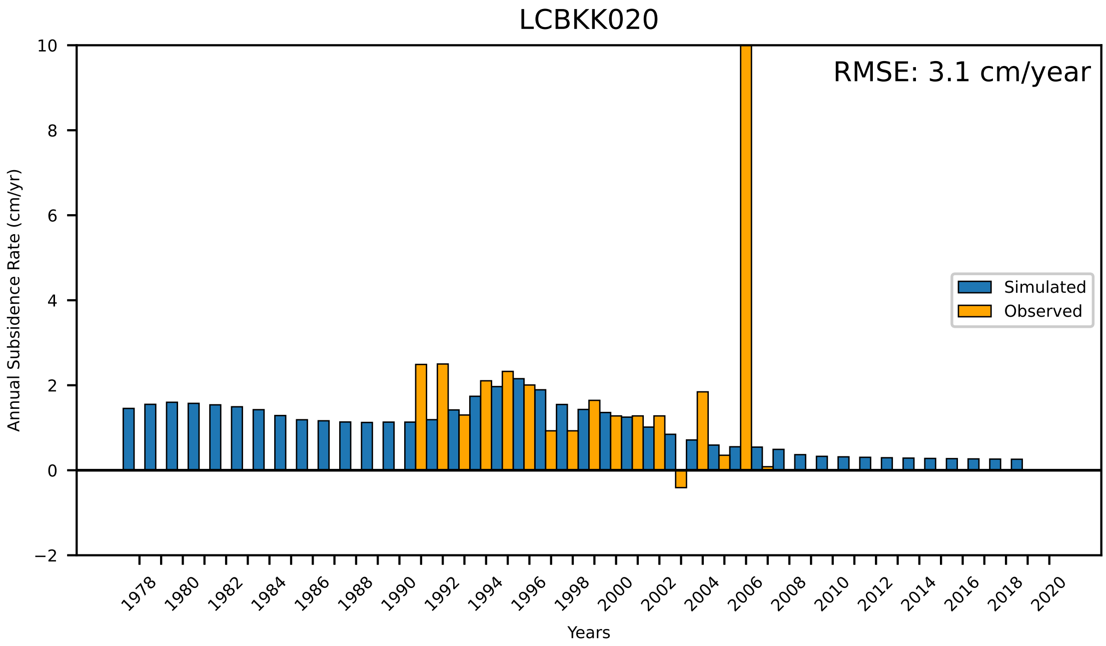

Figure 1.50: Simulated subsidence rates (blue) plotted against observed subsidence rates (orange) for well nest (LC)BKK020.

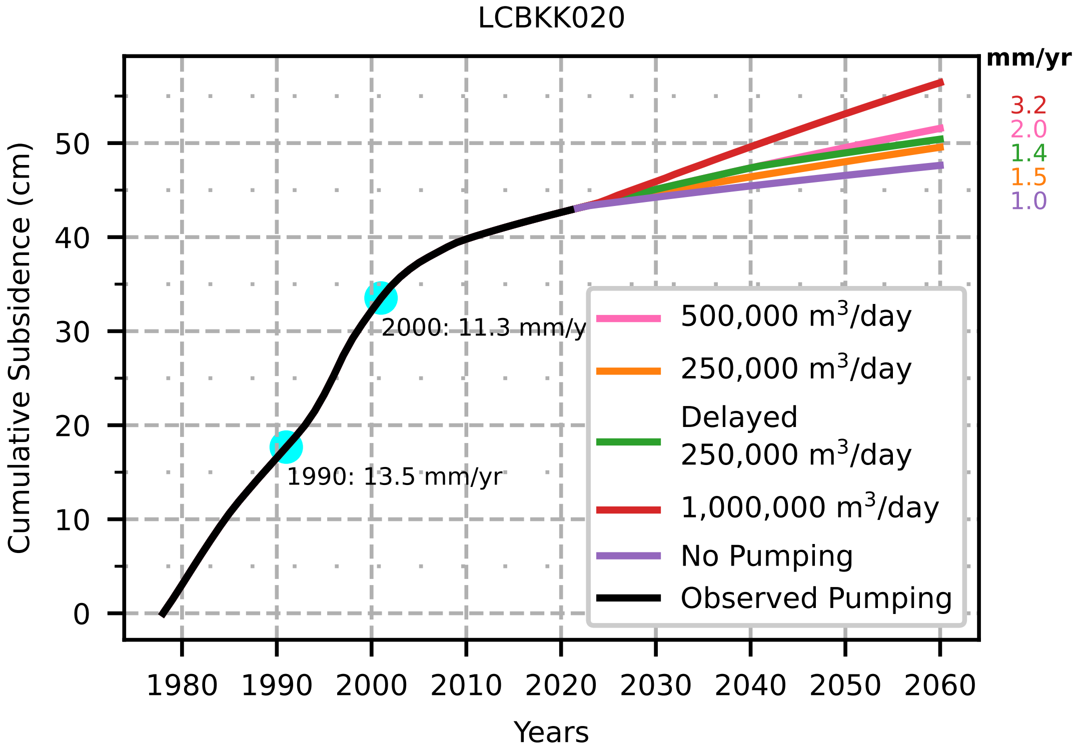

Figure 1.51: Simulated cumulative subsidence for 1978-2060 for well nest (LC)BKK020. For 2020-2060, cumulative subsidence is plotted for several pumping scenarios. The 2060 subsidence rate is shown in the upper right hand corner for each scenario.

1.5.14 BKK021

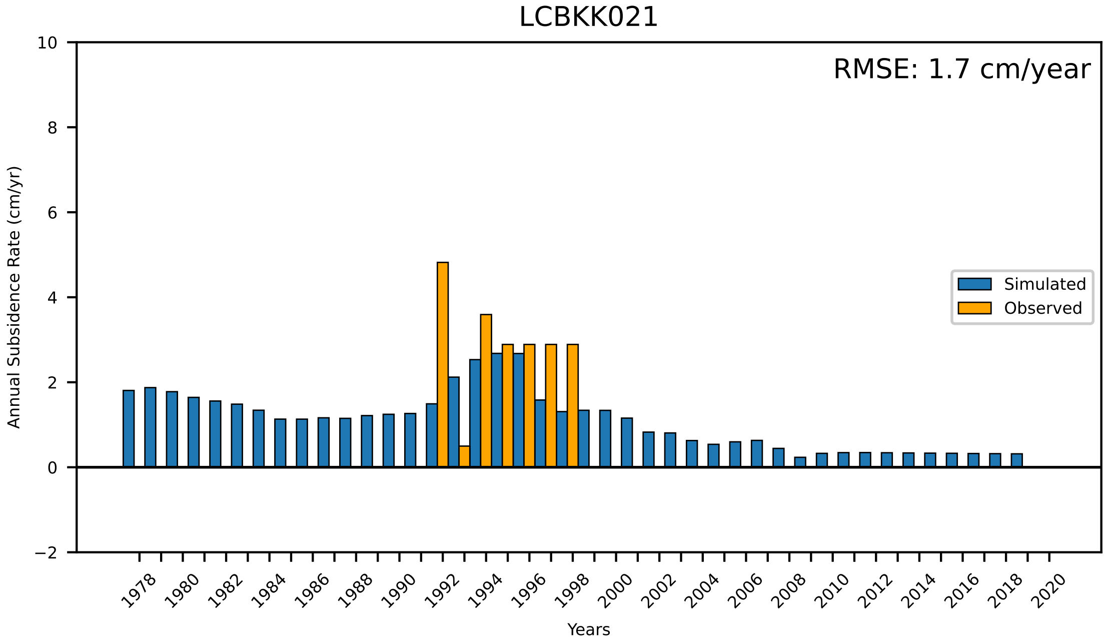

Figure 1.52: Simulated subsidence rates (blue) plotted against observed subsidence rates (orange) for well nest (LC)BKK021.

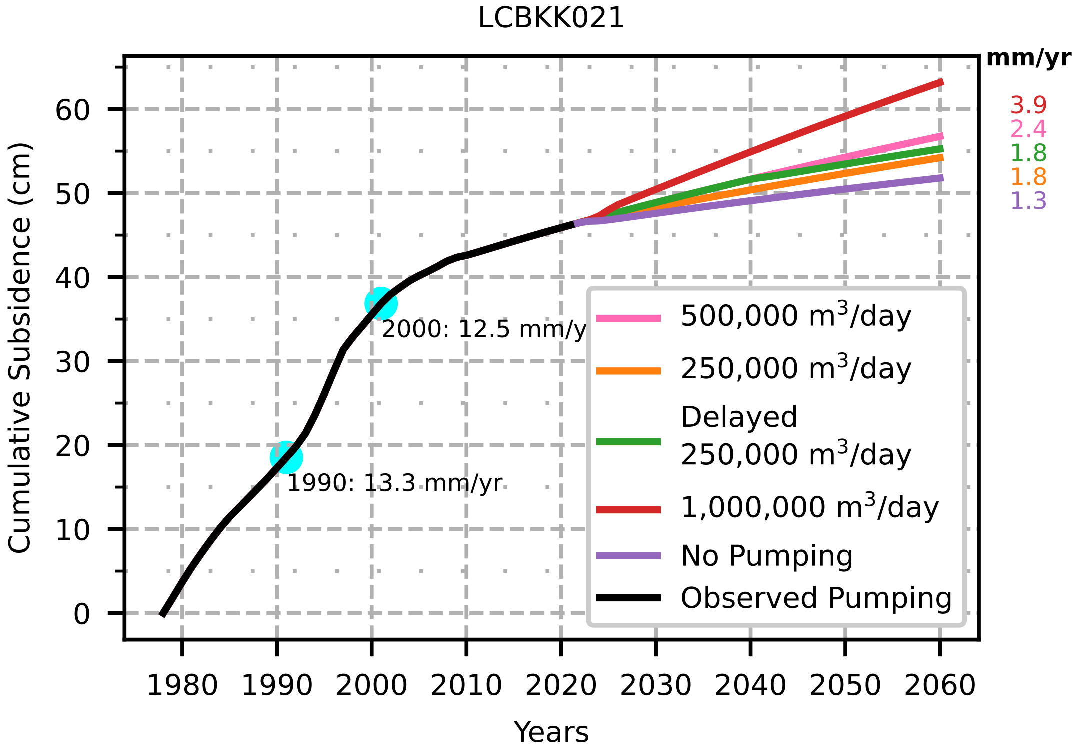

Figure 1.53: Simulated cumulative subsidence for 1978-2060 for well nest (LC)BKK021. For 2020-2060, cumulative subsidence is plotted for several pumping scenarios. The 2060 subsidence rate is shown in the upper right hand corner for each scenario.

1.5.15 BKK026

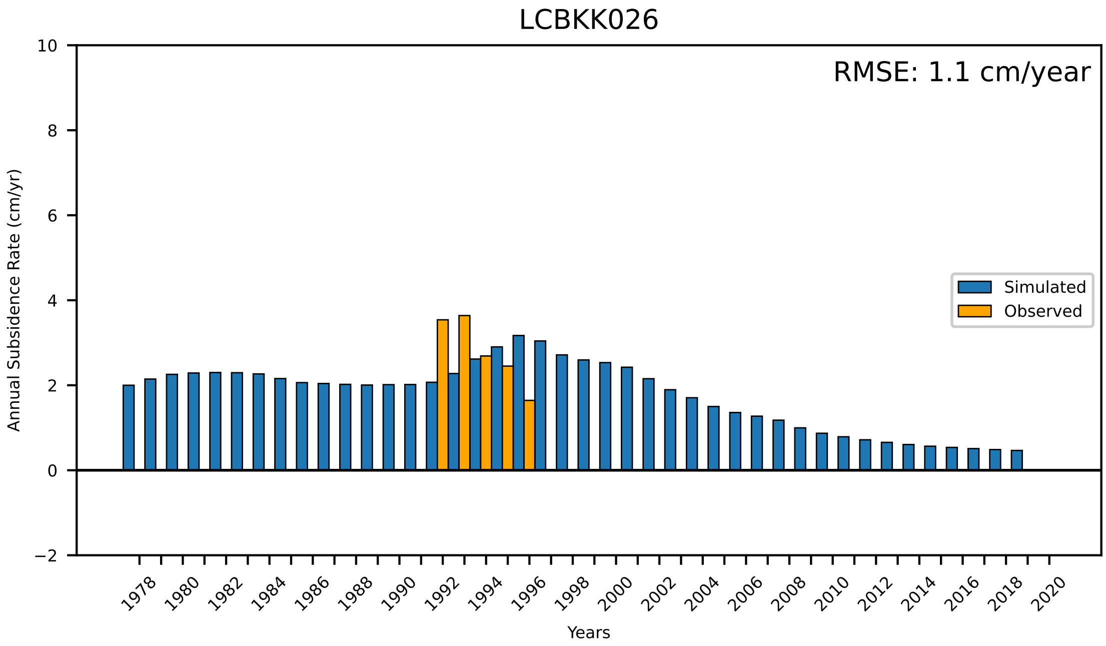

Figure 1.54: Simulated subsidence rates (blue) plotted against observed subsidence rates (orange) for well nest (LC)BKK026.

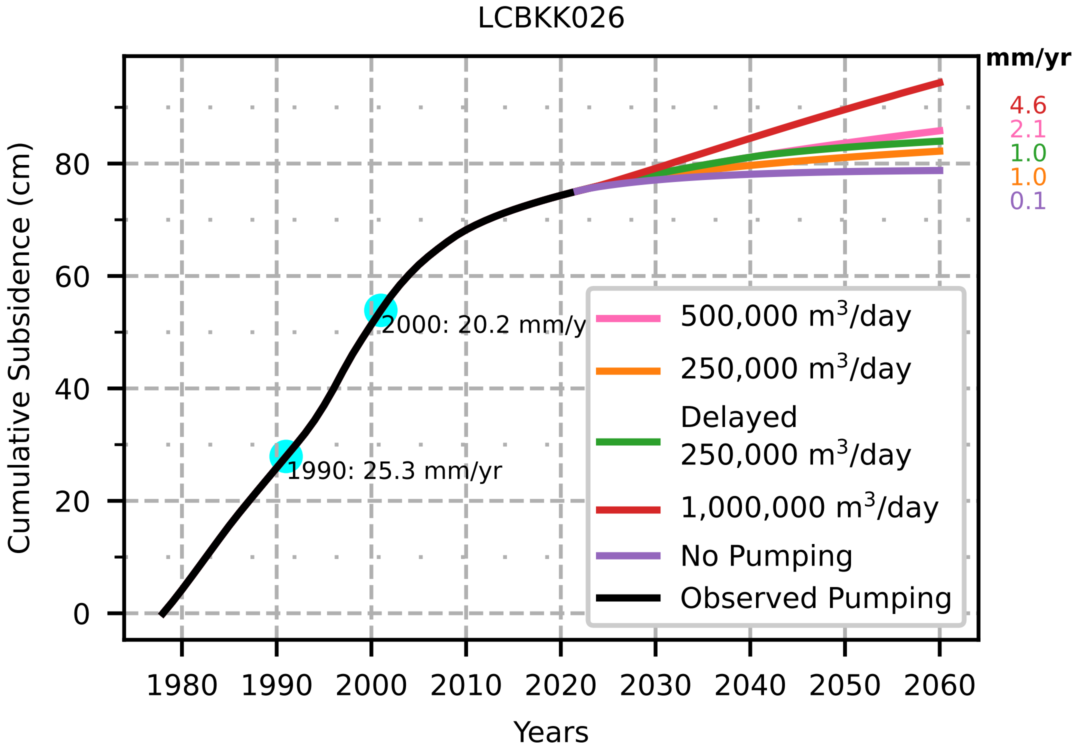

Figure 1.55: Simulated cumulative subsidence for 1978-2060 for well nest (LC)BKK026. For 2020-2060, cumulative subsidence is plotted for several pumping scenarios. The 2060 subsidence rate is shown in the upper right hand corner for each scenario.

1.5.16 BKK027

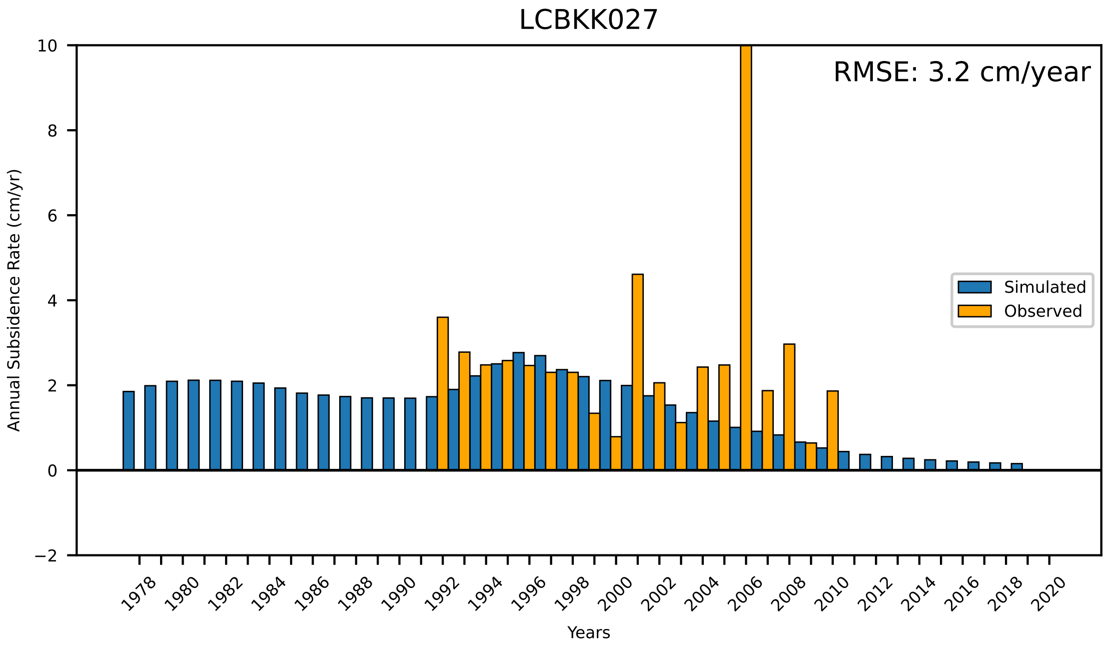

Figure 1.56: Simulated subsidence rates (blue) plotted against observed subsidence rates (orange) for well nest (LC)BKK027.

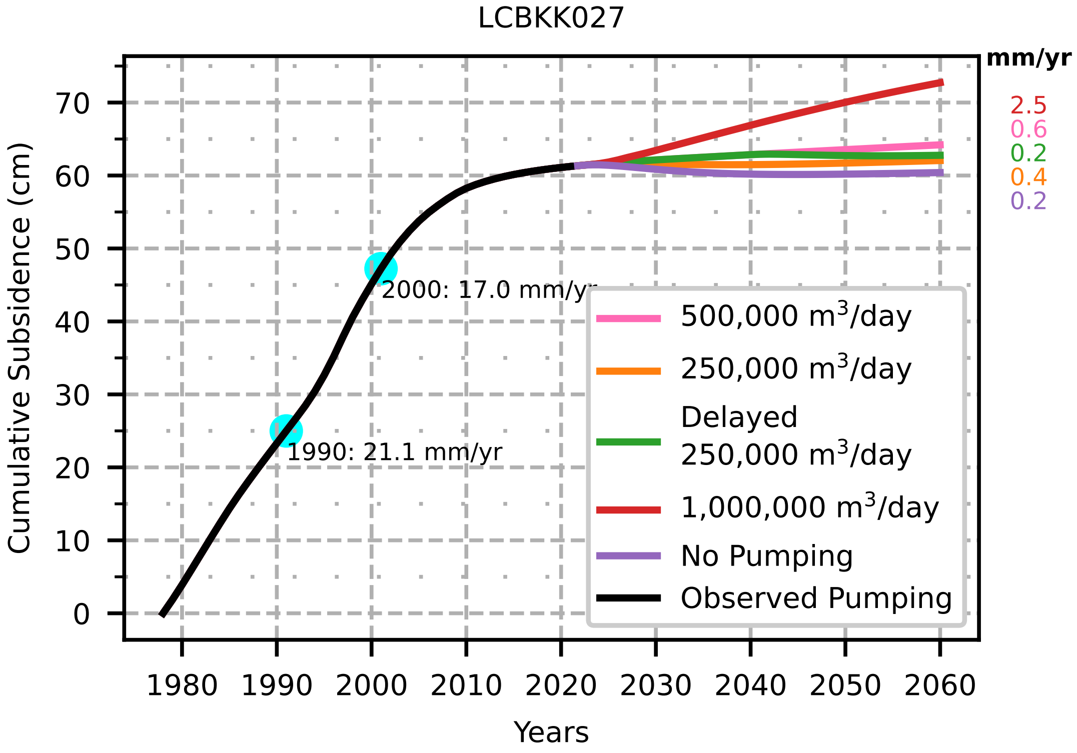

Figure 1.57: Simulated cumulative subsidence for 1978-2060 for well nest (LC)BKK027. For 2020-2060, cumulative subsidence is plotted for several pumping scenarios. The 2060 subsidence rate is shown in the upper right hand corner for each scenario.

1.5.17 BKK036

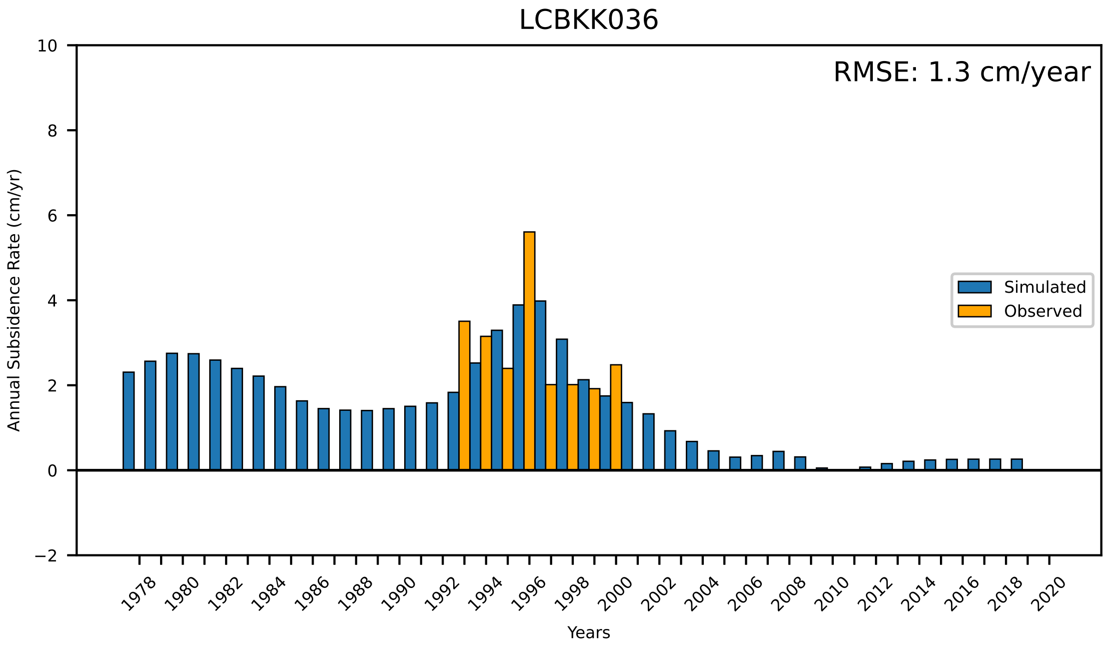

Figure 1.58: Simulated subsidence rates (blue) plotted against observed subsidence rates (orange) for well nest (LC)BKK036.

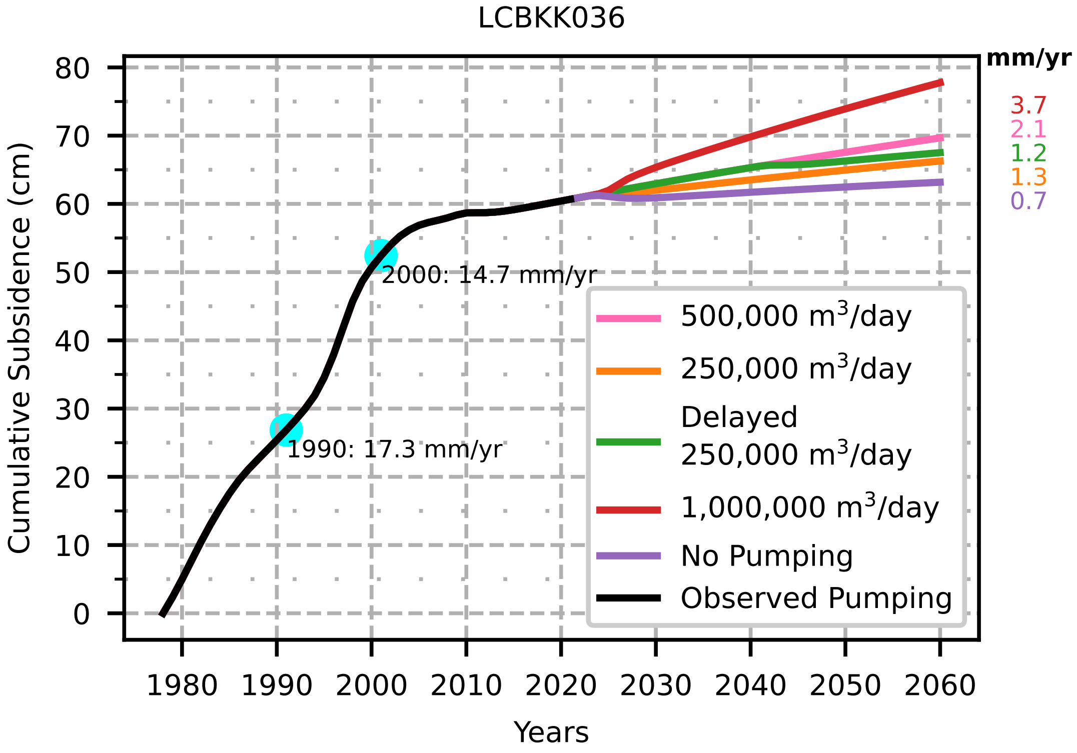

Figure 1.59: Simulated cumulative subsidence for 1978-2060 for well nest (LC)BKK036. For 2020-2060, cumulative subsidence is plotted for several pumping scenarios. The 2060 subsidence rate is shown in the upper right hand corner for each scenario.

1.5.18 BKK038

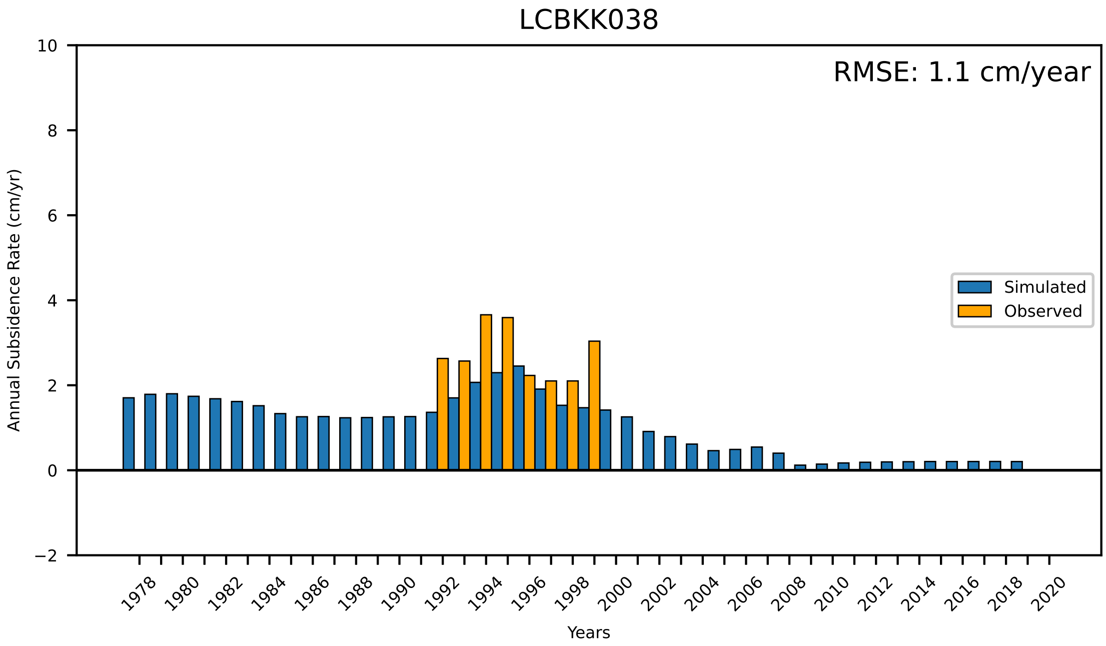

Figure 1.60: Simulated subsidence rates (blue) plotted against observed subsidence rates (orange) for well nest (LC)BKK038.

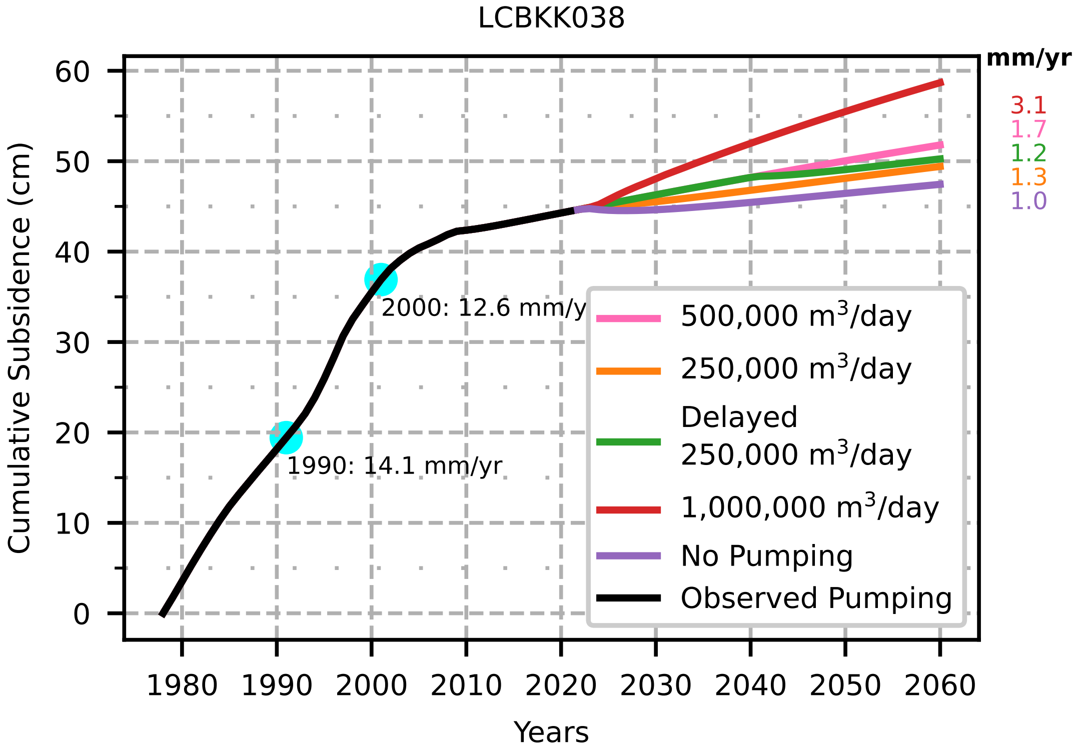

Figure 1.61: Simulated cumulative subsidence for 1978-2060 for well nest (LC)BKK038. For 2020-2060, cumulative subsidence is plotted for several pumping scenarios. The 2060 subsidence rate is shown in the upper right hand corner for each scenario.

1.5.19 BKK041

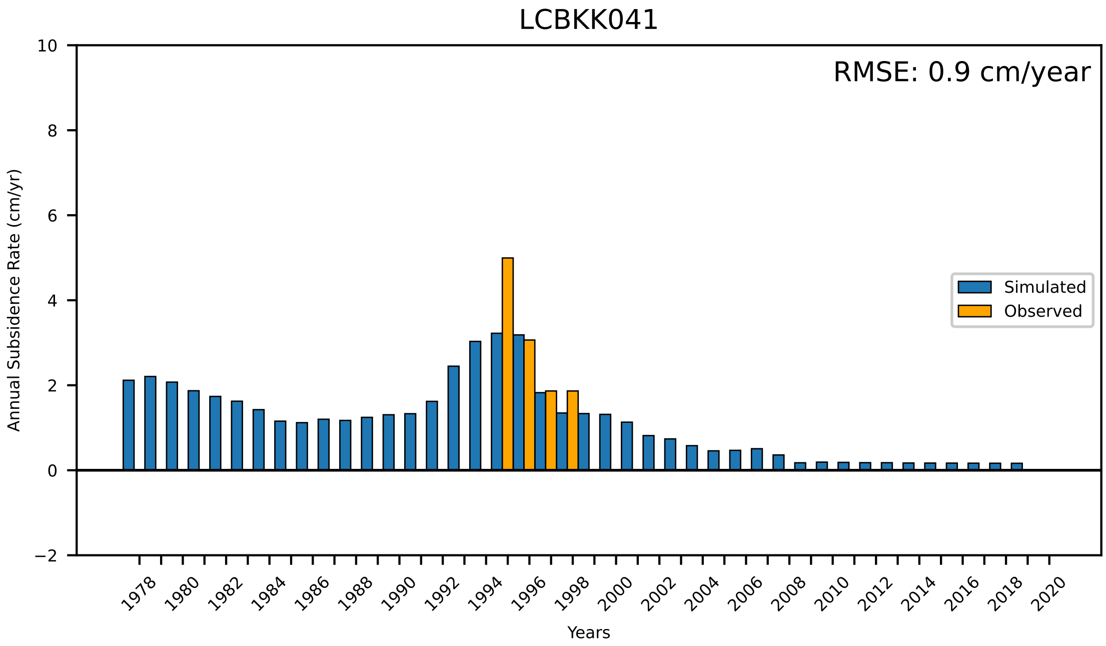

Figure 1.62: Simulated subsidence rates (blue) plotted against observed subsidence rates (orange) for well nest (LC)BKK041.

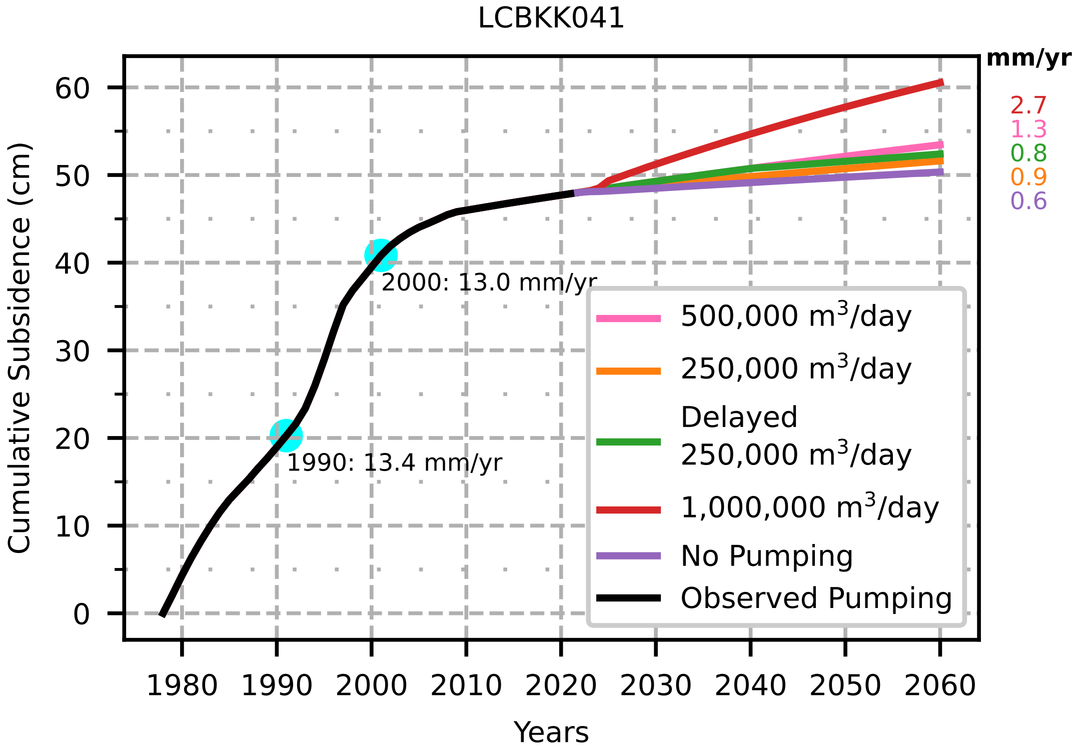

Figure 1.63: Simulated cumulative subsidence for 1978-2060 for well nest (LC)BKK041. For 2020-2060, cumulative subsidence is plotted for several pumping scenarios. The 2060 subsidence rate is shown in the upper right hand corner for each scenario.

1.5.20 NBI003

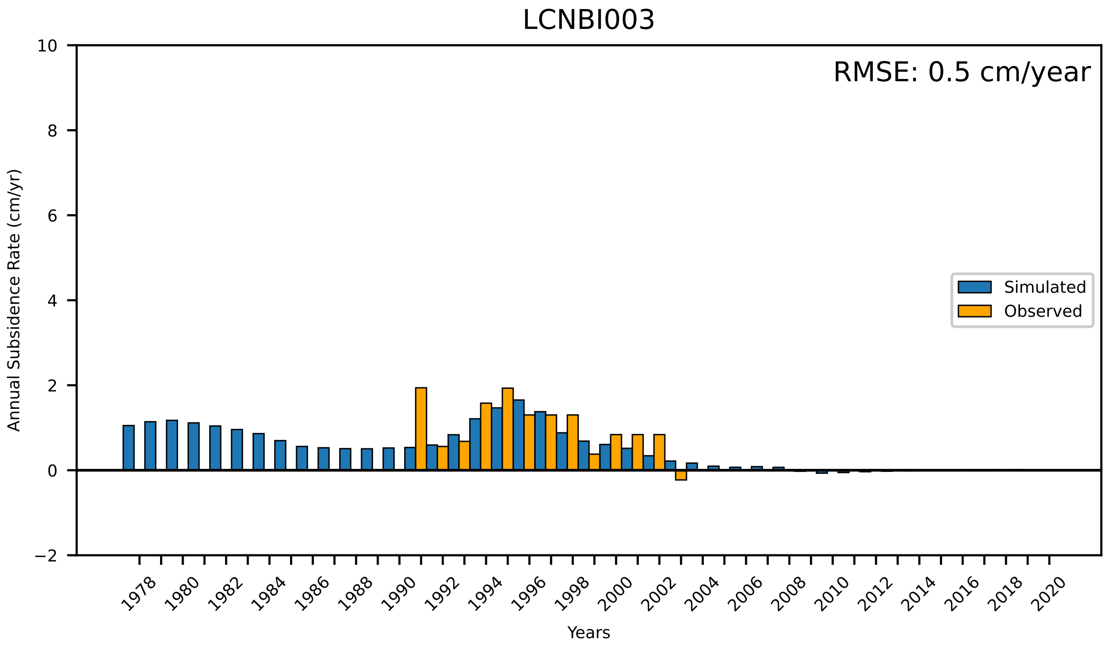

Figure 1.64: Simulated subsidence rates (blue) plotted against observed subsidence rates (orange) for well nest (LC)NBI003.

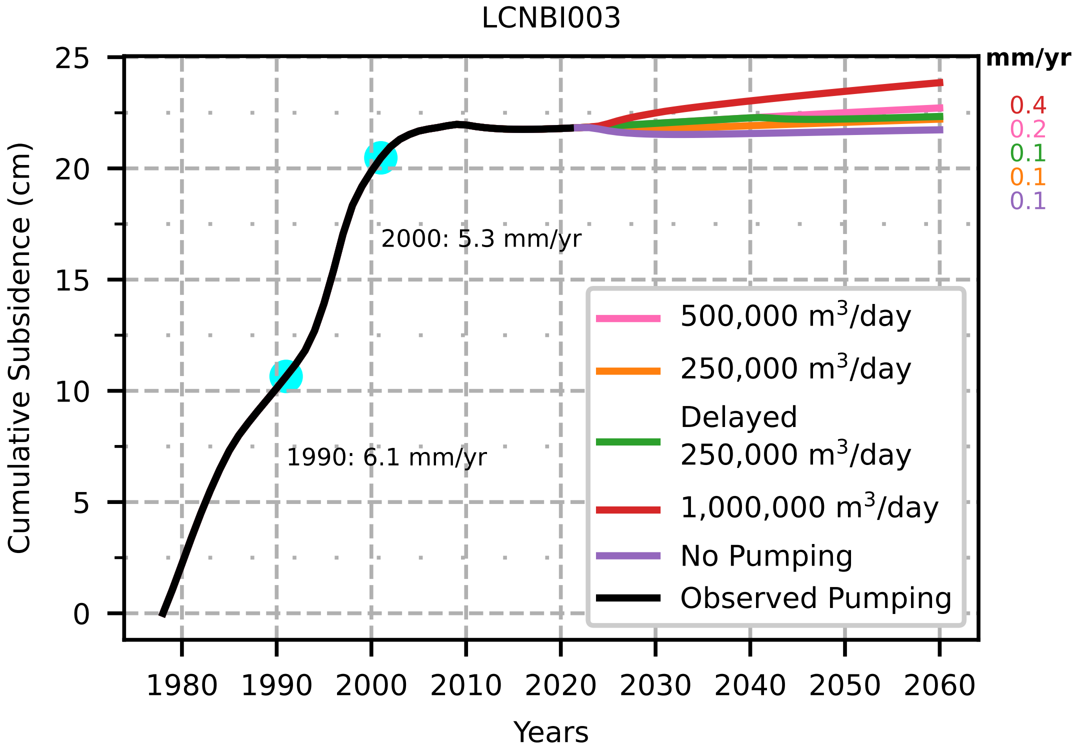

Figure 1.65: Simulated cumulative subsidence for 1978-2060 for well nest (LC)NBI003. For 2020-2060, cumulative subsidence is plotted for several pumping scenarios. The 2060 subsidence rate is shown in the upper right hand corner for each scenario.

1.5.21 NBI007

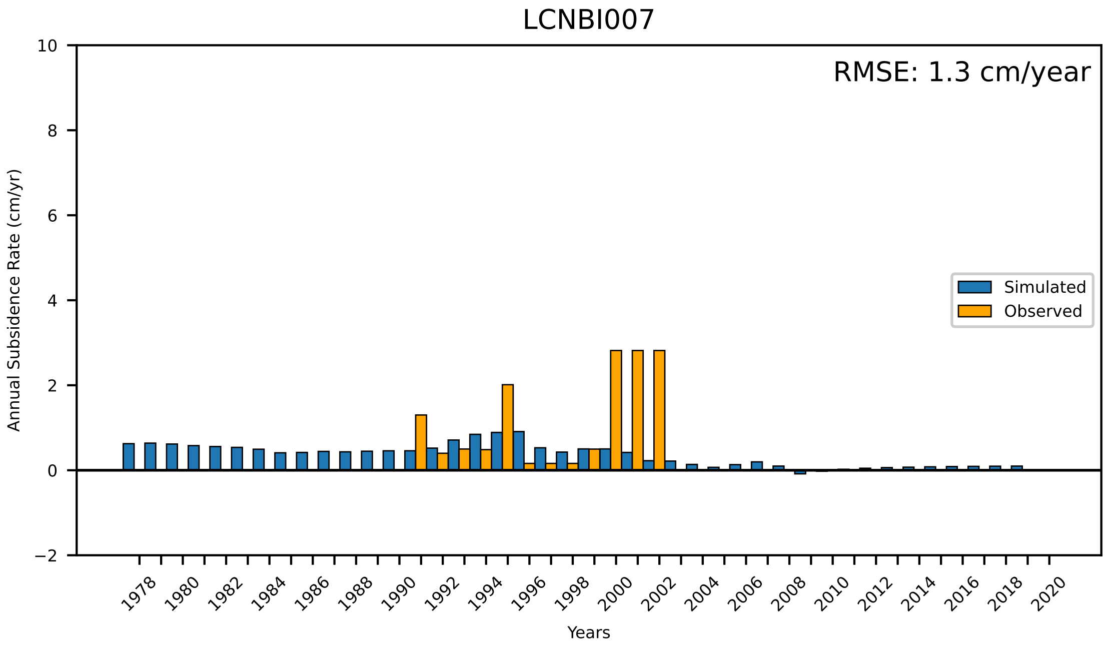

Figure 1.66: Simulated subsidence rates (blue) plotted against observed subsidence rates (orange) for well nest (LC)NBI007.

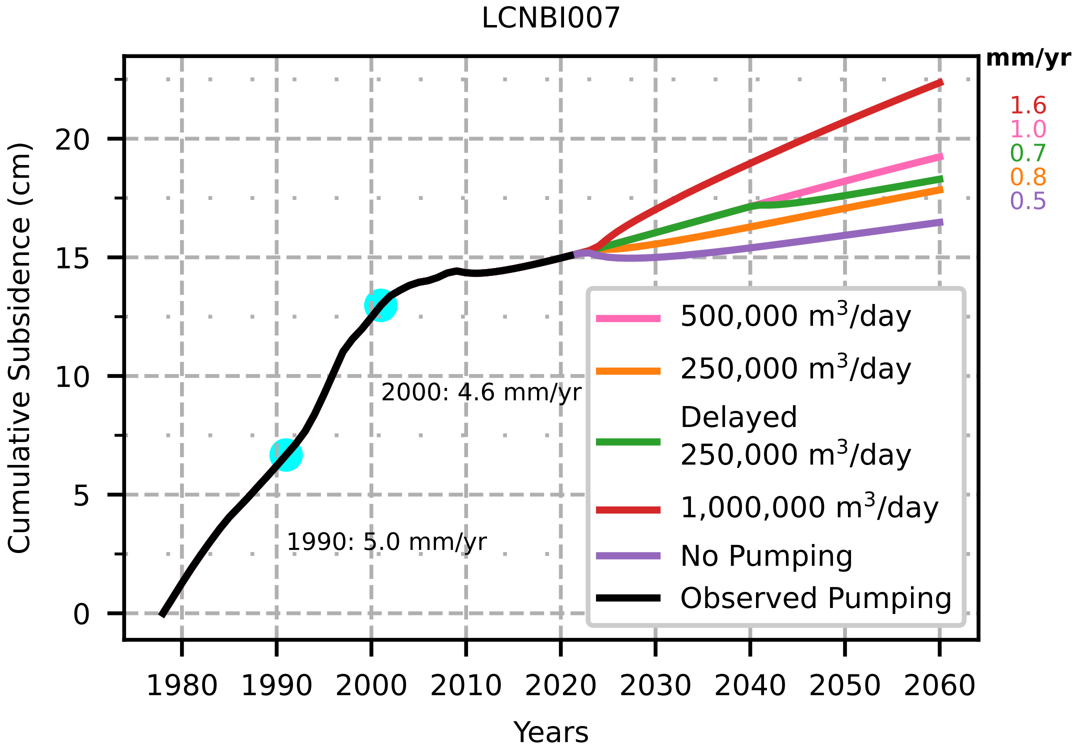

Figure 1.67: Simulated cumulative subsidence for 1978-2060 for well nest (LC)NBI007. For 2020-2060, cumulative subsidence is plotted for several pumping scenarios. The 2060 subsidence rate is shown in the upper right hand corner for each scenario.

1.5.22 SPK007

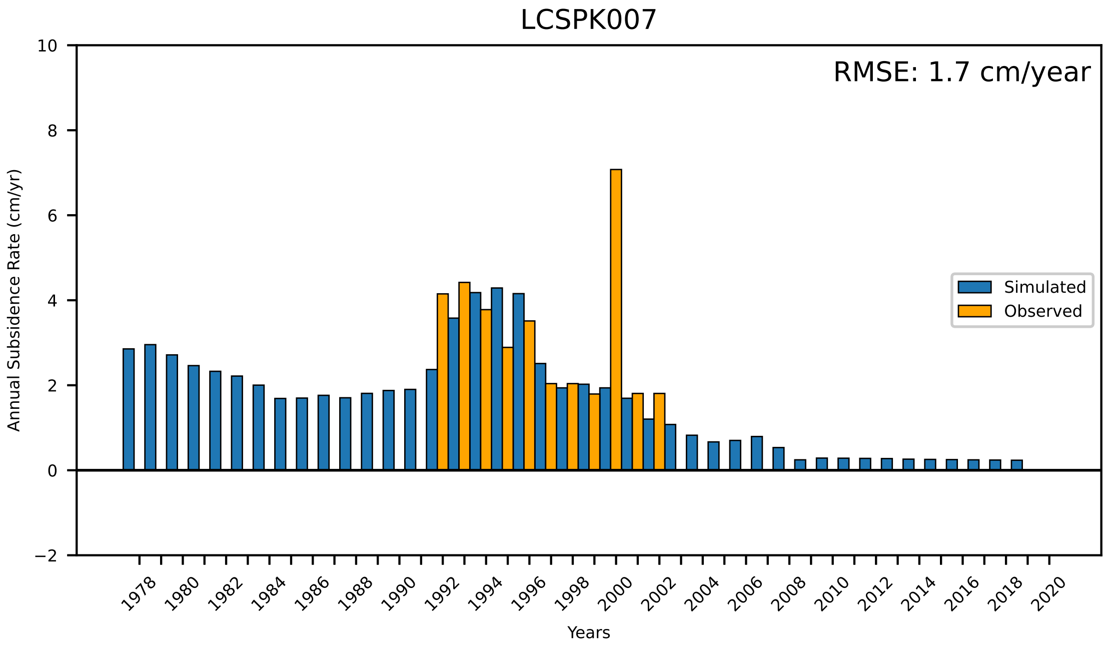

Figure 1.68: Simulated subsidence rates (blue) plotted against observed subsidence rates (orange) for well nest (LC)SPK007.

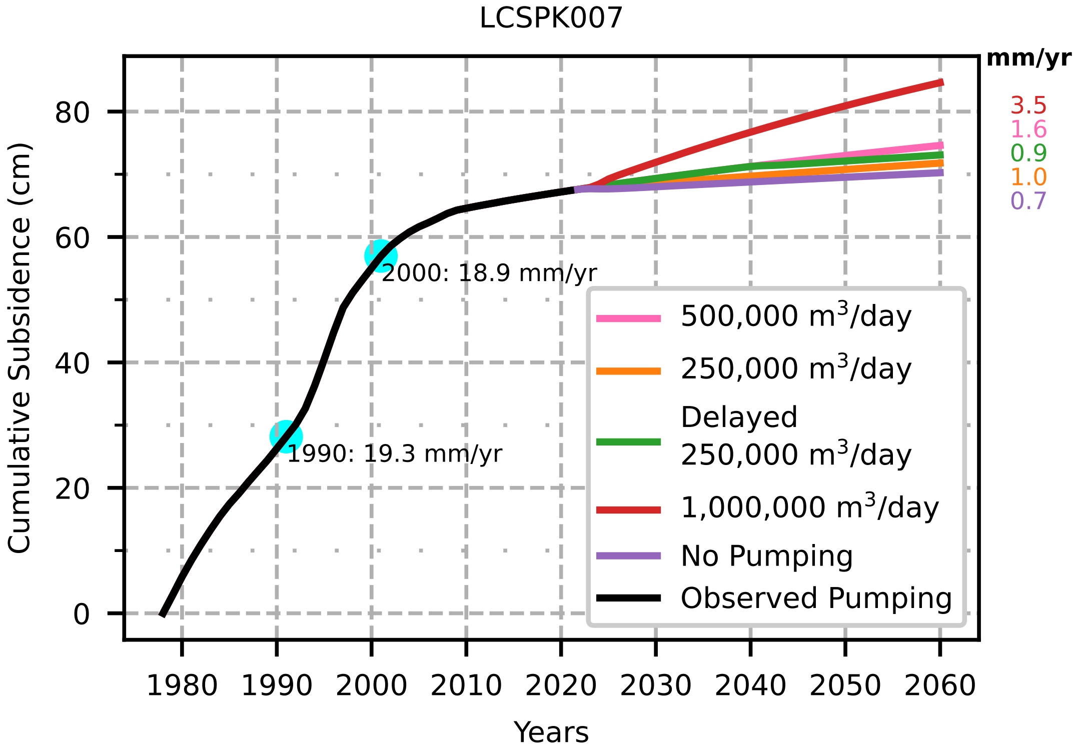

Figure 1.69: Simulated cumulative subsidence for 1978-2060 for well nest (LC)SPK007. For 2020-2060, cumulative subsidence is plotted for several pumping scenarios. The 2060 subsidence rate is shown in the upper right hand corner for each scenario.

1.5.23 SPK009

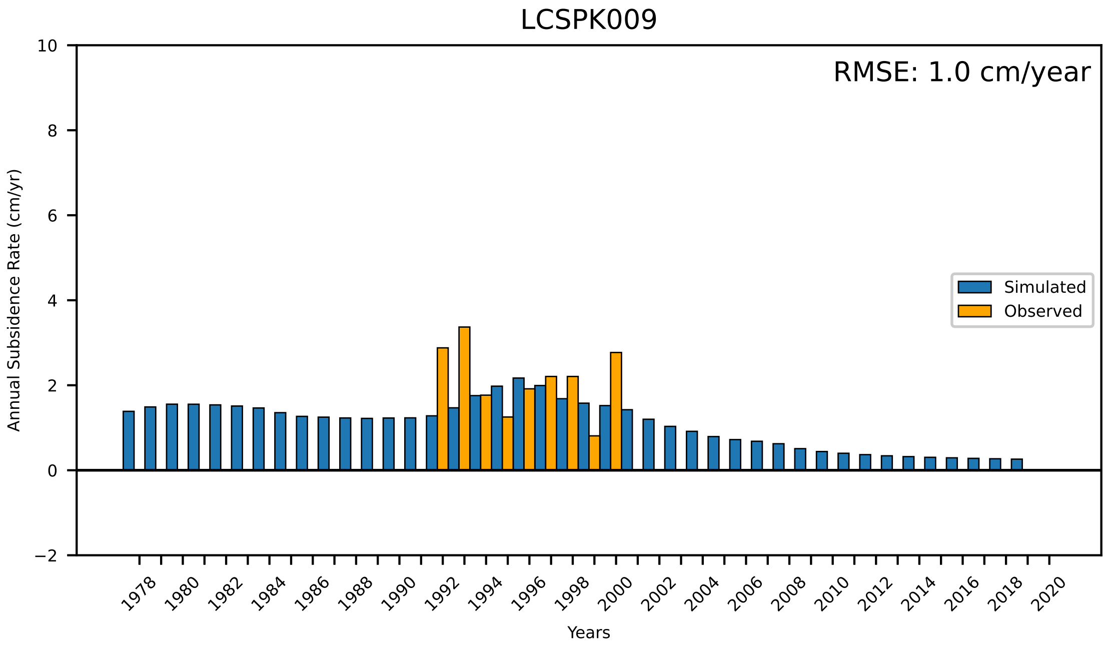

Figure 1.70: Simulated subsidence rates (blue) plotted against observed subsidence rates (orange) for well nest (LC)SPK009.

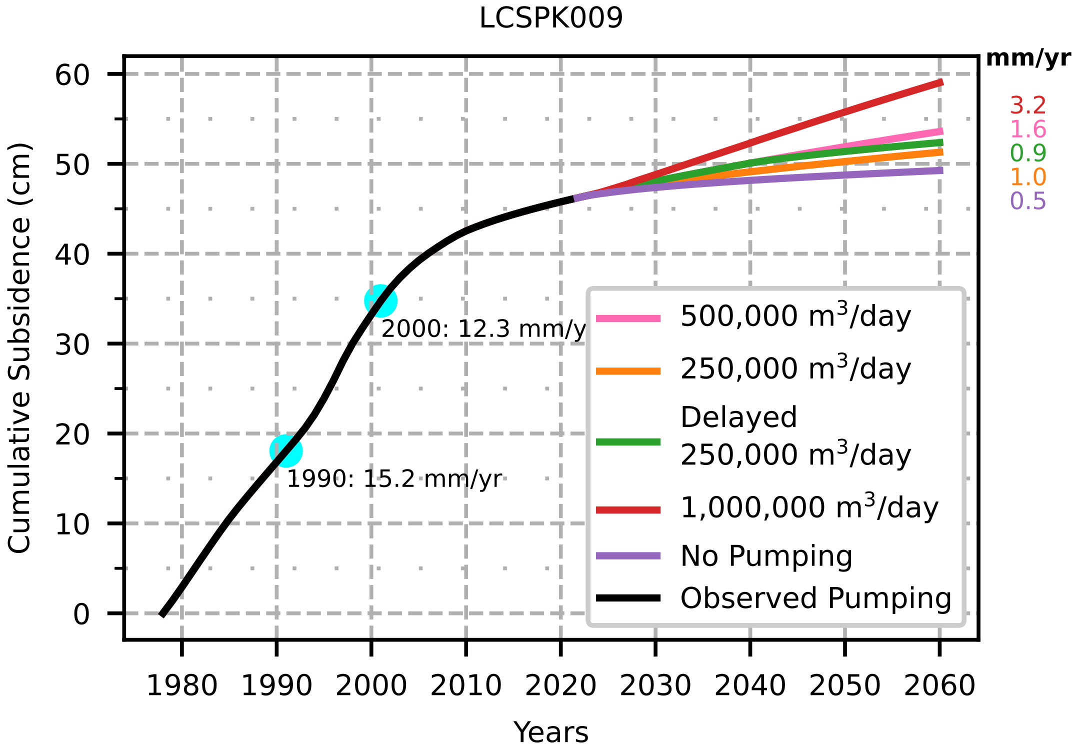

Figure 1.71: Simulated cumulative subsidence for 1978-2060 for well nest (LC)SPK009. For 2020-2060, cumulative subsidence is plotted for several pumping scenarios. The 2060 subsidence rate is shown in the upper right hand corner for each scenario.

1.6 Sensitivity Analyses

1.6.1 Groundwater Levels

Pumping Sensitivity

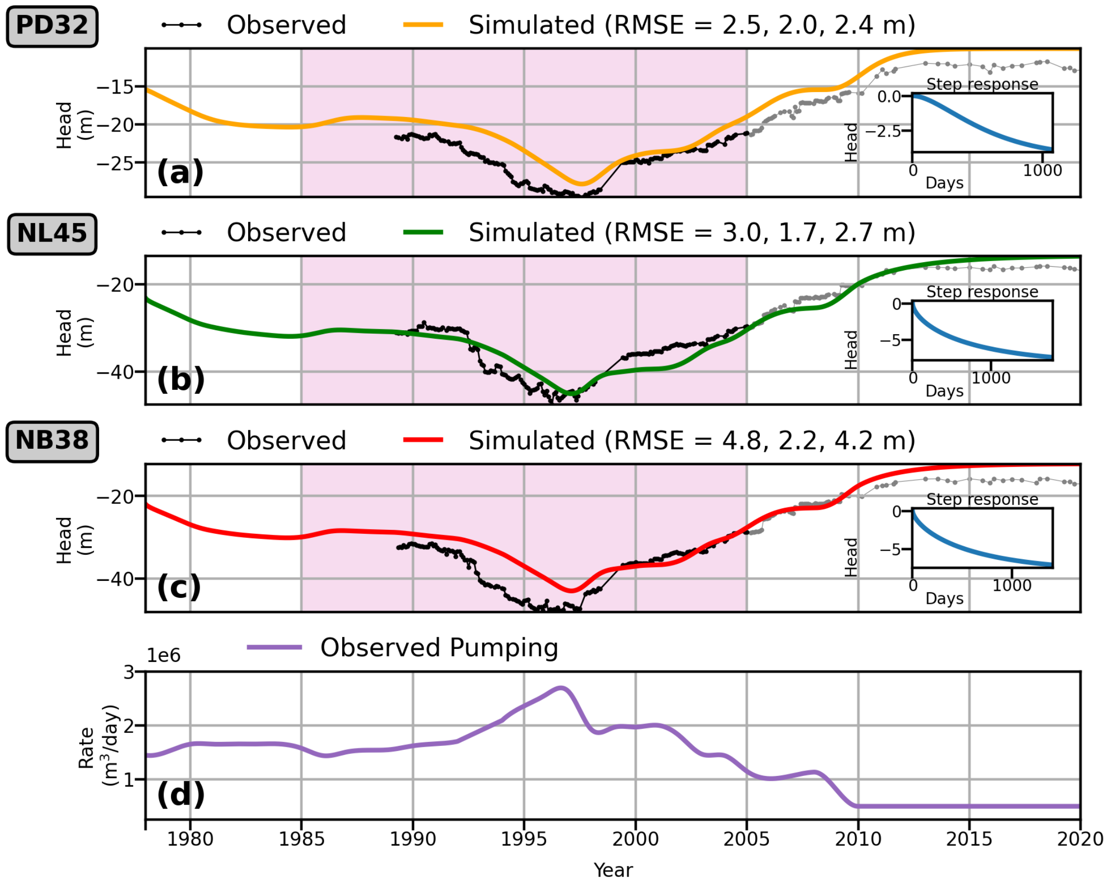

Figure 1.72: Results for Pastas time series models for well PD32 in well nest (LC)BKK013 with 15% increased pumping rate for 1954-1993.

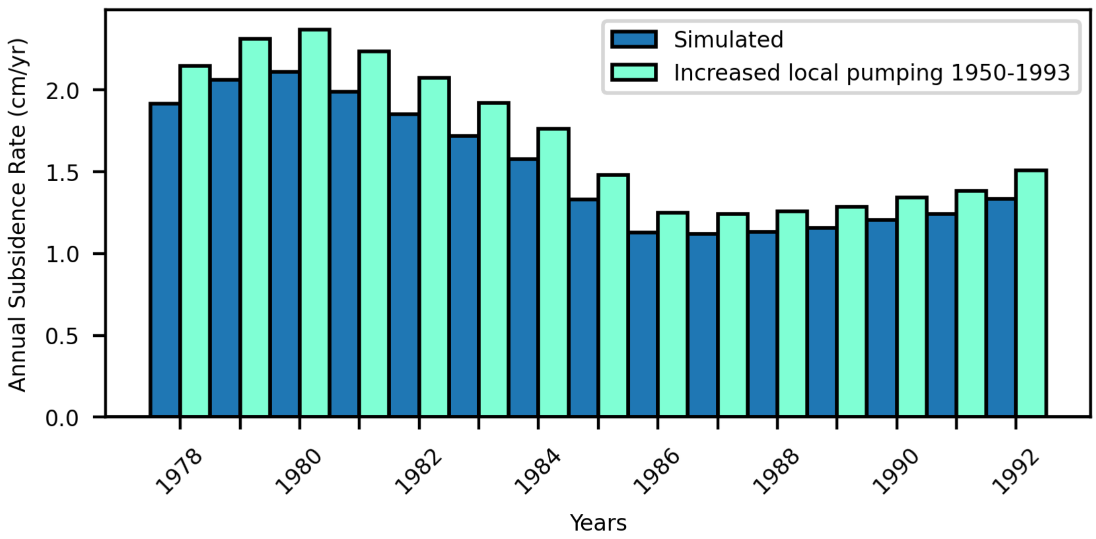

Figure 1.73: Annual subsidence rate results when pumping is increased by 15% for 1954-1993 (aquamarine) vs. the original pumping rates (blue).

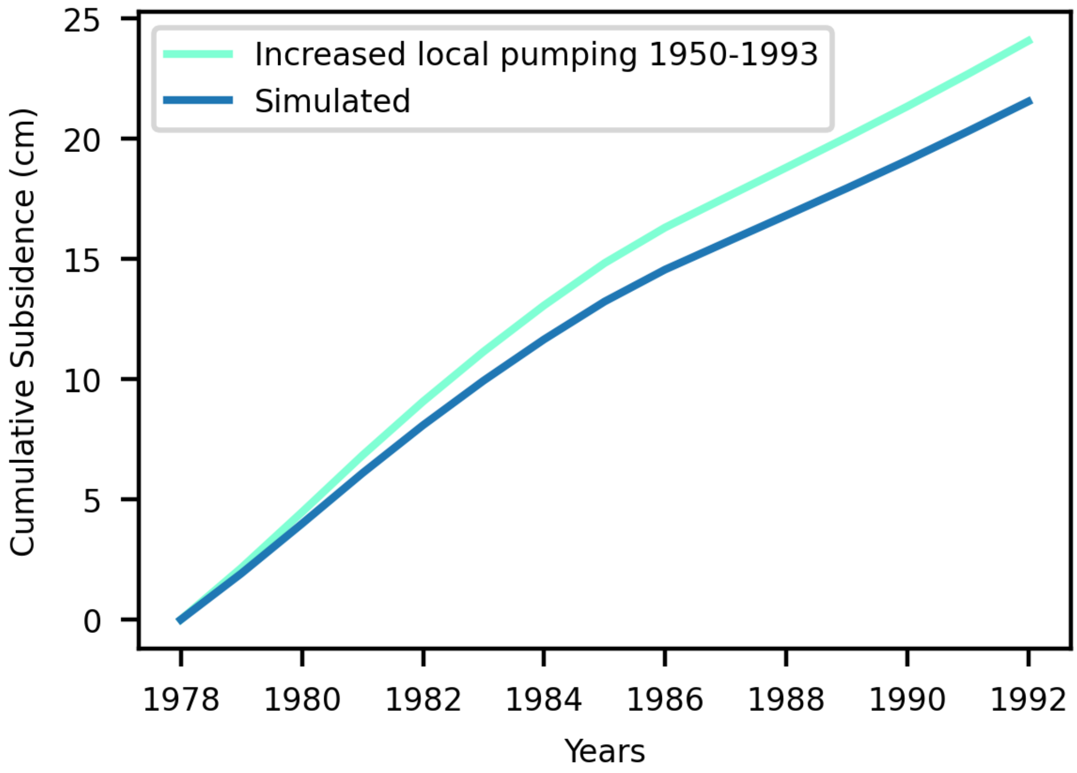

Figure 1.74: Cumulative subsidence results when pumping is increased by 15% for 1954-1993 (aquamarine) vs. the original pumping rates (blue). In 1993, there is an increase of 3 cm or 12% with the increase in local pumping.

1.6.2 Subsidence Parameters

BKK013

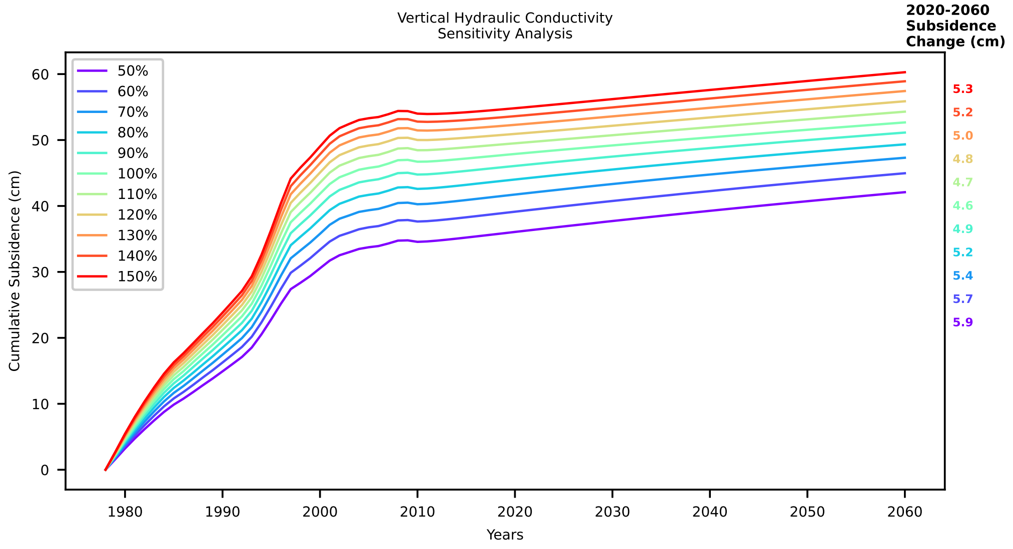

Figure 1.75: Simulated cumulative subsidence for 1978-2060 for well nest (LC)BKK013 after varying the vertical hydraulic conductivity of clay. For 2020-2060, cumulative subsidence is plotted for several pumping scenarios. The 2020-2060 subsidence change is shown in the upper right hand corner for each sensitivity.

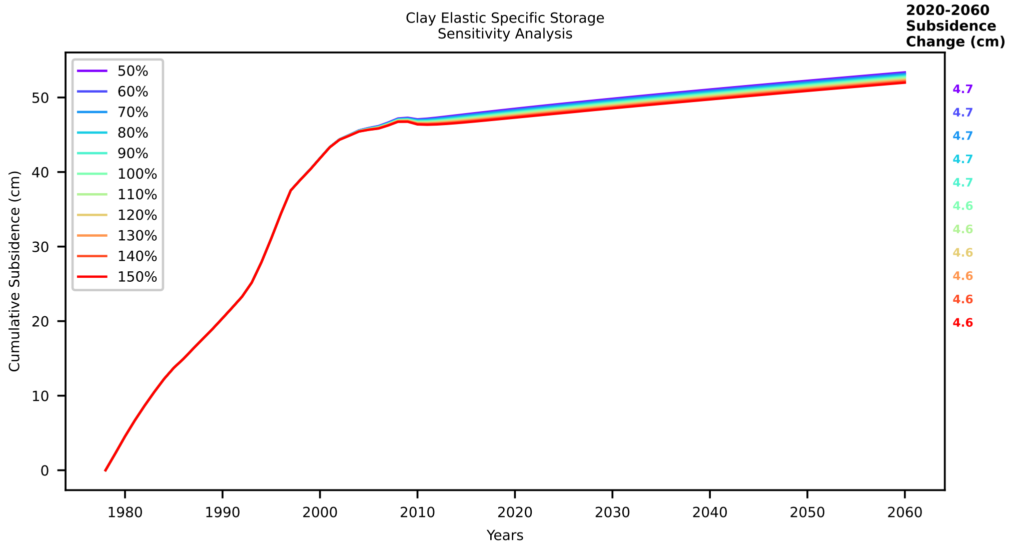

Figure 1.76: Simulated cumulative subsidence for 1978-2060 for well nest (LC)BKK013 after varying the elastic specific storage of clay. For 2020-2060, cumulative subsidence is plotted for several pumping scenarios. The 2020-2060 subsidence change is shown in the upper right hand corner for each sensitivity.

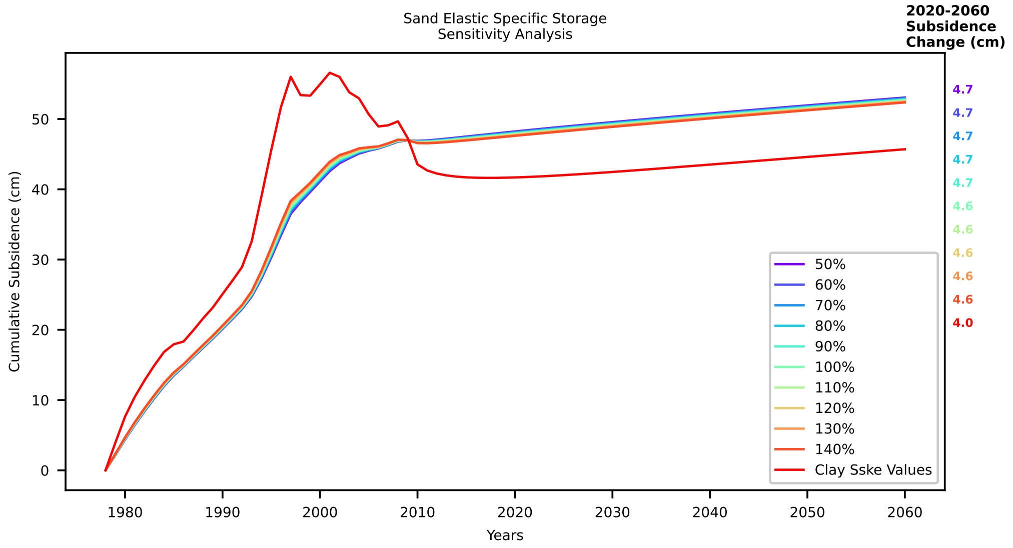

Figure 1.77: Simulated cumulative subsidence for 1978-2060 for well nest (LC)BKK013 after varying the elastic specific storage of sand. For 2020-2060, cumulative subsidence is plotted for several pumping scenarios. The 2020-2060 subsidence change is shown in the upper right hand corner for each sensitivity.

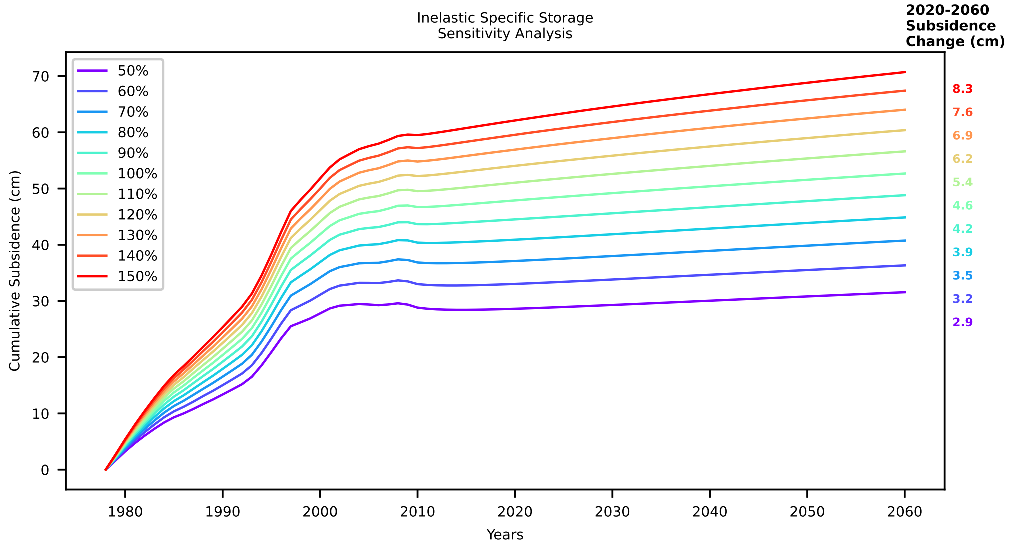

Figure 1.78: Simulated cumulative subsidence for 1978-2060 for well nest (LC)BKK013 after varying the inelastic specific storage of clay. For 2020-2060, cumulative subsidence is plotted for several pumping scenarios. The 2020-2060 subsidence change is shown in the upper right hand corner for each sensitivity.

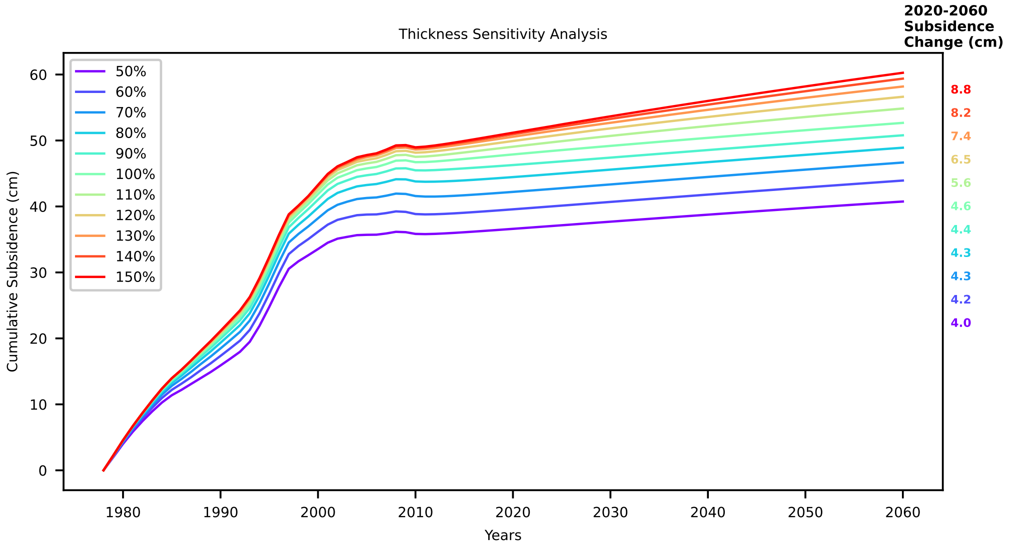

Figure 1.79: Simulated cumulative subsidence for 1978-2060 for well nest (LC)BKK013 after varying the thickness of each layer. For 2020-2060, cumulative subsidence is plotted for several pumping scenarios. The 2020-2060 subsidence change is shown in the upper right hand corner for each sensitivity.
